# COVID-19 in Pregnant Women and Neonates: A Systematic Review of the Literature with Quality Assessment of the Studies

**DOI:** 10.3390/pathogens9060485

**Published:** 2020-06-18

**Authors:** Giulia Trippella, Martina Ciarcià, Marta Ferrari, Chiara Buzzatti, Ilaria Maccora, Chiara Azzari, Carlo Dani, Luisa Galli, Elena Chiappini

**Affiliations:** 1 Post-Graduate School of Pediatrics, Anna Meyer Children’s University Hospital, Department of Health Sciences, University of Florence, 50100 Florence, Italy; giulia.trippella@unifi.it (G.T.); martina.ciarcia@unifi.it (M.C.); marta.ferrari@unifi.it (M.F.); chiara.buzzatti@unifi.it (C.B.); ilaria.maccora@unifi.it (I.M.); 2 Division of Pediatric Immunology, Department of Health Sciences, University of Florence and Meyer Children’s Hospital, 50100 Florence, Italy; chiara.azzari@unifi.it; 3 Department of Neurosciences, Psychology, Drug Research and Children’s Health, University of Florence, 50100 Florence, Italy; carlo.dani@unifi.it; 4 Division of Pediatric Infectious Disease, Anna Meyer Children’s University Hospital, Department of Health Sciences, University of Florence, 50100 Florence, Italy; luisa.galli@unifi.it

**Keywords:** COVID-19, SARS-CoV-2, pregnancy, neonates

## Abstract

The SARS-CoV-2 virus emerged in December 2019 and then spread globally. Little is still known about the impact of COVID-19 on pregnant women and neonates. A review of the literature was performed according to the PRISMA guideline recommendations, searching the MEDLINE and EMBASE databases. Studies’ quality assessments were performed using the JBI Critical Appraisal Checklist. A total of 37 studies were included, involving 275 pregnant women with COVID-19 and 248 neonates. The majority of pregnant women presented with mild to moderate symptoms, only 10 were admitted in the ICU, and one died. Two stillbirths were reported and the incidence of prematurity was 28%. Sixteen neonates were tested positive for SARS-CoV-2 by RT-PCR, and nine of them were born from mothers infected during pregnancy. Neonatal outcomes were generally good: all the affected neonates recovered. RT-PCR for SARS-CoV-2 yielded negative results on amniotic fluid, vaginal/cervical fluids, placenta tissue, and breast milk samples. SARS-CoV-2 infection in pregnant women appeared associated with mild or moderate disease in most cases, with a low morbidity and mortality rate. The outcomes of neonates born from infected women were mainly favorable, although neonates at risk should be closely monitored. Further studies are needed to investigate the possibility of vertical transmission.

## 1. Introduction

The first case of coronavirus disease 2019 (COVID-19), caused by severe acute respiratory syndrome coronavirus 2 (SARS-CoV-2), was described in Wuhan, Hubei Province, China, in December 2019. Since then, COVID-19 has been rapidly spreading all over the world, which led the World Health Organization (WHO) to declare the outbreak of COVID-19 as a pandemic on 11 March 2020 [1].

SARS-CoV-2 is a positive-sense single-stranded RNA (+ssRNA) virus. Coronaviruses (order Nidovirales, family Coronaviridae, and subfamily Orthocoronavirinae) include four coronavirus genera (α,β,γ,δ): human coronaviruses (HCoVs) are classified under α-CoV and β-CoV. SARS-CoV-2 is a β-CoV. Seven coronaviruses are capable of infecting humans, four of them (HCoV-OC43, HCoV-HKU1, HCoV-229E, HCoV-NL63) causing mild respiratory infections, while three, severe acute respiratory syndrome coronavirus (SARS-CoV), Middle East respiratory syndrome coronavirus (MERS-CoV), and SARS-CoV-2, have been associated with severe respiratory diseases, with a high fatality rate [2]. Despite sharing a lot of characteristics with them, most of the information regarding transmission, immune system response, and susceptibility among subgroups of populations is still unclear. Indeed, potentially vulnerable groups deserve special consideration. Pregnant women and neonates are categories of major interest, as they represent a challenge for public health care. Pregnant women have a unique and dynamic immune state during the different stages of pregnancy. It is well known that the hormonal state and the reduced chest expansion increase pregnant women’s risk of respiratory infections [3]. Furthermore, data regarding the previous epidemic coronaviruses, SARS-CoV and MERS-CoV, as well as data regarding influenza viruses, showed that pregnant women and their neonates are exposed to a higher risk of poor outcomes [4]. Little is known about the impact of COVID-19 on pregnancies, perinatal, and neonatal outcomes. The possibility of vertical transmission is still unknown. Although studies on pregnant women and neonates are increasing, most of them are case reports or case series with small population samples and conflicting results.

We performed a systematic review of the literature to provide a comprehensive overview of all the available data regarding clinical features, outcomes, and management of pregnant women with COVID-19. Moreover, we collected data on neonates born from mothers with COVID-19 and neonates with a post-natal diagnosis of COVID-19, analyzing the likelihood of vertical transmission. Finally, we discussed the current management strategies across different countries regarding maternal and neonatal care, which appeared particularly heterogeneous, especially on the type of delivery, isolation, and feeding of neonates.

## 2. Materials and Methods

### 2.1. Study Design and Search Strategy

We performed a systematic review of the literature, according to the Preferred Reporting Items for Systematic Reviews and Meta-analyses (PRISMA) guideline recommendations [5]. We searched the MEDLINE and EMBASE databases from 1 December 2019 to 18 April 2020. We did not restrict the research for language.

Search terms for the MEDLINE database were: (“COVID-19”[All Fields] OR “severe acute respiratory syndrome coronavirus 2”[Supplementary Concept] OR “severe acute respiratory syndrome coronavirus 2”[All Fields] OR “2019-nCoV”[All Fields] OR “SARS-CoV-2”[All Fields] OR “2019nCoV”[All Fields] OR “2019 coronavirus disease” OR ((“Wuhan”[All Fields] AND (“coronavirus”[MeSH Terms] OR “coronavirus”[All Fields])) AND 2019/12[PDAT]: 2030[PDAT])) AND (child* OR pediatri* OR paediatri* OR infan* OR newborn* OR neonate* OR pregnan* OR breastfeed* OR fetal OR fetus OR obstetric* OR “transplacental transmission” OR “placental transmission” OR “vertical transmission” OR intrauterine OR perinatal).

Search terms for the EMBASE database were: (‘covid-19’ OR ‘severe acute respiratory syndrome coronavirus 2’/exp OR ‘severe acute respiratory syndrome coronavirus 2’ OR ‘2019-ncov’ OR ‘sars-cov-2’ OR ‘2019ncov’ OR ‘2019 coronavirus disease’ OR (‘Wuhan’ AND (‘coronavirus’/exp OR ‘coronavirus’))) AND [1-12-2019]/sd AND (child* OR pediatri* OR paediatri* OR infan* OR newborn* OR neonate* OR pregnan* OR breastfeed* OR fetal OR fetus OR obstetric* OR ‘transplacental transmission’ OR ‘placental transmission’ OR ‘vertical transmission’ OR intrauterine OR perinatal).

We performed the research also on Google Scholar and Medrxiv, and finally the references of important articles were crosschecked.

### 2.2. Study Eligibility and Quality Assessment

The articles included provide epidemiological, clinical, diagnostic, and/or therapeutic data about pregnant women or neonates with COVID-19. At first, we screened titles and abstracts to discover eligible studies, and then we analyzed all full texts for the final evaluation. Two investigators (MF, CB) independently reviewed and evaluated every study.

We considered a study eligible when the following criteria were met: (1) population were pregnant women and/or neonates; (2) a diagnosis of COVID-19 was made with specified diagnostic criteria; and (3) epidemiological, clinical, diagnostic and/or therapeutic data were reported.

Exclusion criteria were: (1) not relevant topic (not appropriate population or not appropriate outcome) (2) non-original studies (e.g., literature reviews, guidelines, duplicate articles, comments); and (3) studies not reporting useful clinical data about patients.

Considering the relevance of the topic, we decided to include as many studies, fitting the eligibility criteria and presenting original data, as possible, comprising non-peer-reviewed publications. Nevertheless, we carefully assessed and described the quality of every study and verified that there was no overlap in the population sample. Specifically, we crosschecked the settings, the population samples, and the period of admission of the patients, finding several studies performed in the same setting and the same period. We excluded all the articles with confirmed (by contacting the authors) or suspected dataset overlap.

The quality of the eligible studies was evaluated by two authors independently (MC, GT), using different methods according to the study design: The Joanna Briggs Institute (JBI) Critical Appraisal Checklist for Case Reports [6], and the Joanna Briggs Institute (JBI) Critical Appraisal Checklist for Case Series [7]. We planned to use the Newcastle-Ottawa Quality Assessment Form [8] for the analytic observational studies, however, we did not identify articles that properly fitted this definition.

### 2.3. Data Extraction and Definitions

Data were collected and entered into an electronic database (Microsoft Corporation, 2018 Microsoft Excel, Redmond, WA, USA).

From each study, we selected information regarding study design, date of publication, country of origin, setting, characteristics of the population sample, objective of the study, and outcome measured. As a measured outcome, we considered any clinical data described and analyzed. For mothers, we collected data on age, comorbidities, symptoms, gestational age at symptom onset and delivery, imaging and laboratory testing results, administered therapy, and type of delivery. Regarding neonates, we collected data on birth weight, Apgar score (Appearance, Pulse, Grimace, Activity, Respiration), isolation, feeding, symptoms, imaging and laboratory testing results, and administered therapy. Maternal and perinatal outcomes were evaluated and the likelihood of vertical transmission of the virus was analyzed.

We considered articles following the diagnostic criteria for COVID-19 based on the New Coronavirus Pneumonia Prevention and Control Program (Trial Fifth Edition) issued by the National Health Commission of China [9]. The above-mentioned diagnostic criteria of COVID-19 include (1) typical chest CT imaging of patchy shadowing and ground-glass opacity, and (2) positive in reverse transcription-polymerase chain reaction (RT-PCR) tests for SARS-CoV-2.

Moreover, considering the risk of false-negative results of laboratory tests (possibly related to low virus titers, inappropriate swabbing sites, or variability on laboratory test performance) [10] and the limited test capacities during a period of overloaded healthcare systems, we decided to include also studies using extended diagnostic criteria. Indeed, some authors included patients with negative RT-PCR tests, but typical symptoms and chest CT imaging, in association with suggestive clinical data (blood tests showing leukopenia or lymphocytopenia, exclusion of other causes of respiratory infections).

### 2.4. Statistical Analysis

Statistical analysis was performed using Microsoft Excel (Microsoft Corporation, 2018). Categorical variables were expressed as the number of cases (N) and percentages (%). Continuous variables were expressed as the mean with standard deviation (SD).

## 3. Results

### 3.1. Included Studies

**Figure 1:**
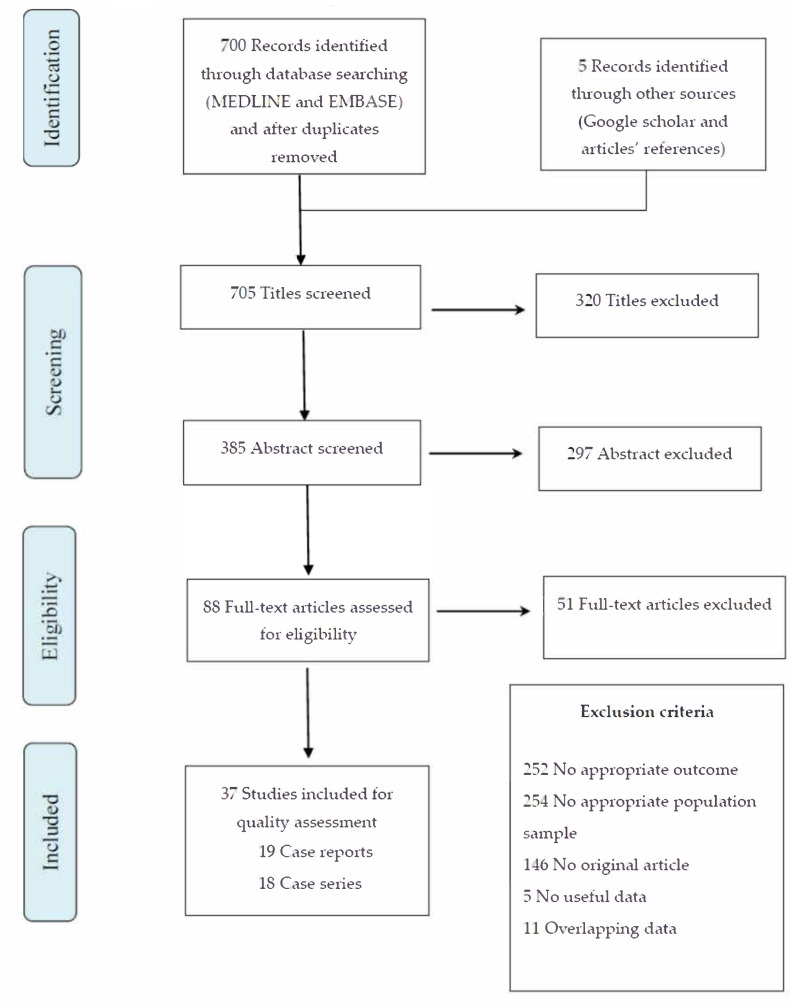
Flow chart of the study selection.

The selection process is shown in Figure 1.

Our search strategy identified 700 articles, after duplicate removal. The Google Scholar research and the articles’ references crosscheck identified five articles. The abstracts and titles screening found 88 studies that met the inclusion criteria. After the full-text analyses, 51 of these studies were excluded (Supplementary Materials). Thirty-seven studies were eligible according to our criteria and were included in the review: 19 case reports ([11,12,13,14,15,16,17,18,19,20,21,22,23,24,25,26,27,28,29]) and 18 case series ([30,31,32,33,34,35,36,37,38,39,40,41,42,43,44,45,46,47]).

Characteristics of the included studies are reported in Table 1.

### 3.2. Quality Assessment

Only five case reports ([12,13,20,24,26]) completely fulfilled the checklist criteria, scoring 7 out of 8 on the JBI Critical Appraisal Checklist for Case Reports; we considered not applicable the seventh question of the checklist. In the remaining 17 articles, we found missing information or unclear information. Specifically, in five articles, the setting was not specified, while some articles did not report data about the pregnancy/birth (n = six), about the pregnant woman (n = one), about the neonate (n = four), about the administered treatment (n = eight), or the outcome (n = three).

Out of 17 analyzed case series, only 3 ([30,40,44]) completely fulfilled the checklist criteria, scoring 9 out of 10 on the JBI Critical Appraisal Checklist for Case Series. The 10th question, about statistical analysis, was appropriate for only one study [33], in which authors performed a comparison between two groups. In the remaining 12 articles, we found missing information in one or more of the investigated aspects. In three studies, patients’ characteristics were not evaluated in a standard way (some tests were not performed on all the population sample), and in five studies, the condition was not identified with valid methods for all patients (some maternal diagnoses were not laboratory-confirmed). Four case series described patients from different hospitals, without specifying if the inclusion was consecutive. Demographic and clinical data of the participants were not reported or were missing in one and four studies, respectively. Maternal and/or neonatal outcomes or follow-up information were not reported in nine articles. Finally, demographic data were not reported in the four case series.

**Figure 2:**
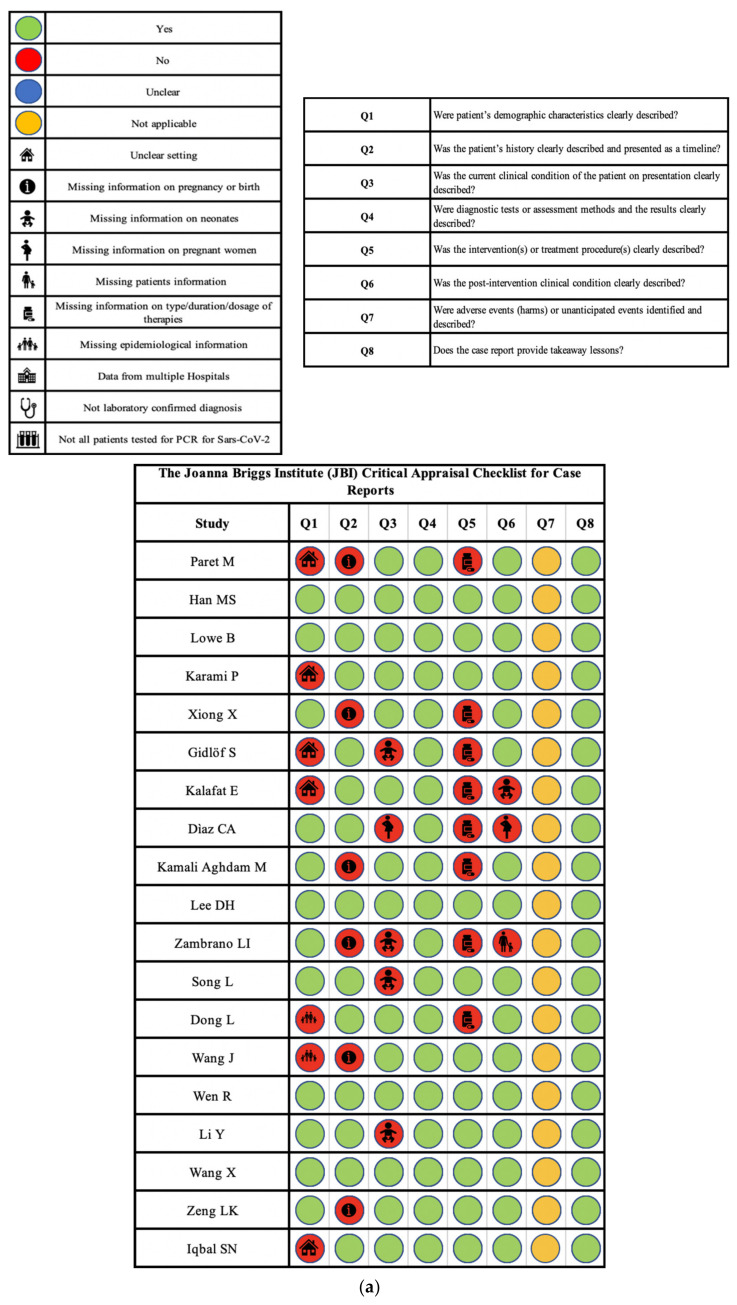

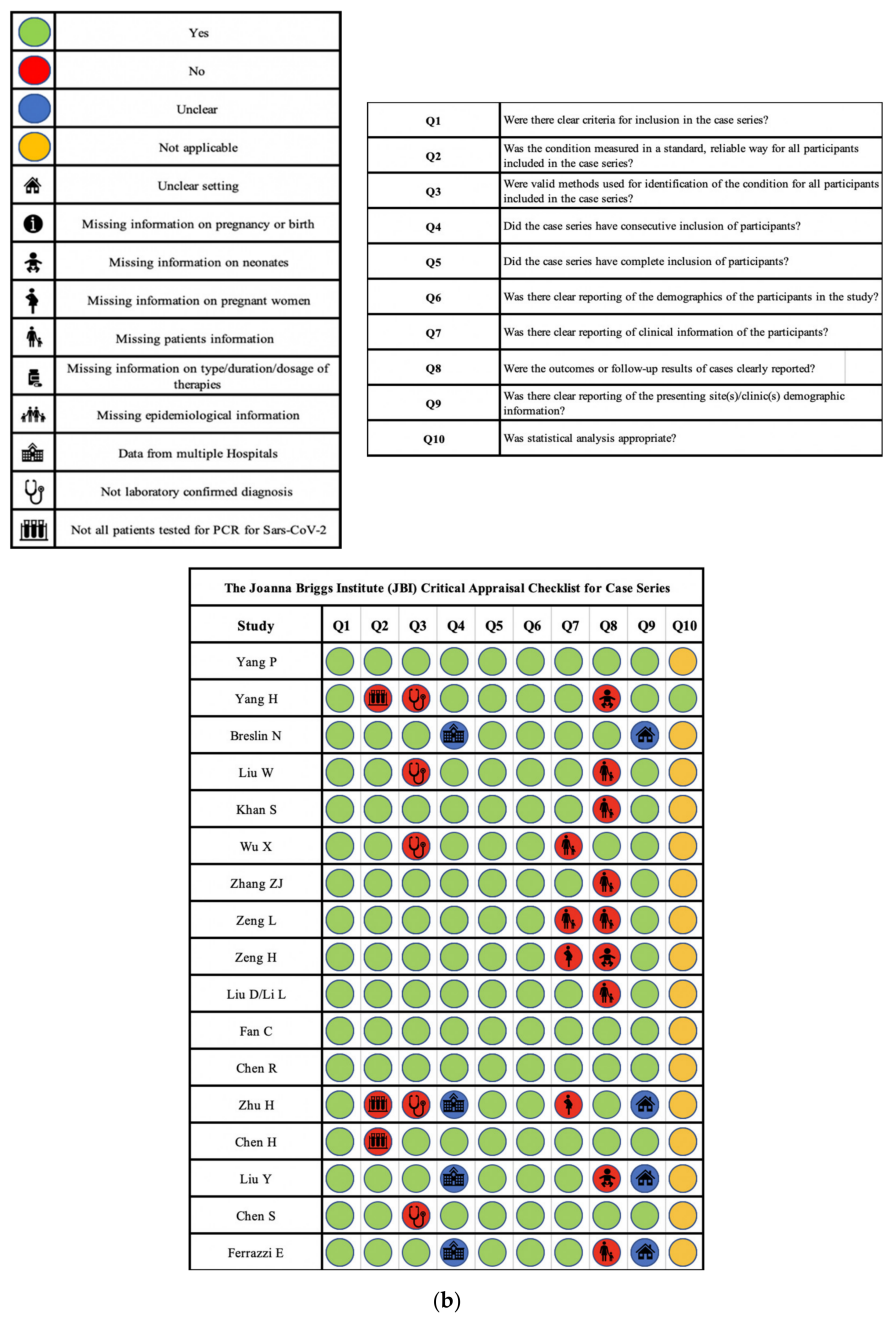
Quality assessment of the included studies. (**a**) The Joanna Briggs Institute (JBI) Critical Appraisal Checklist for Case Reports; (**b**) The Joanna Briggs Institute (JBI) Critical Appraisal Checklist for Case Series.

The quality assessment of the studies is reported in Figure 2.

### 3.3. Maternal Characteristics and Outcomes

The included articles described 275 pregnant women affected with COVID-19, 181 from China, and 94 from other countries. RT-PCR for SARS-CoV-2 was positive for 260 women (95%) on pharyngeal/nasopharyngeal swab, while 15 women were diagnosed using extended diagnostic criteria, based on clinical and radiological features, and excluding other causes of symptoms.

Out of 275 pregnant women, 239 gave birth: 3 women voluntarily decided to terminate their pregnancy in the first trimester and 33 women were still pregnant when the articles were published. Gestational age at delivery was reported for 208 patients: 160 (77%) delivered at term (≥37 weeks of gestation) and 48 (23%) had a preterm delivery (<37 weeks of gestation).

In 179 (75%) cases, the type of delivery was a cesarean section, which was performed in an emergency in 27 cases (11%). Sixty (25%) women delivered vaginally. Considering the available data, indications for cesarean section were often obstetric reasons (e.g., placenta previa, repeat cesarean, the arrest of descending, the arrest of dilation, failed labor induction, fetal distress); however, in some cases, the diagnosis of COVID-19 was considered itself an indication to perform surgical delivery, especially when a worsening of maternal respiratory conditions occurred or when mothers needed antiviral treatment.

Some of the articles reported the presence of comorbidities, most likely not related to COVID-19, such as obesity (n = 27), gestational diabetes (n = 10), hypertension (n = 10), asthma (n = 8), pre-eclampsia (n = 5), anemia (n = 5), diabetes mellitus (n = 3), hypothyroidism (n = 3), placenta previa (n = 3), hepatitis B (n = 2), cholecystitis (n = 2), placental abruption (n = 1), cephalopelvic disproportion (n = 1), and thalassemia (n = 1).

Symptoms were clearly described in 269 women out of 275 (98%). The most frequent symptoms were fever (155, 58%) and cough (98, 36%); thirty-seven (14%) patients complained of myalgia, malaise, or fatigue, 28 (10%) patients developed dyspnea, and only 9 (3%) presented with mild respiratory symptoms (sore throat, nasal congestion); a small number of patients (9, 3%) presented with gastrointestinal symptoms, such as diarrhea or abdominal pain. Twenty-two women (8%) were asymptomatic. In most of the cases, symptoms began before delivery (1 to 38 days before, mean 9.26 days, SD 8.86).

Laboratory findings were reported for 108 women out of 275 (39%) and revealed elevated C-reactive protein (CRP) in 52 cases (48%), lymphopenia in 31 cases (29%), and elevated liver enzymes in 9 cases (8%). Serological tests for SARS-CoV-2 were performed in nine RT-PCR-positive patients: all of them had positive IgG and six out of nine had positive IgM for SARS-CoV-2.

As diagnostic imaging, 171 patients underwent chest CT, which showed a typical pattern of viral pneumonia in 162 cases (95%), while 9 (5%) were normal. Chest X-rays were reported for 48 women and were all consistent with the diagnosis of viral pneumonia.

Regarding medical treatments, reported data are extremely heterogeneous, depending on the patients’ clinical presentation. Furthermore, hospital protocols varied among different countries and changed rapidly over time according to the emergence of new evidence.

Overall, the majority of pregnant women developed a mild or moderate illness; twenty-two women did not develop symptoms. However, complications were reported. Considering the reported data, 36 patients required oxygen therapy and 10 patients required the intensive care unit (ICU). Five women required invasive ventilation and one woman needed extracorporeal membrane oxygenation (ECMO) and hemodialysis. The latter was a 31-year-old Chinese woman at 35 + 2 weeks’ gestation, admitted to hospital because of fever and dyspnea, who developed severe acute respiratory distress syndrome (ARDS), septic shock, and multiple organ dysfunction syndrome (MODS). Cesarean section was performed, but the newborn underwent intra-uterine asphyxia and died. The patient received invasive ventilation, continuous renal replacement therapy, and ECMO and was treated with antiviral therapy (lopinavir-ritonavir and ribavirin) and transfusion of convalescent plasma [47]. The follow-up of this patient is described by Zhang B et al. [48], who showed that she finally recovered and was discharged from the hospital. One of the patients who required intensive treatment had a fatal outcome. [14] She was a 27-year-old mother at 30 + 3 weeks’ gestation, admitted to hospital for progressive respiratory distress. The day after the admission, she spontaneously gave birth to a cyanotic neonate with an Apgar score of 0 (at first and fifth minute) not responding to neonatal cardiopulmonary resuscitation (CPR). After delivery, there was a clinical deterioration; she was treated with lopinavir-ritonavir, oseltamivir, hydroxychloroquine, meropenem, and vancomycin and received corticosteroid pulse therapy, emergency plasmapheresis, and invasive ventilation. Despite treatment, the patient died as a result of multiple organ failure (MOF).

Data regarding the included pregnant women are reported in Table 2.

### 3.4. Neonatal Characteristics and Outcomes

The articles analyzed in our review described 248 neonates. Data on the gestational age of the neonates were available for 196 neonates, 72% of whom were at term (≥37 weeks of gestation). The average birth weight was 2914 (±573) g. Apgar scores were reported over 7 at the first minute and/or at the fifth minute in 97% of cases, while five (3%) were under 7 at the fifth minute.

Among the 248 included neonates, 191 neonates born from mothers with COVID-19 were tested with RT-PCR for Sars-CoV-2 on throat/nasopharyngeal/anal swab; in 28 cases (15%), also specimens were analyzed (urine, blood, feces samples). Twenty-six patients underwent serological tests (IgM, IgG) for SARS-CoV-2.

Out of the 191 tested neonates, 175 (92%) were negative. However, among the negative neonates, some authors reported clinical symptoms. Symptoms most frequently described were: fever (n = 4), mild respiratory symptoms (rhinitis) (n = 1), respiratory distress (n = 16), and gastrointestinal symptoms (n = 8). Zhu et al. [45] reported a high incidence of complications on a population sample of 10 negative neonates: respiratory distress (n = 6), fever (n = 2), thrombocytopenia with liver failure (n = 2), tachycardia (n = 1), vomiting (n = 1), pneumothorax (n = 1), refractory shock with subsequent MOF, and disseminated intravascular coagulation (DIC) until death (n = 1). Nevertheless, this series included cases from five different hospitals (in unclear settings) and all the sick babies were preterm neonates (range of gestational age: 31–35 + 6 weeks of gestation). One case of neonatal asphyxia was reported by Zeng et al. [38] Two stillbirths were described by Karami et al. [14] and Liu et al. [47], both from mothers with severe SARS-CoV-2 pneumonia requiring intensive treatment; in one case, the mother had a fatal outcome (see above).

Sixteen of the tested neonates yielded SARS-CoV-2-positive results (Table 3).

Fourteen of them were RT-PCR-positive on the nasopharyngeal swab, and two were RT-PCR-positive on the anal swab. Among them, nine were born from mothers with COVID-19 diagnosed during pregnancy or in the immediate post-delivery period (within 36 h). The other seven positive neonates were born from asymptomatic mothers, and not tested, during pregnancy, who were diagnosed during the first month of life (range 8–27 days after birth). Among the RT-PCR-positive neonates, symptoms were reported in 14 cases. Fever was the most frequent symptom (n = nine), followed by gastrointestinal symptoms (n = five), respiratory distress (n = five), and mild respiratory symptoms (cough, rhinitis) (n = three). One neonate required non-invasive respiratory support and two required mechanical ventilation. All neonates had a good outcome. No cases of death in SARS-CoV-2-positive neonates are described in the literature. Zeng et al. [38] reported a positive neonate born at 31 + 2 weeks’ gestation by C-section because of fetal distress, who required resuscitation at birth (Apgar score was reported as 3 at the first minute and 4 at the fifth), treatment with antibiotics, caffeine, and non-invasive respiratory support. On day 2, nasopharyngeal and anal swabs resulted in positive for SARS-CoV-2. However, he also suffered from Enterobacter sepsis with subsequent thrombocytopenia, leukocytosis, and coagulopathy which progressively improved after antibiotic treatment.

Regarding the laboratory findings, data were reported for seven positive neonates and showed: thrombocytopenia (n = two), elevated PCT (n = one), lymphocytosis (n = one), leukocytosis (n = two), and lymphocytopenia (n = one); five neonates had normal laboratory tests.

Imaging diagnostic tests were performed on 11 positive neonates: 5 neonates underwent chest CT, while 8 neonates underwent chest X-ray (2 underwent both chest CT and chest X-ray). All chest CTs showed anomalies (most frequently increased lung marking), six chest X-rays were consistent with pneumonia, two were negative.

The majority of neonates (125, 73%) were separated from the infected mothers immediately after delivery and were isolated for at least 14 days. Data about feeding were reported for 56 cases; 68% of neonates were fed with formula. However, all RT-PCR tests for SARS-CoV-2 (25 specimens) performed on breast milk yielded negative results.

To evaluate the possibility of vertical transmission, some authors tested SARS-CoV-2 RT-PCR in other samples. Amniotic fluid (n = 24), cord blood (n = 30), placenta samples (n = 6), and cervical/vaginal fluids (n = 7) were tested in 14% of the 248 included cases. All of these samples yielded negative results.

Data regarding the included neonates are reported in Table 4.

## 4. Discussion

### 4.1. SARS-CoV-2 Infection in Pregnant Women

Since December 2019, when the first cases of COVID-19 pneumonia were identified in Wuhan, few narrative reviews ([4,49,50]) and only one systematic review [51] were published, evaluating the clinical outcome, diagnostic, and therapeutic data in pregnant women and neonates with COVID-19.

This is the most comprehensive systematic review, with a quality assessment of the included studies, describing pregnant women and neonates with COVID-19. A total of 37 studies were retrieved, involving overall 275 pregnant women and 248 neonates.

Most cases in the literature showed that pregnant women with SARS-CoV-2 infection and their neonates had better outcomes than expected. Specifically, the reported outcomes were better than the previously reported for patients affected with SARS or MERS. Being affected with SARS and MERS during pregnancy has been associated with severe prognosis, both for mothers and neonates. [4]

However, no vertical transmission has been identified among pregnant women infected with SARS-CoV and MERS-CoV [52]. Therefore, it is possible to speculate that the worse neonatal outcome was related to the poor clinical conditions of the mother during pregnancy and delivery, rather than being caused by vertical transmission of the infection. Nevertheless, so far, data are still limited.

Our review demonstrated that pregnant women with COVID-19 generally presented with mild symptoms, mostly fever and cough, a significant number of patients remained asymptomatic, and only a few cases developed dyspnea requiring oxygen therapy or admission in ICU for intensive treatment. One patient needed intensive care because of ARDS and MODS requiring intubation, mechanical ventilation, and the support of ECMO, however, she fully recovered. [47,48] Only one patient, out of the 275 described, had a fatal outcome. [14]

According to Chen et al. [41] and Wu et al. [36], clinical symptoms and chest CT features of COVID-19 in pregnant women were similar to those of non-pregnant women. Laboratory findings, as well, appeared analogous to the ones described in non-pregnant SARS-CoV-2-infected patients: elevated levels of C-reactive protein (48%), mild lymphocytopenia (29%), and elevated levels of liver transaminase (8%) were the most frequently recorded abnormalities.

In our review, a high rate of pregnant women (62%) underwent a chest CT, which was positive for viral pneumonia in 95% of cases. Chest CT imaging has been reported as superior to RT-PCR in sensitivity for early diagnosis of COVID-19 [53] and it is essential even for a severity assessment and follow-up of SARS-CoV-2 pneumonia, with higher sensitivity than chest X-rays, especially in clinically diagnosed patients [54]. Hence, it is important to choose low-dose techniques for diagnostic imaging, which are safer for the fetus, and to protect the mother’s abdomen whenever possible, to minimize ionizing radiation exposition. [54]

The only Italian study included in this review reported a different approach to patients regarding diagnostic imaging: patients did not undergo any chest CT, they received, instead, a confirmative chest X-ray, which was consistent with the diagnosis of viral pneumonia in all of the 42 cases [39]. Further studies are needed to define the appropriate diagnostic techniques in pregnant women.

A high incidence rate of premature delivery is reported in some studies [23,26,47]. We confirmed these data, with a percentage of preterm delivery of 28% among the reported cases, although most cases were late-preterm. The global preterm birth rate (percentage of all births delivered <37 completed weeks of gestation) was reported to be approximately 10.6% [55] and 10% [56] in the USA in 2014 and 2018, respectively. Among included studies, causes of preterm delivery were not always clarified. The most frequently reported causes of preterm emergency cesarean section were: fetal distress, worsening of mother’s respiratory symptoms [26,28], or the need to start antiviral treatment [46]. Indeed, causes of preterm delivery might not always be directly related to SARS-CoV-2 infection and further studies are needed to explain the correlation between pre-term delivery and SARS-CoV-2 infection.

Regarding the type of delivery, C-section was performed in most cases (75%). However, data showed that spontaneous vaginal delivery, when feasible according to clinical mothers’ conditions, was not associated with poorer outcomes either for mothers or for neonates. It is possible to speculate that, as experienced in the management of pregnant women with COVID19 increases, the rate of cesarean section will decrease.

### 4.2. SARS-CoV-2 Infection in Neonates

Our review reported 248 neonates born from infected mothers. Most of the cases (92%) were healthy babies at birth with an Apgar score of 8–10 and RT-PCR-negative for SARS-CoV-2. No spontaneous abortion or congenital anomalies were reported. Even neonates who resulted positive for SARS-CoV-2 infection showed a mild clinical course of the disease, with a good outcome.

We identified nine cases of neonates, born from mothers with a diagnosis of COVID-19 during pregnancy or in the immediate post-partum period, who were tested positive for SARS-CoV-2. Seven of them were isolated from the mothers immediately after birth and breastfed with formula, and in two cases, they were breastfed without the surgical mask during the first hours of life because the mothers were found positive after delivery (within 36 h). In these cases, it is difficult to speculate on how the infection was acquired: even though these neonates had positive results of the RT-PCR testing for SARS-CoV-2, the tests were not performed immediately at birth, but mostly 36–48 h after birth. Hence, a hospital-acquired infection could not be ruled out. In one case, the first test for SARS-CoV-2 was doubtful a few hours after birth and yielded a positive result on the third day of life [39].

Furthermore, the possibility of false-positive results could not be excluded. Indeed, one of the authors reported a false-positive result of RT-PCR testing for SARS-CoV-2: one neonate had positive RT-PCR in the throat swab at first, but the second test on the same swab was negative. Moreover, even RT-PCR on amniotic fluid and umbilical cord blood yielded a negative result [34].

Additionally, among the included studies, all RT-PCR testing for SARS-CoV-2 performed on amniotic fluid, cord blood, placental sample, and cervical/vaginal fluid yielded a negative result.

The other seven positive reported neonates were born from mothers without a COVID-19 diagnosis during pregnancy and were admitted to hospital from 8 to 27 days after birth. In these cases, it is possible to hypothesize a horizontal transmission, probably due to droplet transmission from infected family members.

Dong et al. [22] and Zeng et al. [42] reported three neonates with negative RT-PCR for SARS-CoV-2, but elevated IgM antibody for SARS-CoV-2 after birth, supporting the hypothesis of vertical infection. However, as Kimberlin et al. [57] suggested, the diagnosis of congenital infection should not be confirmed only by IgM detection because of the possibility of cross-reactivity with subsequent false-negative and false-positive results.

To the best of our knowledge, vertical transmission is unlikely, although the few positive neonates from infected mothers described in the literature do not allow to rule it out definitively. Further studies, focusing also on the detection of the virus on a specimen such as amniotic fluid, cord blood, placental sample, and cervical/vaginal fluid would need to confirm that.

### 4.3. International Guidelines on the Management of Infected Pregnant Women and Neonates

In our review, most of the Chinese neonates from positive mothers were isolated until the mothers were negative at RT-PCR for SARS-CoV-2 and were fed with formula because of the risk of transmission through respiratory droplets and maternal contact. Thus, for neonates born to mothers suspected for or diagnosed with SARS-CoV-2 infection, the Chinese expert consensus on the perinatal and neonatal management for the prevention and control of the 2019 novel coronavirus infection does not recommend mother–baby contact and recommends to avoid feeds with breast milk until a negative result for SARS-CoV-2 on milk has been obtained. As the Chinese Paediatrics COVID-19 Working Group suggests, the indication is unbalanced without any analysis of the global risks and benefits of not breastfeeding compared with those of neonatal infection. Authors who collected and tested breast milk samples from mothers with COVID-19 before delivery showed that all samples tested were negative. [34,41] Moreover, the primary concern is not only the virus’s transmission through breast milk, but whether mothers’ respiratory droplets could infect the baby during breastfeeding.

The World Health Organization, as well as United Nations Children’s Fund (UNICEF), underline that breast milk is the best source of feed even in infants born from mothers with suspected or confirmed SARS-CoV-2 infection, as well as all infants. Therefore, considering also the generally mild course in infants and young children, WHO encouraged to touch and hold the baby, breastfeed safely with good respiratory hygiene, hold the baby skin-to-skin, and share a room with the child when the mother’s clinical condition permits it.

The American College of Obstetricians and Gynaecologists (ACOG) [58], The Royal College of Obstetricians and Gynecologists (RCOG) [59], and the Italian Society of Neonatology endorsed by the Union of European Neonatal and Perinatal Societies [60] suggest that in case of a paucisymptomatic or asymptomatic mother with identified or suspected SARS-CoV-2 infection at delivery, direct breastfeeding and room-sharing is possible, with rigorous measures of infection control such as mother’s hands’ disinfection before holding the neonates and wearing a face mask during breastfeeding.

Ferrazzi et al. [39] reported the largest case series of neonates breastfed from mothers with COVID 19 (n = 11). All neonates had a favorable outcome, however, there are no available data regarding long-term follow-up.

Currently, the paucity of the studies’ results makes it hard to define the best strategy for the management of infected mothers with their infants during the post-natal period. Study results pending, it is likely that the benefits of breastfeeding and early mother–child contact are greater than the neonatal risk of SARS-CoV-2 infection.

### 4.4. Limitations of the Study

This review has several limitations. EMBASE, Medline, and Google Scholar were the only databases searched, hence relevant publications reported in other databases might have been missed. The search methodology could have introduced selection bias. Results should be interpreted cautiously due to the high heterogeneity among the included studies, and the fact that not all included articles were peer-reviewed articles. Finally, it is important to stress that the COVID-19 pandemic is still in progress all over the world; the reviewed studies are mostly carried out in China, using patient management and guidelines sometimes different from the ones used in Europe and the USA.

## 5. Conclusions

To the best of our knowledge, this is the biggest review regarding the impact of COVID-19 in pregnant women and neonates reported in the literature. According to our data, pregnant women with COVID-19 mostly presented with mild or moderate symptoms, with a low incidence of serious complications and adverse outcomes. Even the outcome of neonates born from infected mothers appeared mostly favorable. However, despite having a big population sample (275 pregnant women and 248 neonates), the information often derived from low-quality studies, which are case reports or case series. No observational analytic studies or clinical trials are available in the literature. Hence, although the data are reassuring, they must be confirmed by larger and high-quality studies.

Vertical transmission of SARS-CoV-2 was not detected in the majority of the reported cases, however, few cases of neonates with positive RT-PCR after birth are described. Hence, it is still not possible to rule out the vertical transmission.

Finally, we addressed that the creation of international registers, with easily available and detailed patient data, would be a useful tool to guide management decisions in a moment of global emergency.

## 6. Key Issues

- SARS-CoV-2 infection in pregnant women appeared associated with mild or moderate disease in the majority of cases, with a low incidence of severe complications and low mortality rates;
- Outcomes of neonates born from infected women were mainly favorable, although neonates at risk should be closely monitored to carry out early intervention for patients with abnormal findings;
- Further studies are needed to investigate the possibility of vertical transmission.

## Figures and Tables

**Table 1:** Characteristics of the included studies.

Author	Design	Setting, Country	Population	Objective	Results
Yang P	Case series	Zhongnan Hospital of Wuhan University, Wuhan, China	7 pregnant women with COVID-19 7 neonates	To describe clinical characteristics of neonates born from pregnant women with SARS-CoV-2	Good outcomes were reported for pregnant women with SARS-CoV-2 and their offspring of neonatal pharyngeal swabs, amniotic fluid samples, and umbilical cord blood samples all tested negative for SARS-CoV-2 by RT-PCR.
Paret M	Case report	Department of Pediatrics, Division of Pediatric Infectious Diseases, New York University Grossman School of Medicine, New York, USA	1 neonate	To describe clinical characteristics of SARS-CoV-2 infection in febrile infants	No adverse outcomes were reported in neonates with SARS-CoV-2 infection. Encourage routine testing of febrile infants for SARS-CoV-2, even if they lack respiratory symptoms.
Li L	Case series	Union Hospital, Tongji Medical College, Huazhong University of Science and Technology, Wuhan, China	4 pregnant women with COVID-19	To describe follow-up characteristics of 4 women already described in the previous article	Pregnant women suffering from pneumonia due to SARS-CoV-2 did not experience severe symptoms or acute respiratory distress syndrome during follow-up (follow-up of Liu D pregnant women)
Han MS	Case report	Seoul National University Children’s Hospital, Seoul, Korea,	1 neonate	To describe the clinical characteristics of COVID-19 in a neonate and her mother; to analyze the viral load of SARS-CoV-2 in clinical specimens from different sources.	SARS-CoV-2 infection did not lead to adverse outcomes in the neonate. SARS- CoV-2 could be shed for a long time in urine and stool.
Yang H	Case series	Maternal and Child Health Hospital of Hubei Province, Tongji Medical College, Huazhong University of Science and Technology, Wuhan, China.	13 pregnant women with COVID-19 14 neonates	To describe the clinical characteristics and outcomes of pregnant women with confirmed SARS-CoV-2 infection	13 Pregnant women with confirmed SARS-CoV-2 pneumonia experienced mild symptoms or were asymptomatic. All neonates tested for SARS-CoV-2 were negative. Pulmonary CT scan can be used to screen SARS-CoV-2 pregnant women in the outbreak area of SARS-CoV-2 infection.
Lowe B	Case report	Gold Coast University Hospital (GCUH), Southport, QLD, Australia	1 pregnant woman with SARS-CoV-2 1 neonate	To describe a vaginal birth in a SARS-CoV-2 mother	Vaginal birth in a SARS-CoV-2 patient occurred without complications Rooming-in post-delivery for SARS-CoV-2 positive parents and breastfeeding appears possible and safe when appropriate precautions are taken.
Breslin N	Case series	Columbia University Irving Medical Center and Allen Hospital, New York, NY, USA	43 pregnant women with COVID-19 18 neonates	To describe the clinical characteristics and outcome of pregnant women with COVID-19 and their neonates	The severity of COVID-19 is similar in pregnant women and non-pregnant adults. Ten women delivered vaginally without any complication. All neonates tested negative for SARS-CoV-2
Liu W	Case series	Tongji Hospital, Tongji Medical College, Huazhong University of Science and Technology, Wuhan, China	19 pregnant women with COVID-19 19 neonates	To describe the clinical characteristics and outcomes of 19 neonates born to mothers suffered from COVID-19	RT PCR for SARS-CoV-2 tested negative on all neonatal throat swabs, gastric fluid, urine, feces, amniotic fluid, and umbilical cord blood. No one showed any clinical, radiologic, or biochemical sign of COVID-19.
Karami P	Case report	Vali-e-asr Hospital, School of Medicine, Zanjan University of Medical Sciences, Zanjan, Iran	1 pregnant woman with COVID-19 1 neonate	To describe a case of maternal and fetus death in a patient with COVID-19	A 27-year-old pregnant woman at 30^+3^ weeks suffering respiratory distress requiring ICU admission and mechanical ventilation. She delivered vaginally after the spontaneous onset of uterine contractions. The neonate was stillborn, with Apgar 0 and did not respond to resuscitation. Lately she developed ARDS, MOF, and finally death.
Khan S	Case series	Renmin Hospital of Wuhan University, Wuhan, China	3 pregnant women with COVID-19 3 neonates	To describe vaginal birth in SARS-CoV-2-positive mothers	Vaginal delivery was uncomplicated in SARS-CoV-2-positive mothers. All the 3 neonates tested negative for SARS-CoV-2. No vertical transmission of SARS-CoV-2 or intrapartum transmission was found.
Xiong X	Case report	Beijing YouAn Hospital, Beijing, China	1 pregnant woman with COVID-19 1 neonate	To describe a vaginal birth in a SARS-CoV-2 positive mother	Vaginal birth in a SARS-CoV-2-positive mothers was uncomplicated. Neonate tested negative for SARS-CoV-2. No vertical transmission of SARS-CoV-2 or intrapartum transmission was found.
Wu X	Case series	Central Hospital of Wuhan, Wuhan, China	23 pregnant women with COVID-19 21 neonates	To describe the clinical characteristics and to study chest CT images in SARS-CoV-2-infected pregnant women	Pregnant and non- pregnant women with SARS-CoV-2 presented similar radiological findings and clinical characteristics. Four of the 21 neonates were negative for SARS-CoV-2 on RT-PCR, 17 were clinically diagnosed as negative.
Zhang ZJ	Case series	Data from central government and local healthdepartments, China	3 pregnant women with COVID-194 neonates with nucleic acid-confirmed SARS-CoV-2	To describe the clinical characteristics and outcome of neonates with SARS-CoV-2	Compared with adults, neonates showed milder symptoms and better outcomes.
Gidlöf S	Case report	Stockholm, Sweden	1 pregnant woman with COVID-19 2 neonates	To describe the clinical characteristics and outcome of a pregnant woman with COVID-19 and her neonates; to ask for liberal testing for SARS-CoV-2 in high-risk patients.	No evidence of vertical transmission. Normal neonatal outcome.
Kalafat E	Case report	Ankara, Turkey	1 pregnant woman with COVID-19 1 neonate	To describe lung ultrasound role in a pregnant COVID-19 woman	Lung-ultrasound examination is easy and could be useful in pregnant women with COVID-19 All neonatal samples tested negative for SARS-CoV-2 (swabs, cord blood, and placental swab). Mother still in ICU at time of publication.
Alonso DÌaz C	Case report	Madrid, Spain	1 pregnant woman with COVID-9 1 neonate	To describe the clinical characteristics and outcome of a pregnant woman with SARS-CoV-2 and her neonate	The mother was asymptomatic at delivery, then needed mechanical ventilation in ICU. This is a suspicious case of horizontal transmission, as the initial neonatal SARS-CoV-2 test was negative. Neonate showed mild symptoms.
Kamali Aghdam M	Case report	Mousavi Hospital, Zanjan, Iran	1 neonate	To describe the clinical outcome of an infected neonate	The neonate had positive throat swab and normal chest X-ray. He rapidly improved with oxygen therapy, fluids, antibiotics, and antiviral treatment (Oseltamivir). Discharged on the 6th day.
Lee DH	Case report	Daegu Fatimal Hospital, Daegu, South Korea	1 pregnant woman with COVID-19 1 neonate	To describe the preventive measures required for delivery in pregnant women with COVID-19	Mechanical obstruction during labor led to an emergency C-section. Two neonatal throat swab, placental tissue, amniotic fluid sample, and cord blood sample were negative for SARS-CoV-2.
Zambrano LI	Case report	Hospital Escuela of Tegucigalpa, Tegucigalpa, Honduras	1 pregnant woman with COVID-19 1 neonate	To describe the clinical characteristics and outcome of a pregnant woman with COVID-19 and her neonate	The mother showed mild symptoms. Preterm spontaneous vaginal delivery at 32 occurred. RT-PCR test was negative for SARS-CoV-2 on neonatal throat swab.
Zeng L	Case series	Wuhan Children’s Hospital, Wuhan, Hubei, China	33 neonates born from 33 pregnant women with COVID-19	To evaluate the clinical outcome of neonates born to SARS-CoV-2-infected pregnant women and the possibility of vertical transmission	3 neonates out of 33 (9%) had positive swabs for SARS-CoV-2 and early symptoms (one might have been septic)
Zeng H	Case series	Zhongnan Hospital of Wuhan University, Wuhan, China	6 neonates born from 6 pregnant women with COVID-19	To evaluate the possibility of SARS-CoV-2 vertical transmission	Two neonates had positive IgM, 5 had positive IgG, all 6 had raised IL-6 levels. All neonates had negative RT-PCR tests for SARS-CoV-2 on a throat swab
Dong L	Case report	Renmin Hospital of Wuhan University, Wuhan, China	1 pregnant woman with COVID-19 1 neonate	To evaluate the possibility of SARS-CoV-2 vertical transmission	Neonate showed IgM and IgG positive, negative RT-PCR tests for SARS-CoV-2 on throat swab. Abnormal levels of the cytokine. Mother had Vaginal swab and breast milk RT-PCR tests for SARS-CoV-2 negative, NP swab positive.
Wang J	Case report	Wuhan Children’s Hospital, Tongji Medical College, Huazhong University of Science & Technology, Wuhan, China	1 pregnant woman with COVID-19 1 neonate	To describe the clinical outcome of a SARS-CoV-2-infected neonate with gastrointestinal onset	No exams were performed during the birth or immediately after. Ten days after the birth the mother was found positive. Neonate developed GI symptoms and had both NP and anal swab positive.
Wen R	Case report	Qingdao Women and Children’s Hospital, Qingdao, China	1 pregnant woman with COVID-19	To describe the clinical outcome of a SARS-CoV-2-infected pregnant woman	Pregnant women with SARS-CoV-2 and non-pregnant adults showed similar clinical characteristics.
Liu D	Case series	Union Hospital, Tongji Medical College, Huazhong University of Science and Technology, Wuhan, China	15 pregnant women with COVID-19 11 neonates	To describe the clinical characteristics and outcome of a pregnant woman with COVID-19 and her neonate	The severity of SARS-CoV-2 pneumonia was not affected by pregnancy and delivery in these patients.No stillbirth or abortion were reported. No neonate was infected and suffered asphyxia or death.
Fan C	Case series	Renmin Hospital of Wuhan University, Wuhan, China	2 pregnant women with COVID-19 2 neonates	To evaluate the possibility of SARS-CoV-2 vertical transmission	Real-time for SARS-CoV-2 on maternal serum, cord blood, placenta tissue, amniotic fluid, vaginal swab, breast milk, and newborn’s nasopharyngeal swab after delivery were negative. Both mothers and newborns had excellent outcomes.
Chen R	Case series	Renmin Hospital of Wuhan University, Wuhan, China	17 pregnant women with COVID-19 17 neonates	To compare epidural or general anesthesia for C-section delivery in pregnant women with COVID-19; to evaluate the clinical outcome of their newborns	Pregnant COVID-19 women safely received epidural or general anesthesia for Cesarean delivery All neonates tested negative for SARS-CoV-2.
Zhu H	Case series	5 hospitals in Hubei, China	9 pregnant women with COVID-19 10 neonates	To evaluate the clinical outcome of infected SARS-CoV-2 pregnant women and the possibility of vertical transmission	Perinatal COVID-19 infection could negatively affect neonates. The authors reported fetal distress, premature labor respiratory distress, thrombocytopenia and even one death. Real-time for SARS-CoV-2 on neonatal pharyngeal swabs showed negative results.
Chen H	Case series	Zhongnan Hospital of Wuhan University, Wuhan, China	9 pregnant women with COVID-19 9 neonates	To describe the clinical outcome of SARS-CoV-2-infected pregnant women and the possibility of vertical transmission	Pregnant women with SARS-CoV-2 pneumonia and non-pregnant adult patients SARS-CoV-2 pneumonia have similar clinical characteristics. The authors found no evidence of intrauterine infection (amniotic fluid, cord blood, neonatal throat swab, and breast milk all tested negative for SARS-CoV-2).
Liu Y	Case series	Hospitals outside of Wuhan (not better defined in the text), China	13 pregnant women with COVID-19 9 neonates1 stillborn	To evaluate the clinical outcome of SARS-CoV-2-infected pregnant women and the possibility of vertical transmission	Three Women discharged pregnant; 10 had C-section (emergency in 5 cases), 6 had preterm labor; 1 mother severe case (ARDS+MODS, needed ECMO treatment in ICU, outcome unknown). Neonates: 1 stillborn, 9 well. No evidence of vertical transmission (tests performed not specified).
Li Y	Case report	The First Affiliated Hospital, College of Medicine, Zhejiang University, Hangzhou, China.	1 pregnant woman with COVID-19 1 neonate	To evaluate the clinical outcome of SARS-CoV-2-infected pregnant woman and the possibility of vertical transmission	No evidence of vertical transmission (feces urine, umbilical cord blood amniotic fluid, placenta, and breast milk samples were negative for RT-PCR for SARS-CoV-2)
Wang X	Case report	Affiliated Infectious Hospital of Soochow University, Suzhou, China	1 pregnant woman with COVID-19 1 neonate	To evaluate the clinical outcome of SARS-CoV-2-infected pregnant woman and the possibility of vertical transmission	No evidence of vertical transmission. (samples of amniotic fluid, placenta, umbilical cord blood, throat swab, stool, gastric juice of the infant for SARS-CoV-2 RT-PCR tests were negative).
Chen S	Case series	Union Hospital, Tongji Medical College, Huazhong University of Science and Technology, Wuhan, China	3 pregnant women with COVID-19 3 neonates	To evaluate the placental features of SARS-CoV-2-infected pregnant women and the possibility of vertical transmission	Tissues from 3 placentas showed no morphological alterations due to SARS-CoV-2 infection: no evidence of vertical transmission (SARS-CoV-2 RT-PCR on neonatal swab were negative).
Zeng LK	Case report	Wuhan Children’s Hospital of Tongji Medical College, Huazhong University of Science & Technology, Wuhan, China.	1 neonate	To describe the clinical outcome of a SARS-CoV-2-infected neonate	The neonatal symptoms were mild (fever, rhinitis, refuse of feeding). Anal swab positive for SARS-CoV-2 longer than throat swab.
Ferrazzi E	Case series	Hospitals in Northern Italy, Italy	42 pregnant women with COVID-19 42 neonates	To describe the clinical characteristics and outcome of pregnant women with COVID-19 and their neonates	SARS-CoV-2 syndrome in pregnancy is often mild or moderate. Three SARS-CoV-2 positive newborns (two breastfed without a mask because of post-partum diagnosis and one positive test after vaginal operative delivery).
Iqbal SN	Case report	MedStar Washington Hospital Center, Washington, USA	1 pregnant woman with COVID-19 1 neonate	To evaluate the clinical outcome of a SARS-CoV-2-infected pregnant woman with vaginal delivery	Vaginal delivery in a SARS-CoV-2-infected mother was uncomplicated. No vertical transmission of SARS-CoV-2 or intrapartum transmission was found.
Song L	Case report	Wuhan Union Hospital, Wuhan, China	1 pregnant woman with COVID-19 1 neonate	To describe the perioperative management of a pregnant women with COVID-19 who underwent an urgent C-section	Both epidural and spinal and anesthesia during an emergent cesarean delivery were safe. SARS-CoV-2 RT-PCR on neonatal swab at 3 and 7 days of life were negative.

Abbreviations: COVID-19 (coronavirus disease 2019), SARS-CoV-2 (severe acute respiratory syndrome coronavirus 2), RT-PCR (reverse transcriptase-polymerase chain reaction), qRT-PCR (quantitative reverse transcriptase-polymerase chain reaction), CT scan (computed tomography scan), ICU (intensive care unit), CPR (cardiopulmonary resuscitation), ARDS (acute respiratory distress syndrome), MOF (multiple organ failure), C-section (cesarean section), IL-6 (interleukin 6), NP swab (nasopharyngeal swab), GI (gastro-intestinal), MODS (multiple organ dysfunction syndrome), ECMO (extracorporeal membrane oxygenation).

**Table 2:** Characteristics of the included pregnant women.

Maternal Characteristics	N	%
Total pregnant women	275	
Ongoing pregnancies	33	12
Abortion in the first trimester	3	1.1
**Gestational age**	**N**	**%**
Total pregnant women with data	208	76
Missing data	31	11
<37 weeks of gestation	48	23
≥37 weeks of gestation	160	77
**Type of delivery**	**N**	**%**
Deliveries	239	87
Vaginal	60	25
C-section *	179	75
Emergency C-section	27	11
**Symptoms**	**N**	**%**
Total pregnant women with data	269	98
Missing data	6	2
Fever	155	58
Cough	98	36
Mild respiratory symptoms (nasal congestion; sore throat)	9	3
Dyspnea	28	10
Myalgia/malaise/fatigue	37	14
GI symptoms (diarrhea or abdominal pain)	9	3
Asymptomatic	22	8
**Complications**	**N**	**%**
O2 therapy	36	13
ICU admission	10	4
Invasive ventilation/ECMO	5	2
Death	1	0.03
pPROM, preterm labor	24	9
**SARS-CoV-2 diagnosis**	**N**	**%**
RT-PCR for SARS-CoV-2 on Pharyngeal/ Nasopharyngeal Swab	260	95
IgM	6	2
IgG	9	3
**Laboratory findings**	**N**	**%**
Total pregnant women with data	108	39
Missing data	167	61
Lymphopenia (<1000/mmc)	31	29
Elevated CRP (>10 mg/L)	52	48
Elevated ALT (>45 U/L) or AST (>35 U/L)	9	8
**Chest CT**	**N**	**%**
Total pregnant women with data	171	62
Missing data (not reported/not done)	104	38
Positive	162	95
Negative	9	5
**Chest X-ray**	**N**	**%**
Total pregnant women with data	48	17
Missing data (not reported/not done)	227	83
Positive	48	100
Negative	0	0.0
**Symptoms Before Delivery and Age**	**Mean**	**SD**
Symptoms Before Delivery	31.06	3.61
Age	9.26	8.96

Abbreviations: SD (standard deviation), C-section (cesarean section), GI (gastro-intestinal), ICU (intensive care unit), ECMO (extracorporeal membrane oxygenation), pPROM (preterm premature rupture of membranes), RT-PCR (reverse transcriptase-polymerase chain reaction), SARS-CoV-2 (severe acute respiratory syndrome coronavirus 2), CRP (C-reactive protein), ALT (amino alanine transferase), AST (aspartate aminotransferase), CT scan (computed tomography scan). * total c-section including emergency c-section.

**Table 3:** Characteristics of neonates with positive SARS-Cov-2 RT-PCR.

Study	N	Symptoms Onset/ Diagnosis	Isolation *	Breast-Feeding *	GA	Birth-Weight	Sars-CoV-2 Positive Swab	Symptoms	Laboratory Test	Imaging	Length of Hospitalization/Symptoms
Paret M	1	25 days	/	/	39	Not reported	NP	Fever	Normal	Not reported	Not reported
Han MS	1	27 days	/	/	38+6	3730	NP	Fever, mild respiratory symptoms	Normal	X-ray Normal	18 days
Diaz CA	1	8 days	/	/	38+4	2500	NP	Distress	Normal (only CRP reported)	X-ray: ground glass opacities	Not reported
Kamali Aghdam M	1	15 days	/	/	Not reported	3460	NP	Fever, distress, need for noninvasive respiratory support	Normal	X-ray: normal	6
Wang J	1	19 days	/	/	38+6	3030	NP/Anal	Fever, gastrointestinal symptoms, cough	Thrombocytopenia	X-ray: positive, CT increased lungmarking	14
Zeng LK	1	17 days	/	/	39	Not reported	NP	Fever, gastrointestinal symptoms (vomit, diarrhea)	lymphocytosis	Cranial, abdomen CT: negative. Chest CT: positive. X-ray: positive	6
Zhang ZJ	1	17 Days	/	/	≥37	Not reported	Anal	Fever, cough, gastrointestinal symptoms (vomit)	Not reported	CT chest: increased lungmarking	23
	1	After birth (mother Sars-CoV-2 + during pregnancy)	Yes	No	40	Not reported	NP	Distress	Not reported	CT chest: increased lungmarking	Not reported
	1	After birth (mother Sars-CoV-2 + during pregnancy)	Yes	No	≥37	Not reported	Anal	Fever	Not reported	Not reported	30
	1	After birth (mother Sars-CoV-2 + during pregnancy)	Yes	No	40	Not reported	NP	No symptoms	Not reported	CT chest: Increased lung marking	16
Zeng L	1	After birth (mother Sars-CoV-2 + during pregnancy)	Yes	No	40	3250	NP	Fever	Normal	X-ray: pneumonia	2 (ICU)
	1	After birth (mother Sars-CoV-2 + during pregnancy)	Yes	No	40+4	3360	NP	Fever	leukocytosis, lymphocytopenia, and elevated creatine kinase–MB fraction	X-ray: pneumonia	4 (ICU)
	1	After birth (mother Sars-CoV-2 + during pregnancy)	Yes	No	32+2	1580	NP	Distress, refuse of feeding, need for mechanical ventilation	leukocytosis, thrombocytopenia, sepsis	X-ray: RDS and pneumonia	11 (ICU)
Ferrazzi E	1	Mothers’ COVID 19 diagnosis a few hours after delivery	No °	Yes	Not reported	Not reported	NP	Not reported	Not reported	Not reported	Not reported
	1	Mothers’ COVID 19 diagnosis a few hours after delivery	No °	Yes	Not reported	Not reported	NP	Not reported	Not reported	Not reported	Not reported
	1	After birth (mother Sars-CoV-2 + during pregnancy)	Yes	No	Not reported	Not reported	NP	Gastrointestinal symptoms, distress requiring one-day mechanical ventilation	Not reported	Not reported	ICU admission for prematurity/respiratory distress

Note: * applicable for neonates born from mothers with COVID-19 diagnosis during pregnancy; ° breastfed without surgical mask until mothers’ COVID-19 diagnosis. Abbreviations: GA (gestational age), SARS-CoV-2 (severe acute respiratory syndrome coronavirus 2), COVID-19 (coronavirus disease 2019), NP (nasopharyngeal), CRP (C-reactive protein), MB fraction (myocardial band fraction), CT scan (computed tomography scan), RDS (respiratory distress syndrome), NICU (neonatal intensive care unit).

**Table 4:** Characteristics of the included neonates.

Neonatal Characteristics		
Total neonates	248	
Stillborn neonates	2	
**Neonatal Characteristics**	**Mean**	**SD**
**Birthweight (g)**	2914	573
**Gestational age**	**N**	**%**
Neonates with data	196	79
<37 weeks	54	28
≥37 weeks	142	72
**Apgar score**	**N**	**%**
Neonates with data	190	77
<7 at 5’ *	5	3
>7 at 1’ and/or at 5’	185	97
>7 at 1’	145	76
(* 2 stillborn were included, the Apgar score was reported).		
Feeding *	**N**	**%**
Neonates with data	56	23
Breastfeeding	13	23
Formula	38	68
Both	5	9
**Isolation**	**N**	**%**
Neonates with data	171	70
Yes	125	73
No* (1 was isolated after 4 days, CA Diaz)	46	37
**Symptoms**	**N**	**%**
Neonates with data	160	65
**Fever**	13	8
SARS-CoV-2 +	9	70
**Mild respiratory symptoms (cough; rhinorrhea)**	4	3
SARS-CoV-2 +	3	75
**Distress**	21	13
SARS-CoV-2 +	5	24
**Gastrointestinal symptoms**	13	81
SARS-CoV-2 +	5	38
**Complications**	**N**	**%**
**Need for respiratory support**	8	5
SARS-CoV-2 +	3	38
**Other complications**	**N**	**%**
**Asphyxia**	2	13
Asphyxia in SARS-CoV-2 +	1	50
**Sepsis**	1	0.6
SARS-CoV-2 +	1	100
**Death or stillbirth**	3	1
**Stillbirth**	2	0.8
**Death**	1	0.4
**SARS-CoV-2 diagnosis**	**N**	**%**
**RT-PCR nasopharyngeal swab**		
Neonates with data	191	78
Positive *	14	7
* RT-PCR-positive on fecal samples only	2	1
**RT-PCR on fecal samples or anal swabs**	**N**	**%**
Neonates with data	28	15
Positive	5	18
**RT-PCR breast milk**	**N**	**%**
Neonates with data	25	10
Positive	0	0
**Other samples** (maternal vaginal swab, placenta tissue, cord blood or amniotic fluid)	**N**	**%**
Neonates with data	35	14
Positive	0	0
**Serological test**	N	%
Neonates with data	26	10
Negative	19	73
Positive IgG	4	15
Positive IgG+ IgM	3	12
**Laboratory findings**	**N**	**%**
Neonates with data	79	32
SARS-CoV-2 + with data	7	44
**Imaging**	**N**	**%**
Neonates with data	76	31
**Chest CT**	5	3
Chest CT in SARS-CoV-2+	4	80
Chest CT positive SARS-CoV-2 +	4	100
**Chest X-ray**	70	92
Chest X-ray in SARS-CoV-2 +	8	50
Chest X-ray positive in SARS-CoV-2 +	6	75
**Both X-ray and Chest CT**	2	3

Abbreviations: SD (standard deviation), SARS-CoV-2 (severe acute respiratory syndrome coronavirus 2), RT-PCR (reverse transcriptase-polymerase chain reaction), CT scan (computed tomography scan). * 2 stillborn were included, the Apgar score was reported.

## Supplementary Materials

The following are available online at https://www.mdpi.com/2076-0817/9/6/485/s1, File S1: PRISMA Checklist.
