# Supplementary material for: COVID-19 in Pregnant Women and Neonates: A Systematic Review of the Literature with Quality Assessment of the Studies
Source: Pathogens. 2020 Jun 18;9(6):485. doi: 10.3390/pathogens9060485 (PMC7350364; doi:10.3390/pathogens9060485)
Supplement: Supplementary file 1 [file pathogens-09-00485-s001.pdf]

pathogens

MDPI

Supplemental material: **PRISMA Checklist**

Section/topic	#	Checklist item	Reported on page #
**TITLE**			
Title	1	Identify the report as a systematic review, meta-analysis, or both.	1
**ABSTRACT**			
Structured summary	2	Provide a structured summary including, as applicable: background; objectives; data sources; study eligibility criteria, participants, and interventions; study appraisal and synthesis methods; results; limitations; conclusions and implications of key findings; systematic review registration number.	1
**INTRODUCTION**			
Rationale	3	Describe the rationale for the review in the context of what is already known.	1-2
Objectives	4	Provide an explicit statement of questions being addressed with reference to participants, interventions, comparisons, outcomes, and study design (PICOS).	1-2
**METHODS**			
Protocol and registration	5	Indicate if a review protocol exists, if and where it can be accessed (e.g., Web address), and, if available, provide registration information including registration number.	NA
Eligibility criteria	6	Specify study characteristics (e.g., PICOS, length of follow-up) and report characteristics (e.g., years considered, language, publication status) used as criteria for eligibility, giving rationale.	3
Information sources	7	Describe all information sources (e.g., databases with dates of coverage, contact with study authors to identify additional studies) in the search and date last searched.	2
Search	8	Present full electronic search strategy for at least one database, including any limits used, such that it could be repeated.	2
Study selection	9	State the process for selecting studies (i.e., screening, eligibility, included in systematic review, and, if applicable, included in the meta-analysis).	3
Data collection process	10	Describe method of data extraction from reports (e.g., piloted forms, independently, in duplicate) and any processes for obtaining and confirming data from investigators.	3
Data items	11	List and define all variables for which data were sought (e.g., PICOS, funding sources) and any assumptions and simplifications made.	3
Risk of bias in individual studies	12	Describe methods used for assessing risk of bias of individual studies (including specification of whether this was done at the study or outcome level), and how this information is to be used in any data synthesis.	3
Summary measures	13	State the principal summary measures (e.g., risk ratio, difference in means).	NA
Synthesis of results	14	Describe the methods of handling data and combining results of studies, if done, including measures of consistency (e.g., I^2) for each meta-analysis.	3
Section/topic	#	Checklist item	Reported on page #
Risk of bias across studies	15	Specify any assessment of risk of bias that may affect the cumulative evidence (e.g., publication bias, selective reporting within studies).	NA
Additional analyses	16	Describe methods of additional analyses (e.g., sensitivity or subgroup analyses, meta-regression), if done, indicating which were pre-specified.	NA
**RESULTS**			

Study selection	17	Give numbers of studies screened, assessed for eligibility, and included in the review, with reasons for exclusions at each stage, ideally with a flow diagram.	5
Study characteristics	18	For each study, present characteristics for which data were extracted (e.g., study size, PICOS, follow-up period) and provide the citations.	5-12
Risk of bias within studies	19	Present data on risk of bias of each study and, if available, any outcome level assessment (see item 12).	12-14
Results of individual studies	20	For all outcomes considered (benefits or harms), present, for each study: (a) simple summary data for each intervention group (b) effect estimates and confidence intervals, ideally with a forest plot.	14-23
Synthesis of results	21	Present results of each meta-analysis done, including confidence intervals and measures of consistency.	NA
Risk of bias across studies	22	Present results of any assessment of risk of bias across studies (see Item 15).	NA
Additional analysis	23	Give results of additional analyses, if done (e.g., sensitivity or subgroup analyses, meta-regression [see Item 16]).	NA
**DISCUSSION**			
Summary of evidence	24	Summarize the main findings including the strength of evidence for each main outcome; consider their relevance to key groups (e.g., healthcare providers, users, and policy makers).	23-25
Limitations	25	Discuss limitations at study and outcome level (e.g., risk of bias), and at review-level (e.g., incomplete retrieval of identified research, reporting bias).	25
Conclusions	26	Provide a general interpretation of the results in the context of other evidence, and implications for future research.	25
**FUNDING**			
Funding	27	Describe sources of funding for the systematic review and other support (e.g., supply of data); role of funders for the systematic review.	26

Supplemental material. Search strategy

1. Search strategy for EMBASE

**Search query**	('covid-19' OR 'severe acute respiratory syndrome coronavirus 2'/exp OR 'severe acute respiratory syndrome coronavirus 2' OR '2019-ncov' OR 'sars-cov-2' OR '2019ncov' OR '2019 coronavirus disease' OR ('wuhan' AND ('coronavirus'/exp OR 'coronavirus'))) AND [1-12-2019]/sd AND (child* OR pediatri* OR paediatri* OR infan* OR newborn* OR neonate* OR pregnan* OR breastfeed* OR fetal OR fetus OR obstetric* OR 'transplacental transmission' OR 'placental transmission' OR 'vertical transmission' OR intrauterine OR perinatal)
**Records without duplicates**	65
**Recors after title screening**	38 (27 excluded)
**Records after abstract screening**	9 (29 excluded)
**Records after full text screening**	1 (8 excluded)

**Exclusion criteria:**	23 No appropriate outcome	
	27 No appropriate population sample	
	13 No original article	
	1 No useful data	

**Records**	**Exclusion criteria**
Chen, Z., et al., Diagnosis and treatment recommendation for pediatric coronavirus disease-19. Zhejiang da xue xue bao. Yi xue ban = Journal of Zhejiang University. Medical sciences, 2020. 49(1): p. 1-8.	No original article
Çiftçi, E. and F. Çoksüer, Novel coronavirus infection: Covid-19. Flora, 2020. 25(1): p. 9-18.	No appropriate outcome
Ghosh, A., R. Gupta, and A. Misra, Telemedicine for diabetes care in India during COVID19 pandemic and national lockdown period: Guidelines for physicians. Diabetes and Metabolic Syndrome: Clinical Research and Reviews, 2020. 14(4): p. 273-276.	No appropriate population sample
Grant, W.B., et al., Evidence that vitamin d supplementation could reduce risk of influenza and covid-19 infections and deaths. Nutrients, 2020. 12(4).	No appropriate outcome
Hall, K.S., et al., Centring sexual and reproductive health and justice in the global COVID-19 response. The Lancet, 2020. 395(10231): p. 1175-1177.	No appropriate outcome
Haşlak, F., et al., Childhood Rheumatic Diseases and COVID-19 Pandemic: An Intriguing Linkage and a New Horizon. Balkan medical journal, 2020.	No appropriate population sample

Hedrich, C.M., COVID-19 – Considerations for the paediatric rheumatologist. Clinical Immunology, 2020. 214.	No original article
Huang, J.W., et al., Dialectical behavior therapy-based psychological intervention for woman in late pregnancy and early postpartum suffering from COVID-19: a case report. Journal of Zhejiang University: Science B, 2020.	No appropriate outcome
Hussein, J., COVID-19: What implications for sexual and reproductive health and rights globally? Sexual and Reproductive Health Matters, 2020. 28(1).	No appropriate outcome
Jones, V.G., et al., COVID-19 and Kawasaki Disease: Novel Virus and Novel Case. Hospital pediatrics, 2020.	No appropriate population sample
Kimberlin, D.W. and S. Stagno, Can SARS-CoV-2 Infection Be Acquired in Utero?: More Definitive Evidence Is Needed. JAMA - Journal of the American Medical Association, 2020: p. E1-E2.	No original article
Lenert, L. and B.Y. McSwain, Balancing Health Privacy, Health Information Exchange and Research in the Context of the COVID-19 Pandemic. Journal of the American Medical Informatics Association : JAMIA, 2020.	No appropriate outcome
Liew, M.F., et al., Preparing for CovID-19: Early experience from an intensive care unit in Singapore. Critical Care, 2020. 24(1).	No appropriate population sample
Parhar, K.K.S., et al., Optimizing provision of extracorporeal life support during the COVID-19 pande.mic: Practical considerations for Canadian jurisdictions. CMAJ, 2020. 192(14): p. E372-E374.	No appropriate outcome
Rajkumar, R.P., COVID-19 and mental health: A review of the existing literature. Asian Journal of Psychiatry, 2020. 52.	No original article

Sheng, W.H., et al., SARS-CoV-2 and COVID-19. Journal of Microbiology, Immunology and Infection, 2020.	No appropriate outcome
The, L., The gendered dimensions of COVID-19. The Lancet, 2020. 395(10231): p. 1168.	No appropriate outcome
Wei-ming, Y., et al., Expression of prothrombinase/fibroleukin gene fg12 in lung impairment in a murine severe acute respiratory syndrome model. Virologica Sinica, 2007. 22(3): p. 181-192.	No appropriate outcome
Xu, Y.H., et al., Clinical and computed tomographic imaging features of novel coronavirus pneumonia caused by SARS-CoV-2. Journal of Infection, 2020. 80(4): p. 394-400.	No appropriate population sample
Zhang, S., et al., The novel coronavirus (SARS-CoV-2) infections in China: prevention, control and challenges. Intensive Care Medicine, 2020. 46(4): p. 591-593.	No appropriate outcome
Zhao, W., et al., CT scans of patients with 2019 novel coronavirus (covid-19) pneumonia. Theranostics, 2020. 10(10): p. 4606-4613.	No appropriate population sample
Zhou, W.H., Perinatal and neonatal management plan for prevention and control of 2019 novel coronavirus infection (1st Edition). Chinese Journal of Contemporary Pediatrics, 2020. 22(2): p. 87-90.	No original article
Zhu, Y.L., B.B. Yang, and F. Wu, Understanding of COVID-19 in children from different perspectives of traditional Chinese medicine and western medicine. Chinese Traditional and Herbal Drugs, 2020. 51(4): p. 883-887.	No appropriate outcome
Perinatal and neonatal management plan for prevention and control of SARS-CoV-2 infection (2nd Edition). Zhongguo dang dai er ke za zhi = Chinese journal of contemporary pediatrics, 2020. 22(3): p. 195-198.	No original article

Alhazzani, W., et al., Surviving Sepsis Campaign: guidelines on the management of critically ill adults with Coronavirus Disease 2019 (COVID-19). Intensive care medicine, 2020.	No original article
Alonso DÌaz, C., et al., First case of neonatal infection due to SARS-CoV-2 in Spain. Anales de pediatria (Barcelona, Spain : 2003), 2020.	Included
Li, S., et al., The impact of covid-19 epidemic declaration on psychological consequences: A study on active weibo users. International Journal of Environmental Research and Public Health, 2020. 17(6).	No appropriate outcome
Li, Y., et al., Follow-up testing of viral nucleic acid in discharged patients with moderate type of 2019 coronavirus disease (COVID-19). Zhejiang da xue xue bao. Yi xue ban = Journal of Zhejiang University. Medical sciences, 2020. 49(1): p. 0.	No appropriate population sample
Pan, X., et al., Asymptomatic cases in a family cluster with SARS-CoV-2 infection. The Lancet Infectious Diseases, 2020. 20(4): p. 410-411.	No appropriate population sample
Pung, R., et al., Investigation of three clusters of COVID-19 in Singapore: implications for surveillance and response measures. The Lancet, 2020. 395(10229): p. 1039-1046.	No appropriate population sample
Shi, Y., et al., A quickly, effectively screening process of novel corona virus disease 2019 (COVID-19) in children in Shanghai, China. Annals of Translational Medicine, 2020. 8(5).	No appropriate population sample
Tian, S., et al., Characteristics of COVID-19 infection in Beijing. Journal of Infection, 2020. 80(4): p. 401-406.	No appropriate population sample
Wang, Y., et al., Oral Health Management of Children during the Epidemic Period of Coronavirus Disease 2019. Sichuan da xue xue bao. Yi xue ban = Journal of Sichuan University. Medical science edition, 2020. 51(2): p. 151-154.	No appropriate population sample

Wollenberg, A., et al., European Task Force on Atopic Dermatitis (ETFAD) statement on severe acute respiratory syndrome coronavirus 2 (SARS-Cov-2)-infection and atopic dermatitis. Journal of the European Academy of Dermatology and Venereology : JEADV, 2020.	No appropriate outcome
Yang, C., Does hand hygiene reduce SARS-CoV-2 transmission? Graefe's archive for clinical and experimental ophthalmology = Albrecht von Graefes Archiv fur klinische und experimentelle Ophthalmologie, 2020.	No appropriate population sample
Zehender, G., et al., GENOMIC CHARACTERISATION AND PHYLOGENETIC ANALYSIS OF SARS-COV-2 IN ITALY. Journal of medical virology, 2020.	No appropriate outcome
Erratum: Department of Error (The Lancet (2020) 395(10226) (809–815), (S0140673620303603), (10.1016/S0140-6736(20)30360-3)). The Lancet, 2020. 395(10229): p. 1038.	No original article
Chen, Z., et al., High-resolution computed tomography manifestations of COVID-19 infections in patients of different ages. European Journal of Radiology, 2020. 126.	No appropriate population sample
Gan, H., et al., Epidemiological analysis on 1 052 cases of COVID-19 in epidemic clusters. Zhonghua liu xing bing xue za zhi = Zhonghua liuxingbingxue zazhi, 2020. 41(5): p. E027.	No appropriate population sample
Huang, J., et al., Recommendation about the perioperative prevention of infection to healthcare workers and the anesthesia management of children with SARS-CoV-2 infection. World Journal of Pediatric Surgery, 2020. 3(1).	No appropriate population sample
Kang, X., et al., Anesthesia management in cesarean section for a patient with coronavirus disease 2019. Zhejiang da xue xue bao. Yi xue ban = Journal of Zhejiang University. Medical sciences, 2020. 49(1): p. 0.	No useful data
Kinner, S.A., et al., Prisons and custodial settings are part of a comprehensive response to COVID-19. The Lancet Public Health, 2020. 5(4): p. e188-e189.	No appropriate population sample

Sun, Q., et al., Lower mortality of COVID-19 by early recognition and intervention: experience from Jiangsu Province. Annals of Intensive Care, 2020. 10(1).	No appropriate population sample
Xie, C., et al., Comparison of different samples for 2019 novel coronavirus detection by nucleic acid amplification tests. International Journal of Infectious Diseases, 2020. 93: p. 264-267.	No appropriate population sample
Zhang, Y.H., et al., 2019 novel coronavirus infection in a three-month-old baby. Zhonghua er ke za zhi = Chinese journal of pediatrics, 2020. 58(3): p. 182-184.	No appropriate population sample
Zhang, Y., et al., Clinical and coagulation characteristics of 7 patients with critical COVID-2019 pneumonia and acro-ischemia. Zhonghua xue ye xue za zhi = Zhonghua xueyexue zazhi, 2020. 41: p. E006.	No appropriate population sample
Zhou, Y., et al., Clinical features and chest CT findings of coronavirus disease 2019 in infants and young children. Zhongguo dang dai er ke za zhi = Chinese journal of contemporary pediatrics, 2020. 22(3): p. 215-220.	No appropriate population sample
Liu K.-C., Xu P., Lv W.-F., Qiu X.-H., Yao J.-L., Gu J.-F., Wei W. CT manifestations of coronavirus disease-2019: A retrospective analysis of 73 cases by disease severity. European Journal of Radiology (2020) 126 Article Number: 108941. Date of Publication: 1 May 2020	No appropriate population sample
Lupia T., Scabini S., Pinna S.M., Di Perri G., De Rosa F.G., Corcione S. 2019-novel coronavirus outbreak: A new challenge. Journal of global antimicrobial resistance (2020). Date of Publication: 7 Mar 2020	No original article
Fan Q., Pan Y., Wu Q., Liu S., Song X., Xie Z., Liu Y., Zhao L., Wang Z., Zhang Y., Wu Z., Guan L., Lv X. Anal swab findings in an infant with COVID-19. Pediatric Investigation (2020) 4:1 (48-50). Date of Publication: 1 Mar 2020	No appropriate population sample
Xie Z. Pay attention to SARS-CoV-2 infection in children. Pediatric Investigation (2020) 4:1 (1-4). Date of Publication: 1 Mar 2020	No original article

Palacios Cruz M., Santos E., Velázquez Cervantes M.A., León Juárez M. COVID-19, a worldwide public health emergency. COVID-19, una emergencia de salud pública mundial.	No appropriate outcome
Wu M., Zhang Q., Yi D., Wu T., Chen H., Guo S., Li S., , Wang L., Zhao D., Hou Y., Wu G. Quantitative Proteomic Analysis Reveals Antiviral and Anti-inflammatory Effects of Puerarin in Piglets. Infected With Porcine Epidemic Diarrhea Virus. Frontiers in immunology (2020) 11 (169). Date of Publication: 2020	No appropriate outcome
Ko W.-C., Rolain J.-M., Lee N.-Y., Chen P.-L., Huang C.-T., Lee P.-I., Hsueh P.-R. Arguments in favor of remdesivir for treating SARS-CoV-2 infections. International journal of antimicrobial agents (2020) (105933). Date of Publication: 5 Mar 2020	No appropriate outcome
Chua M.S.Q., Lee J.C.S., Sulaiman S., Tan H.K. From the frontlines of COVID-19 - How prepared are we as obstetricians: a commentary. BJOG : an international journal of obstetrics and gynaecology (2020). Date of Publication: 4 Mar 2020	No original article
Yang P., Liu P., Li D., Zhao D. Corona Virus Disease 2019, a growing threat to children? The Journal of infection (2020). Date of Publication: 3 Mar 2020	No original article
Zhang J., Zhou L., Yang Y., Peng W., Wang W., Chen X. Therapeutic and triage strategies for 2019 novel coronavirus disease in fever clinics. The Lancet Respiratory Medicine (2020) 8:3 (e11-e12). Date of Publication: 1 Mar 2020	No appropriate population sample
Xu X., Chen P., Wang J., Feng J., Zhou H., Li X., Zhong W., Hao P. Evolution of the novel coronavirus from the ongoing Wuhan outbreak and modeling of its spike protein for risk of human transmission. Science China. Life sciences (2020) 63:3 (457-460). Date of Publication: 1 Mar 2020	No appropriate outcome
Calda P., Břešťák M., Fischerová D., Zikán M., Smetanová D., Machala L. Coronavirus infection and pregnancy (Covid-19) Koronavirová infekce a těhotenství (Covid-19). Aktualni Gynekologie a Porodnictvi (2020) 12 (17-19). Date of Publication: 2020	No original article
Chen W.-H., Strych U., Hotez P.J., Bottazzi M.E. The SARS-CoV-2 Vaccine Pipeline: an Overview. Current Tropical Medicine Reports (2020). Date of Publication: 2020	No appropriate outcome

Gilbert M., Pullano G., Pinotti F., Valdano E., Poletto C., Boëlle P.-Y., D'Ortenzio E., Yazdanpanah Y., Eholie S.P., Altmann M., Gutierrez B., Kraemer M.U.G., Colizza V. Preparedness and vulnerability of African countries against importations of COVID-19: a modelling study. The Lancet (2020). Date of Publication: 2020	No appropriate outcome
Sun K., Chen J., Viboud C. Early epidemiological analysis of the coronavirus disease 2019 outbreak based on crowdsourced data: a population-level observational study. The Lancet Digital Health (2020). Date of Publication: 2020	No appropriate outcome
Mizumoto K., Chowell G. Transmission potential of the novel coronavirus (COVID-19) onboard the diamond Princess Cruises Ship, 2020. Infectious Disease Modelling (2020) 5 (264-270). Date of Publication: 1 Jan 2020	No appropriate population sample
Rodriguez-Morales A.J., Gallego V., Escalera-Antezana J.P., Méndez C.A., Zambrano L.I., Franco-Paredes C., Suárez J.A., Rodriguez-Enciso H.D., Balbin-Ramon G.J., Savio-Larriera E., Risquez A., Cimerman S. COVID-19 in Latin America: The implications of the first confirmed case in Brazil. Travel Medicine and Infectious Disease (2020) Article Number: 101613. Date of Publication: 2020	No appropriate population sample
Qian S., Zhang W., Jia X., Sun Z., Zhang Y., Xiao Y., Li Z. Isolation and Identification of Porcine Epidemic Diarrhea Virus and Its Effect on Host Natural Immune Response. Frontiers in Microbiology (2019) 10 Article Number: 2272. Date of Publication: 4 Oct 2019	No appropriate outcome

2. Search strategy for MEDLINE

**Search query**	("COVID-19"[All Fields] OR "severe acute respiratory syndrome coronavirus 2"[Supplementary Concept] OR "severe acute respiratory syndrome coronavirus 2"[All Fields] OR "2019-nCoV"[All Fields] OR "SARS-CoV-2"[All Fields] OR "2019nCoV"[All Fields] OR “2019 coronavirus disease” OR (("Wuhan"[All Fields] AND ("coronavirus"[MeSH Terms] OR "coronavirus"[All Fields])) AND 2019/12[PDAT] : 2030[PDAT])) AND (child* OR pediatri* OR paediatri* OR infan* OR newborn* OR neonate* OR pregnan* OR breastfeed* OR fetal OR fetus OR obstetric* OR "transplacental transmission" OR "placental transmission" OR "vertical transmission" OR intrauterine OR perinatal)
**Records without duplicates**	635
**Title screening**	342 (293 excluded)

**Abstract screening**	74 (268 excluded)
**Full text screening**	33 (41 excluded)
**Exclusion criteria**	229 No appropriate outcome
	227 No appropriate population sample
	133 No original article
	4 No useful data
	9 Overlapping data

**Records**	**Exclusion criteria**
1: Dotters-Katz SK, Hughes BL. Considerations for Obstetric Care during theCOVID-19 Pandemic. Am J Perinatol. 2020 Apr 17. doi: 10.1055/s-0040-1710051.[Epub ahead of print] PubMed PMID: 32303077.	No original article
2: Weiner HL, Adelson PD, Brockmeyer DL, Maher CO, Gupta N, Smyth MD, Jea A,Blount JP, Riva-Cambrin J, Lam SK, Ahn ES, Albert GW, Leonard JR. Editorial.Pediatric neurosurgery along with Children's Hospitals' innovations are rapid anduniform in response to the COVID-19 pandemic. J Neurosurg Pediatr. 2020 Apr17:1-3. doi: 10.3171/2020.4.PEDS20240. [Epub ahead of print] PubMed PMID:32302988	No appropriate population sample
3: Arlt W, Dekkers OM, Leger J, Semple RK. Endocrinology in the time of COVID-19.Eur J Endocrinol. 2020 Apr 1. pii: EJE-20-0386. doi: 10.1530/EJE-20-0386. [Epubahead of print] Review. PubMed PMID: 32302976.	No appropriate population sample
4: Yang P, Wang X, Liu P, Wei C, He B, Zheng J, Zhao D. Clinical characteristics and risk assessment of newborns born to mothers with COVID-19. J Clin Virol. 2020Apr 10;127:104356. doi: 10.1016/j.jcv.2020.104356.	Included

[Epub ahead of print] PubMedPMID: 32302955.	
5: Tan YP, Tan BY, Pan J, Wu J, Zeng SZ, Wei HY. Epidemiologic and clinicalcharacteristics of 10 children with coronavirus disease 2019 in Changsha, China. J Clin Virol. 2020 Apr 10;127:104353. doi: 10.1016/j.jcv.2020.104353. [Epub aheadof print] PubMed PMID: 32302953.	No appropriate population sample
6: Yuan R, Xu QH, Xia CC, Lou CY, Xie Z, Ge QM, Shao Y. Psychological status ofparents of hospitalized children during the COVID-19 epidemic in China.Psychiatry Res. 2020 Apr 13;288:112953. doi: 10.1016/j.psychres.2020.112953.[Epub ahead of print] PubMed PMID: 32302814.	No appropriate outcome
7: Wang X, Liu W, Zhao J, Lu Y, Wang X, Yu C, Hu S, Shen N, Liu W, Sun Z, Li W.Clinical characteristics of 80 hospitalized frontline medical workers infectedwith COVID-19 in Wuhan, China. J Hosp Infect. 2020 Apr 14. pii:S0195-6701(20)30194-8. doi: 10.1016/j.jhin.2020.04.019. [Epub ahead of print]PubMed PMID: 32302722.	No appropriate population sample
8: Li Y, Zhou W, Yang L, You R. Physiological and pathological regulation ofACE2, the SARS-CoV-2 receptor. Pharmacol Res. 2020 Apr 14:104833. doi:10.1016/j.phrs.2020.104833. [Epub ahead of print] Review. PubMed PMID: 32302706.	No appropriate outcome
9: Stephens EH, Dearani JA, Guleserian KJ, Overman DM, Tweddell JS, Backer CL,Romano JC, Bacha E. COVID-19: Crisis Management in Congenital Heart Surgery. Ann Thorac Surg. 2020 Apr 11. pii: S0003-4975(20)30540-3. doi:10.1016/j.athoracsur.2020.04.001. [Epub ahead of print] PubMed PMID: 32302660.	No appropriate population sample

10: Lee J. Mental health effects of school closures during COVID-19. Lancet ChildAdolesc Health. 2020 Apr 14. pii: S2352-4642(20)30109-7. doi:10.1016/S2352-4642(20)30109-7. [Epub ahead of print] PubMed PMID: 32302537.	No appropriate outcome
11: Garg SK, Rodbard D, Hirsch IB, Forlenza GP. Managing New-Onset Type 1Diabetes During the COVID-19 Pandemic: Challenges and Opportunities. DiabetesTechnol Ther. 2020 Apr 17. doi: 10.1089/dia.2020.0161. [Epub ahead of print]PubMed PMID: 32302499.	No appropriate population sample
12: Vlachodimitropoulou Koumoutsea E, Vivanti AJ, Shehata N, Benachi A, Le Gouez A, Desconclois C, Whittle W, Snelgrove J, Malinowski KA. COVID19 and acutecoagulopathy in pregnancy. J Thromb Haemost. 2020 Apr 17. doi: 10.1111/jth.14856.[Epub ahead of print] PubMed PMID: 32302459.	No appropriate outcome
13: Akima S, McLintock C, Hunt BJ. RE: ISTH interim guidance to recognition andmanagement of coagulopathy in COVID-19. J Thromb Haemost. 2020 Apr 17. doi:10.1111/jth.14853. [Epub ahead of print] PubMed PMID: 32302442.	No appropriate outcome
14: Damle B, Vourvahis M, Wang E, Leaney J, Corrigan B. Clinical PharmacologyPerspectives on the Antiviral Activity of Azithromycin and Use in COVID-19. Clin Pharmacol Ther. 2020 Apr 17. doi: 10.1002/cpt.1857. [Epub ahead of print] Review.PubMed PMID: 32302411.	No appropriate outcome
15: Barnes BJ, Adrover JM, Baxter-Stoltzfus A, Borczuk A, Cools-Lartigue J,Crawford JM, Daßler-Plenker J, Guerci P, Huynh C, Knight JS, Loda M, Looney MR,McAllister F, Rayes R, Renaud S, Rousseau S, Salvatore S, Schwartz RE, Spicer JD,Yost CC, Weber A, Zuo Y, Egeblad M. Targeting potential drivers of COVID-19:Neutrophil extracellular traps. J Exp Med. 2020 Jun 1;217(6). pii: e20200652.doi: 10.1084/jem.20200652. PubMed PMID: 32302401.	No appropriate outcome

16: Patel PD, Cobb J, Wright D, Turer R, Jordan T, Humphrey A, Kepner AL, SmithG, Rosenbloom ST. Rapid Development of Telehealth Capabilities within PediatricPatient Portal Infrastructure for COVID-19 Care: Barriers, Solutions, Results. J Am Med Inform Assoc. 2020 Apr 17. pii: ocaa065. doi: 10.1093/jamia/ocaa065. [Epub	No appropriate outcome
17: Lagana SM, De Michele S, Lee MJ, Emond JC, Griesemer AD, Tulin-Silver SA,Verna EC, Martinez M, Lefkowitch JH. COVID-19 Associated Hepatitis ComplicatingRecent Living Donor Liver Transplantation. Arch Pathol Lab Med. 2020 Apr 17. doi:10.5858/arpa.2020-0186-SA. [Epub ahead of print] PubMed PMID: 32302212.	No appropriate population sample
18: Li L, Liu D, Yang L. Reply to "CT Findings of Pregnant Women With CoronavirusDisease (COVID-19) Pneumonia". AJR Am J Roentgenol. 2020 Apr 17:W1. doi:10.2214/AJR.20.23248. [Epub ahead of print] PubMed PMID: 32302206.	No original article
19: Chen L, Li Q, Zheng D, Jiang H, Wei Y, Zou L, Feng L, Xiong G, Sun G, Wang H,Zhao Y, Qiao J. Clinical Characteristics of Pregnant Women with Covid-19 inWuhan, China. N Engl J Med. 2020 Apr 17. doi: 10.1056/NEJMc2009226. [Epub aheadof print] PubMed PMID: 32302077.	Overlapping data
20: Chen X, Zhao B, Qu Y, Chen Y, Xiong J, Feng Y, Men D, Huang Q, Liu Y, Yang B,Ding J, Li F. Detectable serum SARS-CoV-2 viral load (RNAaemia) is closelycorrelated with drastically elevated interleukin 6 (IL-6) level in critically illCOVID-19 patients. Clin Infect Dis. 2020 Apr 17. pii: ciaa449. doi:10.1093/cid/ciaa449. [Epub ahead of print] PubMed PMID: 32301997.	No appropriate outcome
21: Paret M, Lighter J, Pellett Madan R, Raabe VN, Shust GF, Ratner AJ.SARS-CoV-2 infection (COVID-19) in febrile infants without respiratory distress. Clin Infect Dis. 2020 Apr 17. pii: ciaa452. doi:	Included

10.1093/cid/ciaa452. [Epub aheadof print] PubMed PMID: 32301967.	
22: Franco-Paredes C. Albert Camus' 'The COVID-19 Plague' Revisited. Clin Infect Dis. 2020 Apr 17. pii: ciaa454. doi: 10.1093/cid/ciaa454. [Epub ahead of print]PubMed PMID: 32301966.	No appropriate population sample
23: Li W, Zhang B, Lu J, Liu S, Chang Z, Cao P, Liu X, Zhang P, Ling Y, Tao K,Chen J. The characteristics of household transmission of COVID-19. Clin InfectDis. 2020 Apr 17. pii: ciaa450. doi: 10.1093/cid/ciaa450. [Epub ahead of print]PubMed PMID: 32301964.	No appropriate population sample
24: Ademuyiwa AO, Bekele A, Berhea AB, Borgstein E, Capo-Chichi N, Derbew M,Evans FM, Feyssa MD, Galukande M, Gawande AA, Gueye SM, Harrison E, Jani P,Kaseje N, Litswa L, Mammo TN, Mellin-Olsen J, Muguti G, Nabukenya MT, Ngoga E,Ntirenganya F, Rulisa S, Starr N, Tabiri S, Tadesse M, Walker I, Weiser TG, Wren SM. COVID-19 Preparedness within the Surgical, Obstetric and Anesthetic Ecosystemin Sub Saharan Africa. Ann Surg. 2020 Apr 13. doi: 10.1097/SLA.0000000000003964. [Epub ahead of print] PubMed PMID: 32301806.	No appropriate outcome
25: Grünebaum A, Dudenhausen J, McCullough LB, Chervenak FA. Women and childrenfirst: the need for ringfencing during the COVID-19 pandemic. J Perinat Med. 2020Apr 16. pii: /j/jpme.ahead-of-print/jpm-2020-0149/jpm-2020-0149.xml. doi:10.1515/jpm-2020-0149. [Epub ahead of print] PubMed PMID: 32301752.	No original article
26: Jiang S, Liu P, Xiong G, Yang Z, Wang M, Li Y, Yu XJ. Coinfection ofSARS-CoV-2 and multiple respiratory pathogens in children. Clin Chem Lab Med.2020 Apr 16. pii: /j/cclm.ahead-of-print/cclm-2020-0434/cclm-2020-0434.xml. doi: 10.1515/cclm-2020-0434.	No appropriate population sample

[Epub ahead of print] PubMed PMID: 32301747.	
27: Monteleone PA, Nakano M, Lazar V, Gomes AP, de H Martin, Bonetti TC. A reviewc of initial data on pregnancy during the COVID-19 outbreak: implications forvassisted reproductive treatments. JBRA Assist Reprod. 2020 May 1;24(2):219-225.doi: 10.5935/1518-0557.20200030. Review. PubMed PMID: 32301600.	No original article
28: Du W, Yu J, Wang H, Zhang X, Zhang S, Li Q, Zhang Z. Clinical characteristicsof COVID-19 in children compared with adults in Shandong Province, China.Infection. 2020 Apr 16. doi: 10.1007/s15010-020-01427-2. [Epub ahead of print]PubMed PMID: 32301099.	No appropriate population sample
29: Ding J, Fu H, Liu Y, Gao J, Li Z, Zhao X, Zheng J, Sun W, Ni H, Ma X, Feng J,Wu A, Liu J, Wang Y, Geng P, Chen Y. Prevention and control measures in radiologydepartment for COVID-19. Eur Radiol. 2020 Apr 16. doi:10.1007/s00330-020-06850-5. [Epub ahead of print] PubMed PMID: 32300968.	No appropriate outcome
30: Promislow DEL. A geroscience perspective on COVID-19 mortality. J Gerontol A Biol Sci Med Sci. 2020 Apr 17. pii: glaa094. doi: 10.1093/gerona/glaa094. [Epubahead of print] PubMed PMID: 32300796.	No appropriate outcome
31: Ramphul K, Mejias SG. Coronavirus Disease: A Review of a New Threat to PublicHealth. Cureus. 2020 Mar 15;12(3):e7276. doi: 10.7759/cureus.7276. Review. PubMedPMID: 32300496; PubMed Central PMCID: PMC7158591.	No original article

32: Ascierto PA, Fox B, Urba W, Anderson AC, Atkins MB, Borden EC, Brahmer J,Butterfield LH, Cesano A, Chen D, de Gruijl T, Dillman RO, Drake CG, Emens LA,Gajewski TF, Gulley JL, Stephen Hodi F, Hwu P, Kaufman D, Kaufman H, Lotze M,McNeel DG, Margolin K, Marincola F, Mastrangelo MJ, Maus MV, Parkinson DR, RomeroPJ, Sondel PM, Spranger S, Sznol M, Weiner GJ, Wiggington JM, Weber JS. Insights from immuno-oncology: the Society for Immunotherapy of Cancer Statement on accessto IL-6-targeting therapies for COVID-19. J Immunother Cancer. 2020 Apr;8(1).pii: e000878. doi: 10.1136/jitc-2020-000878. PubMed PMID: 32300051.	No appropriate outcome
33: Gupta L, Misra DP, Agarwal V, Balan S, Agarwal V. Management of rheumaticdiseases in the time of covid-19 pandemic: perspectives of rheumatologypractitioners from India. Ann Rheum Dis. 2020 Apr 16. pii:annrheumdis-2020-217509. doi: 10.1136/annrheumdis-2020-217509. [Epub ahead ofprint] PubMed PMID: 32299795.	No appropriate population sample
34: Paquin V, Lemire M, King S. Ecosystem approaches to the risk forschizophrenia. Schizophr Res. 2020 Apr 10. pii: S0920-9964(20)30168-7. doi:10.1016/j.schres.2020.03.057. [Epub ahead of print] PubMed PMID: 32299718.	No appropriate population sample
35: Mogharab V, Pasha AMK, Javdani F, Hatami N. The first case of COVID-19infection in a 75-day-old infant in Jahrom City, south of Iran. J Formos MedAssoc. 2020 Apr 13. pii: S0929-6646(20)30107-8. doi: 10.1016/j.jfma.2020.03.015. [Epub ahead of print] PubMed PMID: 32299654.	No appropriate population sample
36: Chirico F, Nucera G, Magnavita N. COVID-19: Protecting Healthcare Workers is a priority. Infect Control Hosp Epidemiol. 2020 Apr 17:1-4. doi:10.1017/ice.2020.148. [Epub ahead of print] PubMed PMID: 32299519.	No appropriate population sample

37: Zhao ZY, Chen Q, Zhao B, Hannah MN, Wang N, Wang YX, Xuan XF, Rui J, Chu MJ, Yu SS, Wang Y, Liu XC, An R, Pan LL, Chiang YC, Su YH, Zhao BH, Chen TM. Relativetransmissibility of shigellosis among male and female individuals: a modelingstudy in Hubei Province, China. Infect Dis Poverty. 2020 Apr 17;9(1):39. doi:10.1186/s40249-020-00654-x. PubMed PMID: 32299485.	No appropriate outcome
38: Bulut C, Kato Y. Epidemiology of COVID-19. Turk J Med Sci. 2020 Apr 17. doi: 10.3906/sag-2004-172. [Epub ahead of print] PubMed PMID: 32299206.	No appropriate outcome
39: Bachanova V, Bishop MR, Dahi P, Dholaria B, Grupp SA, Hayes-Lattin B,Janakiram M, Maziarz RT, McGuirk JP, Nastoupil LJ, Oluwole OO, Perales MA, PorterDL, Riedell PA. CAR T Cell Therapy During the COVID-19 Pandemic. Biol BloodMarrow Transplant. 2020 Apr 13. pii: S1083-8791(20)30217-2. doi:10.1016/j.bbmt.2020.04.008. [Epub ahead of print] Review. PubMed PMID: 32298807.	No appropriate outcome
40: Procianoy RS, Silveira RC, Manzoni P, Sant'Anna G. Neonatal COVID-19: little evidence and the need for more information. J Pediatr (Rio J). 2020 Apr 11. pii: S0021-7557(20)30143-1. doi: 10.1016/j.jped.2020.04.002. [Epub ahead of print]PubMed PMID: 32298645.	No original article
41: de Niet A, Waanders BL, Walraven I. The role of children in the transmission of mild SARS-CoV-2 infection. Acta Paediatr. 2020 Apr 16. doi: 10.1111/apa.15310.[Epub ahead of print] PubMed PMID: 32298494.	No appropriate outcome
42: Frieden IJ, Püttgen KB, Drolet BA, Garzon MC, Chamlin SL, Pope E, Mancini AJ,Lauren CT, Mathes EF, Siegel DH, Gupta D, Haggstrom AN, Tollefson MM, Baselga E, Morel KD, Shah SD, Holland KE, Adams DM, Horii KA, Newell BD, Powell J, McCuaigCC, Nopper AJ, Metry DW, Maguiness S;	No appropriate population sample

Hemangioma Investigator Group. Managementof Infantile Hemangiomas during the COVID Pandemic. Pediatr Dermatol. 2020 Apr16. doi: 10.1111/pde.14196. [Epub ahead of print] PubMed PMID: 32298480.	
43: Fix OK, Hameed B, Fontana RJ, Kwok RM, McGuire BM, Mulligan DC, Pratt DS,Russo MW, Schilsky ML, Verna EC, Loomba R, Cohen DE, Bezerra JA, Reddy KR, Chung RT. Clinical Best Practice Advice for Hepatology and Liver Transplant ProvidersDuring the COVID-19 Pandemic: AASLD Expert Panel Consensus Statement. Hepatology.2020 Apr 16. doi: 10.1002/hep.31281. [Epub ahead of print] PubMed PMID: 32298473.	No appropriate population sample
44: Sainati L, Biffi A. How we deal with the COVID-19 epidemic in an Italianpediatric onco-hematology clinic located in a region at high density of cases. BrJ Haematol. 2020 Apr 16. doi: 10.1111/bjh.16699. [Epub ahead of print] PubMedPMID: 32298461.	No appropriate population sample
45: Li M, Chen L, Zhang J, Xiong C, Li X. The SARS-CoV-2 receptor ACE2 expressionof maternal-fetal interface and fetal organs by single-cell transcriptome study. PLoS One. 2020 Apr 16;15(4):e0230295. doi: 10.1371/journal.pone.0230295.eCollection 2020. PubMed PMID: 32298273.	No appropriate outcome
46: Garg S, Kim L, Whitaker M, O'Halloran A, Cummings C, Holstein R, Prill M,Chai SJ, Kirley PD, Alden NB, Kawasaki B, Yousey-Hindes K, Niccolai L, AndersonEJ, Openo KP, Weigel A, Monroe ML, Ryan P, Henderson J, Kim S, Como-Sabetti K,Lynfield R, Sosin D, Torres S, Muse A, Bennett NM, Billing L, Sutton M, West N,Schaffner W, Talbot HK, Aquino C, George A, Budd A, Brammer L, Langley G, HallAJ, Fry A. Hospitalization Rates and Characteristics of Patients Hospitalizedwith Laboratory-Confirmed Coronavirus Disease 2019 - COVID-NET, 14 States, March 1-30, 2020. MMWR Morb Mortal Wkly Rep. 2020 Apr 17;69(15):458-464. doi:10.15585/mmwr.mm6915e3. PubMed PMID: 32298251.	No useful data

47: Ghinai I, Woods S, Ritger KA, McPherson TD, Black SR, Sparrow L, FricchioneMJ, Kerins JL, Pacilli M, Ruestow PS, Arwady MA, Beavers SF, Payne DC, KirkingHL, Layden JE. Community Transmission of SARS-CoV-2 at Two Family Gatherings - Chicago, Illinois, February-March 2020. MMWR Morb Mortal Wkly Rep. 2020 Apr17;69(15):446-450. doi: 10.15585/mmwr.mm6915e1. PubMed PMID: 32298246.	No appropriate population sample
48: Chen WH, Hotez PJ, Bottazzi ME. Potential for developing a SARS-CoVreceptor-binding domain (RBD) recombinant protein as a heterologous human vaccineagainst coronavirus infectious disease (COVID)-19. Hum Vaccin Immunother. 2020Apr 16:1-4. doi: 10.1080/21645515.2020.1740560. [Epub ahead of print] PubMedPMID: 32298218.	No appropriate outcome
49: Li L, Liu D, Yang L. Follow-Up Information About the Four Pregnant PatientsWith Coronavirus Disease (COVID-19) Pneumonia Who Were Still in the Hospital atthe End of Our Study. AJR Am J Roentgenol. 2020 Apr 16:W1-W2. doi:10.2214/AJR.20.23247. [Epub ahead of print] PubMed PMID: 32298148.	Included
50: Han MS, Seong MW, Heo EY, Park JH, Kim N, Shin S, Cho SI, Park SS, Choi EH.Sequential analysis of viral load in a neonate and her mother infected withSARS-CoV-2. Clin Infect Dis. 2020 Apr 16. pii: ciaa447. doi: 10.1093/cid/ciaa447.[Epub ahead of print] PubMed PMID: 32297925.	Included
51: Scorzolini L, Corpolongo A, Castilletti C, Lalle E, Mariano A, Nicastri E.Comment of the potential risks of sexual and vertical transmission of Covid-19infection. Clin Infect Dis. 2020 Apr 16. pii: ciaa445. doi: 10.1093/cid/ciaa445. [Epub ahead of print] PubMed PMID: 32297915.	No original article

52: Rezaei N. COVID-19 affects Healthy Pediatricians more than PediatricPatients. Infect Control Hosp Epidemiol. 2020 Apr 16:1-3. doi:10.1017/ice.2020.139. [Epub ahead of print] PubMed PMID: 32297847.	No appropriate population sample
53: Siskind D, Honer WG, Clark S, Correll CU, Hasan A, Howes O, Kane JM, KellyDL, Laitman R, Lee J, MacCabe JH, Myles N, Nielsen J, Schulte PF, Taylor D,Verdoux H, Wheeler A, Freudenreich O. Consensus statement on the use of clozapineduring the COVID-19 pandemic. J Psychiatry Neurosci. 2020 Apr 3;45(4):200061.doi: 10.1503/jpn.200061. [Epub ahead of print] PubMed PMID: 32297722.	No appropriate outcome
54: Lam JCM, Moshi GB, Ang SH, Chew HM, Ng QH, Madjukie A, M L. Management ofCOVID-19 Related Paediatric Blood Samples in a Clinical Haematology Laboratory.Br J Haematol. 2020 Apr 16. doi: 10.1111/bjh.16721. [Epub ahead of print] PubMed PMID: 32297317.	No appropriate outcome
55: Costi S, Caporali R, Cimaz R. Dealing with COVID-19 in a PediatricRheumatology Unit in Italy. Paediatr Drugs. 2020 Apr 16. doi:10.1007/s40272-020-00395-2. [Epub ahead of print] PubMed PMID: 32297225.	No appropriate outcome
56: Hermann A, Deligiannidis KM, Bergink V, Monk C, Fitelson EM, Robakis TK,Birndorf C. Response to SARS-Covid-19-related visitor restrictions on labor anddelivery wards in New York City. Arch Womens Ment Health. 2020 Apr 15. doi:10.1007/s00737-020-01030-2. [Epub ahead of print] PubMed PMID: 32296947.	No appropriate outcome
57: Xu P, Zhou Q, Xu J. Mechanism of thrombocytopenia in COVID-19 patients. AnnHematol. 2020 Apr 15. doi: 10.1007/s00277-020-04019-0. [Epub ahead of print]Review. PubMed PMID: 32296910.	No appropriate outcome

58: LaCourse SM, John-Stewart G, Adams Waldorf KM. Importance of inclusion of pregnant and breastfeeding women in COVID-19 therapeutic trials. Clin Infect Dis.2020 Apr 15. pii: ciaa444. doi: 10.1093/cid/ciaa444. [Epub ahead of print] PubMedPMID: 32296817.	No appropriate outcome
59: Chen F, Hao YT, Zhang ZJ, Tang JL, Xia JL, Zhan SY, Zhao Y, Du ZC, Wei YY,Shen SP, Jiang QW, Li LM. [An urgent call for raising the scientific rigorousnessof clinical trials on COVID-19]. Zhonghua Liu Xing Bing Xue Za Zhi. 2020 Mar10;41(3):301-302. doi: 10.3760/cma.j.issn.0254-6450.2020.03.004. Chinese. PubMed PMID: 32294824.	No appropriate outcome
60: Warrillow S, Austin D, Cheung W, Close E, Holley A, Horgan B, Jansen M, JoyntG, Lister P, Moodie S, Nichol A, Nicholls M, Peake S, Skowronski G, Streat S,White B, Willmott L. ANZICS guiding principles for complex decision making duringthe COVID-19 pandemic. Crit Care Resusc. 2020 Apr 15. [Epub ahead of print]PubMed PMID: 32294810.	No original article
61: Yang H, Sun G, Tang F, Peng M, Gao Y, Peng J, Xie H, Zhao Y, Jin Z. Clinical Features and Outcomes of Pregnant Women Suspected of Coronavirus Disease 2019. J Infect. 2020 Apr 12. pii: S0163-4453(20)30212-7. doi: 10.1016/j.jinf.2020.04.003.[Epub ahead of print] PubMed PMID: 32294503; PubMed Central PMCID: PMC7152867.	Included
62: Bovbjerg ML. Current Resources for Evidence-Based Practice, May 2020. JObstet Gynecol Neonatal Nurs. 2020 Apr 10. pii: S0884-2175(20)30044-7. doi:10.1016/j.jogn.2020.04.001. [Epub ahead of print] PubMed PMID: 32294453; PubMedCentral PMCID: PMC7151442.	No appropriate outcome
63: Lowe B, Bopp B. COVID-19 vaginal delivery - a case report. Aust N Z J Obstet Gynaecol. 2020 Apr 15. doi: 10.1111/ajo.13173. [Epub ahead of print] PubMed PMID:32294229	Included

64: Yuan S, Liao Z, Huang H, Jiang B, Zhang X, Wang Y, Zhao M. Comparison of the Indicators of Psychological Stress in the Population of Hubei Province andNon-Endemic Provinces in China During Two Weeks During the Coronavirus Disease2019 (COVID-19) Outbreak in February 2020. Med Sci Monit. 2020 Apr 15;26:e923767.doi: 10.12659/MSM.923767. PubMed PMID: 32294078.	No appropriate outcome
65: Henderson LA, Canna SW, Schulert GS, Volpi S, Lee PY, Kernan KF, Caricchio R,Mahmud S, Hazen MM, Halyabar O, Hoyt KJ, Han J, Grom AA, Gattorno M, Ravelli A,de Benedetti F, Behrens EM, Cron RQ, Nigrovic PA. On the alert for cytokinestorm: Immunopathology in COVID-19. Arthritis Rheumatol. 2020 Apr 15. doi:10.1002/art.41285. [Epub ahead of print] PubMed PMID: 32293098.	No appropriate outcome
66: Marongiu F, Grandone E, Barcellona D. Pulmonary thrombosis in 2019-nCoVpneumonia? J Thromb Haemost. 2020 Apr 15. doi: 10.1111/jth.14818. [Epub ahead of print] PubMed PMID: 32293083.	No appropriate population sample
67: Breslin N, Baptiste C, Gyamfi-Bannerman C, Miller R, Martinez R, Bernstein K,Ring L, Landau R, Purisch S, Friedman AM, Fuchs K, Sutton D, Andrikopoulou M,Rupley D, Sheen JJ, Aubey J, Zork N, Moroz L, Mourad M, Wapner R, Simpson LL,D'Alton ME, Goffman D. COVID-19 infection among asymptomatic and symptomaticpregnant women: Two weeks of confirmed presentations to an affiliated pair of NewYork City hospitals. Am J Obstet Gynecol MFM. 2020 Apr 9:100118. doi:10.1016/j.ajogmf.2020.100118. [Epub ahead of print] PubMed PMID: 32292903; PubMed	Included
68: Di Mascio D, Khalil A, Saccone G, Rizzo G, Buca D, Liberati M, Vecchiet J,Nappi L, Scambia G, Berghella V, D'Antonio F. Outcome of Coronavirus spectruminfections (SARS, MERS, COVID 1 -19) during pregnancy: a systematic review andmeta-analysis. Am J Obstet Gynecol MFM. 2020 Mar 25:100107.	No original article

doi:10.1016/j.ajogmf.2020.100107. [Epub ahead of print] Review. PubMed PMID:32292902; PubMed Central PMCID: PMC7104131.	
69: Raoult D, Zumla A, Locatelli F, Ippolito G, Kroemer G. Coronavirusinfections: Epidemiological, clinical and immunological features and hypotheses. Cell Stress. 2020 Mar 2;4(4):66-75. doi: 10.15698/cst2020.04.216. PubMed PMID:32292881; PubMed Central PMCID: PMC7146059.	No original article
70: Fan L, Li D, Xue H, Zhang L, Liu Z, Zhang B, Zhang L, Yang W, Xie B, Duan X, Hu X, Cheng K, Peng L, Yu N, Song L, Chen H, Sui X, Zheng N, Liu S, Jin Z.Progress and prospect on imaging diagnosis of COVID-19. Chin J Acad Radiol. 2020 Mar 18:1-10. doi: 10.1007/s42058-020-00031-5. [Epub ahead of print] Review.PubMed PMID: 32292880; PubMed Central PMCID: PMC7149076.	No appropriate outcome
71: Zanon D, Manca A, De Nicolò A, D'Avolio A, Musazzi UM, Cilurzo F, Maximova N,Tomasello C, Minghetti P. Data on the stability of darunavir/cobicistatsuspension after tablet manipulation. Data Brief. 2020 Apr 12:105552. doi:10.1016/j.dib.2020.105552. [Epub ahead of print] PubMed PMID: 32292805; PubMedCentral PMCID: PMC7152873.	No appropriate outcome
72: Omer S, Ali S, Babar ZUD. Preventive measures and management of COVID-19 inpregnancy. Drugs Ther Perspect. 2020 Apr 9:1-4. doi: 10.1007/s40267-020-00725-x. [Epub ahead of print] PubMed PMID: 32292265; PubMed Central PMCID: PMC7143201.	No original article
73: Shi B, Xia Z, Xiao S, Huang C, Zhou X, Xu H. Severe Pneumonia Due toSARS-CoV-2 and Respiratory Syncytial Virus Infection: A Case Report. Clin Pediatr(Phila). 2020 Apr 15:9922820920016. doi: 10.1177/0009922820920016. [Epub ahead ofprint] PubMed PMID: 32292053.	No appropriate population sample

74: Francis TJ. Waiting for COVID-19. J Paediatr Child Health. 2020 Apr 15. doi: 10.1111/jpc.14891. [Epub ahead of print] PubMed PMID: 32291858.	No appropriate population sample
75: Coletto LA, Favalli EG, Caporali R. Psoriasis and Psoriatic Arthritis: how tomanage Immunosuppressants in COVID-19 days. Dermatol Ther. 2020 Apr 14:e13415.doi: 10.1111/dth.13415. [Epub ahead of print] PubMed PMID: 32291828.	No appropriate population sample
76: Mu J, Xu J, Zhang L, Shu T, Wu D, Huang M, Ren Y, Li X, Geng Q, Xu Y, Qiu Y, Zhou X. SARS-CoV-2-encoded nucleocapsid protein acts as a viral suppressor of RNAinterference in cells. Sci China Life Sci. 2020 Apr 10. doi:10.1007/s11427-020-1692-1. [Epub ahead of print] PubMed PMID: 32291557.	No appropriate outcome
77: Duan YN, Zhu YQ, Tang LL, Qin J. CT features of novel coronavirus pneumonia(COVID-19) in children. Eur Radiol. 2020 Apr 14. doi: 10.1007/s00330-020-06860-3.[Epub ahead of print] Review. PubMed PMID: 32291501.	No original article
78: Mayor S. Covid-19: Nine in 10 pregnant women with infection when admitted fordelivery are asymptomatic, small study finds. BMJ. 2020 Apr 14;369:m1485. doi:10.1136/bmj.m1485. PubMed PMID: 32291260.	No original article
79: Quaedackers JSLT, Stein R, Bhatt N, Dogan HS, Hoen L, Nijman RJM, Radmayr C, Silay MS, Tekgul S, Bogaert G. Clinical and surgical consequences of the COVID-19pandemic for patients with pediatric urological problems: Statement of the EAUguidelines panel for paediatric urology, March 30 2020. J Pediatr Urol. 2020 Apr 9. pii: S1477-5131(20)30105-4. doi: 10.1016/j.jpurol.2020.04.007. [Epub ahead of print] PubMed PMID: 32291208; PubMed Central PMCID: PMC7144609.	No appropriate outcome

80: Parikh SR, Avansino JR, Dick AA, Enriquez BK, Geiduschek JM, Martin LD,McDonald RA, Yandow SM, Zerr DM, Ojemann JG. Collaborative Multi-DisciplinaryIncident Command at Seattle Children's Hospital for Rapid Preparatory PediatricSurgery Countermeasures to the COVID-19 Pandemic. J Am Coll Surg. 2020 Apr 11.pii: S1072-7515(20)30319-7. doi: 10.1016/j.jamcollsurg.2020.04.012. [Epub aheadof print] PubMed PMID: 32289376; PubMed Central PMCID: PMC7151263.	No original article
81: Ramos KJ, Pilewski JM, Faro A, Marshall BC. Improved Prognosis in CysticFibrosis: Consideration for Intensive Care During the COVID-19 Pandemic. Am JRespir Crit Care Med. 2020 Apr 14. doi: 10.1164/rccm.202004-0999LE. [Epub aheadof print] PubMed PMID: 32289235.	No appropriate population sample
82: Gudbjartsson DF, Helgason A, Jonsson H, Magnusson OT, Melsted P, Norddahl GL,Saemundsdottir J, Sigurdsson A, Sulem P, Agustsdottir AB, Eiriksdottir B,Fridriksdottir R, Gardarsdottir EE, Georgsson G, Gretarsdottir OS, GudmundssonKR, Gunnarsdottir TR, Gylfason A, Holm H, Jensson BO, Jonasdottir A, Jonsson F,Josefsdottir KS, Kristjansson T, Magnusdottir DN, le Roux L, Sigmundsdottir G,Sveinbjornsson G, Sveinsdottir KE, Sveinsdottir M, Thorarensen EA, Thorbjornsson B, Löve A, Masson G, Jonsdottir I, Möller AD, Gudnason T, Kristinsson KG,Thorsteinsdottir U, Stefansson K. Spread of SARS-CoV-2 in the IcelandicPopulation. N Engl J Med. 2020 Apr 14. doi: 10.1056/NEJMoa2006100. [Epub ahead ofprint] PubMed PMID: 32289214.	No appropriate outcome
83: Khoo EJ, Lantos JD. Lessons learned from the COVID-19 pandemic. ActaPaediatr. 2020 Apr 14. doi: 10.1111/apa.15307. [Epub ahead of print] PubMed PMID:32289175	No original article

84: Prokunina-Olsson L, Alphonse N, Dickenson RE, Durbin JE, Glenn JS, HartmannR, Kotenko SV, Lazear HM, O'Brien TR, Odendall C, Onabajo OO, Piontkivska H,Santer DM, Reich NC, Wack A, Zanoni I. COVID-19 and emerging viral infections:The case for interferon lambda. J Exp Med. 2020 May 4;217(5). pii: e20200653.doi: 10.1084/jem.20200653. PubMed PMID: 32289152.	No appropriate outcome
85: Matava CT, Kovatsis PG, Summers JL, Castro P, Denning S, Yu J, Lockman JL,Von Ungern-Sternberg B, Sabato S, Lee LK, Ayad I, Mireles S, Lardner D, Whyte S, Szolnoki J, Jagannathan N, Thompson N, Stein ML, Dalesio N, Greenberg R,McCloskey J, Peyton J, Evans F, Haydar B, Reynolds P, Chiao F, Taicher B,Templeton T, Bhalla T, Raman VT, Garcia-Marcinkiewicz A, Gálvez J, Tan J, Rehman M, Crockett C, Olomu P, Szmuk P, Glover C, Matuszczak M, Galvez I, Hunyady A,Polaner D, Gooden C, Hsu G, Gumaney H, Pérez-Pradilla C, Kiss EE, Theroux MC, LauJ, Asaf S, Ingelmo P, Engelhardt T, Hervías M, Greenwood E, Javia L, Disma N,Yaster M, Fiadjoe JE; PeDI-Collaborative. Pediatric Airway Management in COVID-19patients - Consensus Guidelines from the Society for Pediatric Anesthesia'sPediatric Difficult Intubation Collaborative and the Canadian PediatricAnesthesia Society. Anesth Analg. 2020 Apr 13. doi: 10.1213/ANE.0000000000004872.[Epub ahead of print] PubMed PMID: 32287142.	No original article
86: Curatola A, Ferretti S, Gatto A, Chiaretti A. Use of Handheld Transceiver forHospital Healthcare Workers-Caregiver Communication During the Coronavirusdisease 2019 (COVID-19) Outbreak in Pediatric Emergency Department. PediatrInfect Dis J. 2020 Apr 13. doi: 10.1097/INF.0000000000002689. [Epub ahead ofprint] PubMed PMID: 32287053.	No appropriate population sample

87: Robbins E, Ilahi Z, Roth P. Febrile Infant: COVID-19 in Addition to the UsualSuspects. Pediatr Infect Dis J. 2020 Apr 13. doi: 10.1097/INF.0000000000002693.[Epub ahead of print] PubMed PMID: 32287052.	No appropriate population sample
88: Parikh SR, Bly RA, Bonilla-Velez J, Dahl JP, Evans SS, Horn DL, Johnson KE,Manning SC, Ou HC, Pattisapu P, Perkins JA, Sie KCY. Pediatric OtolaryngologyDivisional and Institutional Preparatory Response at Seattle Children's Hospital after COVID-19 Regional Exposure. Otolaryngol Head Neck Surg. 2020 Apr14:194599820919748. doi: 10.1177/0194599820919748. [Epub ahead of print] PubMedPMID: 32286910.	No appropriate outcome
89: Moradi B, Kazemi MA, Gity M. CT Findings of Pregnant Women With CoronavirusDisease (COVID-19) Pneumonia. AJR Am J Roentgenol. 2020 Apr 14:W1. doi:10.2214/AJR.20.23212. [Epub ahead of print] PubMed PMID: 32286871.90: Golberstein E, Wen H, Miller BF. Coronavirus Disease 2019 (COVID-19) and	No original article
90: Golberstein E, Wen H, Miller BF. Coronavirus Disease 2019 (COVID-19) andMental Health for Children and Adolescents. JAMA Pediatr. 2020 Apr 14. doi:10.1001/jamapediatrics.2020.1456. [Epub ahead of print] PubMed PMID: 32286618.	No appropriate outcome
91: Henry BM, de Oliveira MHS, Benoit S, Plebani M, Lippi G. Hematologic,biochemical and immune biomarker abnormalities associated with severe illness andmortality in coronavirus disease 2019 (COVID-19): a meta-analysis. Clin Chem Lab Med. 2020 Apr 10. pii: /j/cclm.ahead-of-print/cclm-2020-0369/cclm-2020-0369.xml. doi: 10.1515/cclm-2020-0369. [Epub ahead of print] Review. PubMed PMID: 32286245.	No appropriate outcome
92: Infantino M, Damiani A, Gobbi FL, Grossi V, Lari B, Macchia D, Casprini P,Veneziani F, Villalta D, Bizzaro N, Cappelletti P, Fabris M, Quartuccio L,Benucci M, Manfredi M. Serological Assays for SARS-CoV-2 Infectious Disease:Benefits, Limitations and	No appropriate outcome

Perspectives. Isr Med Assoc J. 2020 Apr;22(4):203-210. PubMed PMID: 32286019.	
93: Cimolai N. Defining Protective Epitopes for COVID-19 Vaccination Models. JMed Virol. 2020 Apr 14. doi: 10.1002/jmv.25876. [Epub ahead of print] PubMedPMID: 32285942.	No appropriate outcome
94: Liu W, Wang J, Li W, Zhou Z, Liu S, Rong Z. Clinical characteristics of 19neonates born to mothers with COVID-19. Front Med. 2020 Apr 13. doi:10.1007/s11684-020-0772-y. [Epub ahead of print] PubMed PMID: 32285380.	Included
95: Lodha R, Kabra SK. COVID-19: How to Prepare for the Pandemic? Indian JPediatr. 2020 Apr 13. doi: 10.1007/s12098-020-03293-0. [Epub ahead of print]PubMed PMID: 32285326.	No original article
96: Joseph Davey D, Bekker LG, Coates TJ, Myer L. Contracting HIV or Contracting SAR-CoV-2 (COVID- 19) in Pregnancy? Balancing the Risks and Benefits. AIDS Behav.2020 Apr 13. doi: 10.1007/s10461-020-02861-x. [Epub ahead of print] PubMed PMID: 32285221	No appropriate outcome
97: Ambigapathy S, Rajahram GS, Shamsudin UK, Khoo EM, Cheah WK, Peariasamy KM,Goh PP, Khor SK. How should front-line general practitioners use personalprotective equipment (PPE)? Malays Fam Physician. 2020 Mar 18;15(1):2-5.eCollection 2020. Review. PubMed PMID: 32284798; PubMed Central PMCID:PMC7136675.	No appropriate population sample
98: Wu JT, Leung K, Bushman M, Kishore N, Niehus R, de Salazar PM, Cowling BJ,Lipsitch M, Leung GM. Estimating clinical severity of COVID-19 from thetransmission dynamics in Wuhan, China. Nat Med. 2020 Apr;26(4):506-510. doi:10.1038/s41591-020-0822-7. Epub 2020 Mar 19. PubMed PMID: 32284616.	No appropriate outcome

99: Xu Y, Li X, Zhu B, Liang H, Fang C, Gong Y, Guo Q, Sun X, Zhao D, Shen J,Zhang H, Liu H, Xia H, Tang J, Zhang K, Gong S. Characteristics of pediatricSARS-CoV-2 infection and potential evidence for persistent fecal viral shedding. Nat Med. 2020 Apr;26(4):502-505. doi: 10.1038/s41591-020-0817-4. Epub 2020 Mar13. PubMed PMID: 32284613.	No appropriate outcome
100: Mishra D. COVID-19 and Indian Pediatrics. Indian Pediatr. 2020 Apr15;57(4):287. PubMed PMID: 32284470.	No appropriate population sample
101: Body R, Carlton E, Carley S, Dawood M, Leech C, Smith SS, Weber EJ. Globalcrisis: EM and EMJ respond. Emerg Med J. 2020 Apr 13. pii: emermed-2020-209679.doi: 10.1136/emermed-2020-209679. [Epub ahead of print]	No appropriate population sample
102: Helmers A. Stability and Viability of SARS-CoV-2. N Engl J Med. 2020 Apr13;382. pii: 10.1056/NEJMc2007942#sa4. doi: 10.1056/NEJMc2007942. [Epub ahead of print] PubMed PMID: 32283578.	No appropriate outcome
103: Hedrich CM. COVID-19 - Considerations for the paediatric rheumatologist.Clin Immunol. 2020 Apr 10;214:108420. doi: 10.1016/j.clim.2020.108420. [Epubahead of print] Review. PubMed PMID: 32283324; PubMed Central PMCID: PMC7151358.	No appropriate population sample
104: Karami P, Naghavi M, Feyzi A, Aghamohammadi M, Novin MS, Mobaien A,Qorbanisani M, Karami A, Norooznezhad AH. Mortality of a pregnant patientdiagnosed with COVID-19: A case report with clinical, radiological, andhistopathological findings. Travel Med Infect Dis. 2020 Apr 11:101665. doi:10.1016/j.tmaid.2020.101665. [Epub ahead of print] PubMed PMID: 32283217; PubMed Central PMCID: PMC7151464.	Included

105: Hamadani M, Zhang MJ, Tang XY, Fei M, Brunstein C, Chhabra S, D'Souza A,Milano F, Phelan R, Saber W, Shaw BE, Weisdorf D, Devine SM, Horowitz MM. Graftcryopreservation does not impact overall survival allogeneic hematopoietic celltransplantation using post-transplant cyclophosphamide for GVHD prophylaxis. BiolBlood Marrow Transplant. 2020 Apr 10. pii: S1083-8791(20)30209-3. doi:10.1016/j.bbmt.2020.04.001. [Epub ahead of print] PubMed PMID: 32283185.	No appropriate population sample
106: Ye Q, Wang B, Mao J. The pathogenesis and treatment of the `Cytokine Storm' in COVID-19. J Infect. 2020 Apr 10. pii: S0163-4453(20)30165-1. doi:10.1016/j.jinf.2020.03.037. [Epub ahead of print] Review. PubMed PMID: 32283152.	No appropriate outcome
107: Xing Y, Ni W, Wu Q, Li W, Li G, Wang W, Tong J, Song X, Wong GWK, Xing Q.Dynamics of Faecal SARS-CoV-2 in Infected Children during the Convalescent Phase.J Infect. 2020 Apr 10. pii: S0163-4453(20)30177-8. doi:10.1016/j.jinf.2020.03.049. [Epub ahead of print] PubMed PMID: 32283149.	No appropriate outcome
108: Ashokka B, Loh MH, Tan CH, Su LL, Young BE, Lye DC, Biswas A, E Illanes S,Choolani M. Care of the Pregnant Woman with COVID-19 in Labor and Delivery:Anesthesia, Emergency cesarean delivery, Differential diagnosis in the acutelyill parturient, Care of the newborn, and Protection of the healthcare personnel. Am J Obstet Gynecol. 2020 Apr 10. pii: S0002-9378(20)30430-0. doi:10.1016/j.ajog.2020.04.005. [Epub ahead of print] PubMed PMID: 32283073; PubMedCentral PMCID: PMC7151436.	No original article
109: Cheng MP, Papenburg J, Desjardins M, Kanjilal S, Quach C, Libman M, DittrichS, Yansouni CP. Diagnostic Testing for Severe Acute Respiratory Syndrome-Related Coronavirus-2: A Narrative Review. Ann Intern Med. 2020 Apr 13. doi:10.7326/M20-	No original article

1301. [Epub ahead of print] PubMed PMID: 32282894.	
110: Wang H, Li Y, Wang F, Du H, Lu X. Rehospitalization of a RecoveredCoronavirus Disease 19 (COVID-19) Child With Positive Nucleic Acid Detection.Pediatr Infect Dis J. 2020 Apr 9. doi: 10.1097/INF.0000000000002690. [Epub ahead of print] PubMed PMID: 32282658.	No appropriate population sample
111: Berg EA, Picoraro JA, Miller SD, Srinath A, Franciosi JP, Hayes CE, Farrell PR, Cole CR, LeLeiko NS. COVID-19 - A Guide to Rapid Implementation of TelehealthServices: A Playbook for the Pediatric Gastroenterologist. J PediatrGastroenterol Nutr. 2020 Apr 10. doi: 10.1097/MPG.0000000000002749. [Epub aheadof print] PubMed PMID: 32282628.	No appropriate outcome
112: Verstraete SG, Sola AM, Ali SA. Telemedicine for Pediatric Inflammatorybowel disease in the Era of COVID-19. J Pediatr Gastroenterol Nutr. 2020 Apr 8.doi: 10.1097/MPG.0000000000002747. [Epub ahead of print] PubMed PMID: 32282624.	No appropriate outcome
113: Lippi G, de Oliveira MHS, Henry BM. Chronic liver disease is not associated with severity or mortality in Coronavirus disease 2019 (COVID-19): a pooledanalysis. Eur J Gastroenterol Hepatol. 2020 Apr 10. doi:10.1097/MEG.0000000000001742. [Epub ahead of print] PubMed PMID: 32282549.	No appropriate population sample
114: Pathak EB, Salemi JL, Sobers N, Menard J, Hambleton IR. COVID-19 in Childrenin the United States: Intensive Care Admissions, Estimated Total Infected, andProjected Numbers of Severe Pediatric Cases in 2020. J Public Health Manag Pract.2020 Apr 10. doi: 10.1097/PHH.0000000000001190. [Epub ahead of print] PubMedPMID: 32282440.	No appropriate population sample

115: DeBaun MR. Initiating adjunct low dose-hydroxyurea therapy for strokeprevention in children with SCA during the COVID-19 pandemic. Blood. 2020 Apr 13.pii: blood.2020005992. doi: 10.1182/blood.2020005992. [Epub ahead of print]PubMed PMID: 32282033.	No appropriate population sample
116: Syal K. COVID-19: Herd Immunity and Convalescent Plasma Transfer Therapy. J Med Virol. 2020 Apr 13. doi: 10.1002/jmv.25870. [Epub ahead of print] PubMedPMID: 32281679.	No appropriate outcome
117: Schiariti V. Prawa człowieka dzieci niepełnosprawnych w sytuacjachkryzysowych: wyzwanie COVID-19. Dev Med Child Neurol. 2020 Apr 13. doi:10.1111/dmcn.14544. [Epub ahead of print] PubMed PMID: 32281649.	No appropriate population sample
118: Bradbury-Jones C, Isham L. The pandemic paradox: the consequences ofCOVID-19 on domestic violence. J Clin Nurs. 2020 Apr 12. doi: 10.1111/jocn.15296.[Epub ahead of print] PubMed PMID: 32281158.	No appropriate population sample
119: Caccialanza R, Laviano A, Lobascio F, Montagna E, Bruno R, Ludovisi S,Corsico AG, Di Sabatino A, Belliato M, Calvi M, Iacona I, Grugnetti G, Bonadeo E,Muzzi A, Cereda E. Early nutritional supplementation in non-critically illpatients hospitalized for the 2019 novel coronavirus disease (COVID-19):Rationale and feasibility of a shared pragmatic protocol. Nutrition. 2020 Apr3:110835. doi: 10.1016/j.nut.2020.110835. [Epub ahead of print] PubMed PMID:32280058	No appropriate population sample
120: Khan S, Peng L, Siddique R, Nabi G, Nawsherwan, Xue M, Liu J, Han G. Impact of COVID-19 infection on pregnancy outcomes and the risk of maternal-to-neonatal intrapartum transmission of COVID-19 during natural birth. Infect Control HospEpidemiol. 2020 Mar 19:1-3. doi: 10.1017/ice.2020.84. [Epub ahead of	Included

print]PubMed PMID: 32279693; PubMed Central PMCID: PMC7156579.	
121: Huang T, Guo Y, Li S, Zheng Y, Lei L, Zeng X, Zhong Q, Liu Y, Liu L.Application and effects of fever screening system in the prevention of nosocomialinfection in the only designated hospital of coronavirus disease 2019 (COVID-19) in Shenzhen, China. Infect Control Hosp Epidemiol. 2020 Apr 13:1-10. doi:10.1017/ice.2020.119. [Epub ahead of print] PubMed PMID: 32279675.	No appropriate outcome
122: Sarialioglu F, Belen Apak FB, Haberal M. Can Hepatitis A Vaccine ProvideProtection Against COVID-19? Exp Clin Transplant. 2020 Apr;18(2):141-143. doi:10.6002/ect.2020.0109. Review. PubMed PMID: 32279655.	No appropriate outcome
123: Bartiromo M, Borchi B, Botta A, Bagalà A, Lugli G, Tilli M, Cavallo A,Xhaferi B, Cutruzzulà R, Vaglio A, Bresci S, Larti A, Bartoloni A, Cirami C.Threatening drug-drug interaction in a kidney transplant patient with CoronavirusDisease 2019 (COVID-19). Transpl Infect Dis. 2020 Apr 12. doi: 10.1111/tid.13286.[Epub ahead of print] PubMed PMID: 32279418.	No appropriate population sample
124: Dayal D. We urgently need guidelines for managing COVID-19 in children with comorbidities. Acta Paediatr. 2020 Apr 12. doi: 10.1111/apa.15304. [Epub ahead ofprint] PubMed PMID: 32279351.	No original article
125: Matthai J, Shanmugam N, Sobhan P; Indian Society Of PediatricGastroenterology, Hepatology And Nutrition; Pediatric Gastroenterology Chapter OfIndian Academy Of Pediatrics . Coronavirus Disease (COVID-19) and theGastrointestinal System in Children. Indian Pediatr. 2020 Apr 12. pii:S097475591600162. [Epub ahead of print] PubMed PMID: 32279064.	No appropriate outcome

126: Henry BM, Lippi G. Poor survival with extracorporeal membrane oxygenation inacute respiratory distress syndrome (ARDS) due to coronavirus disease 2019(COVID-19): Pooled analysis of early reports. J Crit Care. 2020 Apr 1;58:27-28.doi: 10.1016/j.jcrc.2020.03.011. [Epub ahead of print] PubMed PMID: 32279018;PubMed Central PMCID: PMC7118619.	No appropriate outcome
127: Penel N, Bonvalot S, Minard V, Orbach D, Gouin F, Corradini N, Brahmi M,Marec-Bérard P, Briand S, Gaspar N, Llacer C, Carrère S, Dufresne A, Le Cesne A, Blay JY. French Sarcoma Group proposals for management of sarcoma patients duringCOVID-19 outbreak. Ann Oncol. 2020 Apr 9. pii: S0923-7534(20)36413-9. doi:10.1016/j.annonc.2020.03.308. [Epub ahead of print] PubMed PMID: 32278878; PubMedCentral PMCID: PMC7144615.	No appropriate population sample
128: Lancaster EM, Sosa JA, Sammann A, Pierce L, Shen W, Conte M, Wick E. RapidResponse of an Academic Surgical Department to the COVID-19 Pandemic:Implications for Patients, Surgeons, and the Community. J Am Coll Surg. 2020 Apr 9. pii: S1072-7515(20)30312-4. doi: 10.1016/j.jamcollsurg.2020.04.007. [Epubahead of print] PubMed PMID: 32278726.	No appropriate outcome
129: Saccone G, Carbone FI, Zullo F. The novel coronavirus (2019-nCoV) inpregnancy: What we need to know. Eur J Obstet Gynecol Reprod Biol. 2020 Apr 2.pii: S0301-2115(20)30174-3. doi: 10.1016/j.ejogrb.2020.04.006. [Epub ahead ofprint] PubMed PMID: 32278619; PubMed Central PMCID: PMC7131203.	No original article
130: Leva E, Morandi A, Sartori A, Macchini F, Berrettini A, Manzoni G.Correspondence from Northern Italy about our experience with COVID-19. J Pediatr Surg. 2020 Apr 1. pii: S0022-3468(20)30252-9. doi:10.1016/j.jpedsurg.2020.03.028. [Epub ahead of print] PubMed PMID: 32278542.	No original article

131: Gligorov J, Bachelot T, Pierga JY, Antoine EC, Balleyguier C, Barranger E,Belkacemi Y, Bonnefoi H, Bidard FC, Ceugnart L, Classe JM, Cottu P, Coutant C,Cutuli B, Dalenc F, Darai E, Dieras V, Dohollou N, Giacchetti S, Goncalves A,Hardy-Bessard AC, Houvenaeghel G, Jacquin JP, Jacot W, Levy C, Mathelin C, NisandI, Petit T, Petit T, Poncelet E, Rivera S, Rouzier R, Salmon R, Scotté F, SpanoJP, Uzan C, Zelek L, Spielmann M, Penault-Llorca F, Namer M, Delaloge S.[COVID-19 and people followed for breast cancer: French guidelines for clinicalpractice of Nice-St Paul de Vence, in collaboration with the Collège Nationaledes Gynécologues et Obstétriciens Français (CNGOF), the Société d'Imagerie de la FEMme (SIFEM), the Société Française de Chirurgie Oncologique (SFCO), the SociétéFrançaise de Sénologie et Pathologie Mammaire (SFSPM) and the French BreastCancer Intergroup-UNICANCER (UCBG)]. Bull Cancer. 2020 Apr 1. pii:S0007-4551(20)30159-4. doi: 10.1016/j.bulcan.2020.03.008. [Epub ahead of print]French. PubMed PMID: 32278467; PubMed Central PMCID: PMC7118684.	No appropriate population sample
132: Lazzerini M, Barbi E, Apicella A, Marchetti F, Cardinale F, Trobia G.Delayed access or provision of care in Italy resulting from fear of COVID-19.Lancet Child Adolesc Health. 2020 Apr 9. pii: S2352-4642(20)30108-5. doi:10.1016/S2352-4642(20)30108-5. [Epub ahead of print] PubMed PMID: 32278365.	No appropriate outcome
133: Martinez-Portilla RJ, Goncé A, Hawkins-Villarreal A, Figueras F. A Spanish-translated clinical algorithm for management of suspected SARS-CoV-2infection in pregnant women. Lancet Infect Dis. 2020 Apr 9. pii:S1473-3099(20)30285-1. doi: 10.1016/S1473-3099(20)30285-1. [Epub ahead of print] PubMed PMID: 32278360; PubMed Central PMCID: PMC7158943.	No original article
134: Moazzami B, Razavi-Khorasani N, Dooghaie Moghadam A, Farokhi E, Rezaei N.COVID-19 and telemedicine: Immediate action required for maintaining healthcareproviders well-being. J Clin Virol.	No appropriate outcome

2020 Apr 4;126:104345. doi:10.1016/j.jcv.2020.104345. [Epub ahead of print] PubMed PMID: 32278298; PubMedCentral PMCID: PMC7129277.	
135: Frauenfelder C, Butler C, Hartley B, Cochrane L, Jephson C, Nash R, HewittR, Albert D, Wyatt M, Hall A. Practical insights for paediatric otolaryngologysurgical cases and performing microlaryngobronchoscopy during the COVID-19pandemic. Int J Pediatr Otorhinolaryngol. 2020 Mar 30;134:110030. doi:10.1016/j.ijporl.2020.110030. [Epub ahead of print] PubMed PMID: 32278168; PubMedCentral PMCID: PMC7142686.	No appropriate outcome
136: Khan S, Jun L, Nawsherwan, Siddique R, Li Y, Han G, Xue M, Nabi G, Liu J.Association of COVID-19 infection with pregnancy outcomes in healthcare workers and general women. Clin Microbiol Infect. 2020 Apr 8. pii: S1198-743X(20)30180-4.doi: 10.1016/j.cmi.2020.03.034. [Epub ahead of print] PubMed PMID: 32278081;PubMed Central PMCID: PMC7141623.	Overlapping data
137: Shah PS, Diambomba Y, Acharya G, Morris SK, Bitnun A. Classification system and case definition for SARS-CoV-2 infection in pregnant women, fetuses, andneonates. Acta Obstet Gynecol Scand. 2020 Apr 11. doi: 10.1111/aogs.13870. [Epub ahead of print] PubMed PMID: 32277845.	No original article
138: Danis K, Epaulard O, Bénet T, Gaymard A, Campoy S, Bothelo-Nevers E,Bouscambert-Duchamp M, Spaccaferri G, Ader F, Mailles A, Boudalaa Z, Tolsma V,Berra J, Vaux S, Forestier E, Landelle C, Fougere E, Thabuis A, Berthelot P, VeilR, Levy-Bruhl D, Chidiac C, Lina B, Coignard B, Saura C; Investigation Team .Cluster of coronavirus disease 2019 (Covid-19) in the French Alps, 2020. ClinInfect Dis. 2020 Apr 11. pii: ciaa424. doi: 10.1093/cid/ciaa424. [Epub ahead ofprint] PubMed PMID: 32277759.	No appropriate population sample

139: Meena J, Kumar J. Fecal Shedding of SARS CoV-2: Implications for DiseaseSpread and Quarantine. Indian Pediatr. 2020 Apr 11. pii: S097475591600160. [Epub ahead of print] PubMed PMID: 32277746.	No appropriate outcome
140: Wilson CG, Ramage M, Fagan EB. A Primary Care Response to COVID-19 forPatients with an Opioid Use Disorder. J Rural Health. 2020 Apr 11. doi:10.1111/jrh.12438. [Epub ahead of print] PubMed PMID: 32277732.	No appropriate population sample
141: Yousefzadegan S, Rezaei N. Case Report: Death Due to Novel CoronavirusDisease (COVID-19) in Three Brothers. Am J Trop Med Hyg. 2020 Apr 10. doi:10.4269/ajtmh.20-0240. [Epub ahead of print] PubMed PMID: 32277694.	No appropriate outcome
142: Deprest J, Van Ranst M, Lannoo L, Bredaki E, Ryan G, David A, Richter J, VanMieghem T. Feto-placental surgeries during the covid-19 pandemic: starting thediscussion. Prenat Diagn. 2020 Apr 11. doi: 10.1002/pd.5702. [Epub ahead ofprint] PubMed PMID: 32277490.	No appropriate outcome
144: Liu H, He X, Wang Y, Zhou S, Zhang D, Zhu J, He Q, Zhu Z, Li G, Sun L, Wang J, Cheng G, Liu Z, Lau G. Management of COVID-19 in patients after livertransplantation: Beijing working party for liver transplantation. Hepatol Int.2020 Apr 10. doi: 10.1007/s12072-020-10043-z. [Epub ahead of print] PubMed PMID: 32277387	No appropriate population sample
145: Mimouni F, Lakshminrusimha S, Pearlman SA, Raju T, Gallagher PG, MendlovicJ. Perinatal aspects on the covid-19 pandemic: a practical resource forperinatal-neonatal specialists. J Perinatol. 2020 Apr 10. doi:10.1038/s41372-020-0665-6. [Epub ahead of print] Review. PubMed PMID: 32277162.	No original article

146: Solnica A, Barski L, Jotkowitz A. Allocation of scarce resources during the COVID-19 pandemic: a Jewish ethical perspective. J Med Ethics. 2020 Apr 10. pii: medethics-2020-106242. doi: 10.1136/medethics-2020-106242. [Epub ahead of print] PubMed PMID: 32277021.	No appropriate outcome
147: Lee CY, Wang PS, Huang YD, Lin YC, Hsu YN, Chen SC. Evacuation ofquarantine-qualified nationals from Wuhan for COVID-19 outbreak - Taiwanexperience. J Microbiol Immunol Infect. 2020 Apr 1. pii: S1684-1182(20)30083-9.doi: 10.1016/j.jmii.2020.03.023. [Epub ahead of print] PubMed PMID: 32276849;PubMed Central PMCID: PMC7118679.	No appropriate outcome
148: Xing YH, Ni W, Wu Q, Li WJ, Li GJ, Wang WD, Tong JN, Song XF, Wing-Kin Wong G, Xing QS. Prolonged viral shedding in feces of pediatric patients withcoronavirus disease 2019. J Microbiol Immunol Infect. 2020 Mar 28. pii:S1684-1182(20)30081-5. doi: 10.1016/j.jmii.2020.03.021. [Epub ahead of print]Review. PubMed PMID: 32276848; PubMed Central PMCID: PMC7141453.	No appropriate population sample
149: Dipasquale V, Cucchiara S, Martinelli M, Miele E, Aloi M, Romano C.Challenges in paediatric inflammatory bowel diseases in the COVID-19 time. DigLiver Dis. 2020 Apr 7. pii: S1590-8658(20)30117-1. doi:10.1016/j.dld.2020.03.015. [Epub ahead of print] PubMed PMID: 32276846; PubMedCentral PMCID: PMC7141464.	No appropriate outcome
150: Wilson AN, Ravaldi C, Scoullar MJL, Vogel JP, Szabo RA, Fisher JRW, HomerCSE. Caring for the carers: Ensuring the provision of quality maternity careduring a global pandemic. Women Birth. 2020 Apr 8. pii: S1871-5192(20)30212-2.doi: 10.1016/j.wombi.2020.03.011. [Epub ahead of print] PubMed PMID: 32276778;PubMed Central PMCID: PMC7141547.	No appropriate outcome

151: Casamassimo PS, Townsend JA, Litch CS. Pediatric Dentistry During and After COVID-19. Pediatr Dent. 2020 Mar 15;42(2):87-90. PubMed PMID: 32276673.	No appropriate population sample
187: Li L, Zhang B, He B, Gong Z, Chen X. Critical patients with coronavirusdisease 2019: Risk factors and outcome nomogram. J Infect. 2020 Apr 6. pii:S0163-4453(20)30153-5. doi: 10.1016/j.jinf.2020.03.025. [Epub ahead of print]PubMed PMID: 32272120.	No appropriate population sample
278: Eccleston C, Blyth FM, Dear BF, Fisher EA, Keefe FJ, Lynch ME, Palermo TM,Reid MC, Williams ACC. Managing patients with chronic pain during the COVID-19outbreak: considerations for the rapid introduction of remotely supported(eHealth) pain management services. Pain. 2020 May;161(5):889-893. doi:10.1097/j.pain.0000000000001885. PubMed PMID: 32251203.	No appropriate population sample
1: Palatnik A, McIntosh JJ. Protecting Labor and Delivery Personnel from COVID-19during the Second Stage of Labor. Am J Perinatol. 2020 Apr 10. doi:10.1055/s-0040-1709689. [Epub ahead of print] PubMed PMID: 32276282.	No original article
2: Wellons JC, Grant G, Krieger MD, Ragheb J, Robinson S, Weprin B, Ojemann J.Editorial. Early lessons in the management of COVID-19 for the pediatricneurosurgical community from the leadership of the American Society of Pediatric Neurosurgeons. J Neurosurg Pediatr. 2020 Apr 10:1-2. doi:10.3171/2020.3.PEDS20215. [Epub ahead of print] PubMed PMID: 32276256.	No appropriate outcome
3: Sawalha AH, Zhao M, Coit P, Lu Q. Epigenetic dysregulation of ACE2 andinterferon-regulated genes might suggest increased COVID-19 susceptibility andseverity in lupus patients. Clin Immunol. 2020 Apr 7:108410. doi:10.1016/j.clim.2020.108410. [Epub ahead of print] PubMed PMID: 32276140.	No appropriate population sample

4: Yan C, Cui J, Huang L, Du B, Chen L, Xue G, Li S, Zhang W, Zhao L, Sun Y, Yao H, Li N, Zhao H, Feng Y, Liu S, Zhang Q, Liu D, Yuan J. Rapid and visualdetection of 2019 novel coronavirus (SARS-CoV-2) by a reverse transcriptionloop-mediated isothermal amplification assay. Clin Microbiol Infect. 2020 Apr 7. pii: S1198-743X(20)30186-5. doi: 10.1016/j.cmi.2020.04.001. [Epub ahead of print]PubMed PMID: 32276116.	No appropriate outcome
5: Rubin GD, Ryerson CJ, Haramati LB, Sverzellati N, Kanne JP, Raoof S, Schluger NW, Volpi A, Yim JJ, Martin IBK, Anderson DJ, Kong C, Altes T, Bush A, Desai SR, Goldin J, Goo JM, Humbert M, Inoue Y, Kauczor HU, Luo F, Mazzone PJ, Prokop M,Remy-Jardin M, Richeldi L, Schaefer-Prokop CM, Tomiyama N, Wells AU, Leung AN.The Role of Chest Imaging in Patient Management during the COVID-19 Pandemic: AMultinational Consensus Statement from the Fleischner Society. Chest. 2020 Apr 7.pii: S0012-3692(20)30673-5. doi: 10.1016/j.chest.2020.04.003. [Epub ahead ofprint] PubMed PMID: 32275978.	No original article
6: Van Lancker W, Parolin Z. COVID-19, school closures, and child poverty: asocial crisis in the making. Lancet Public Health. 2020 Apr 7. pii:S2468-2667(20)30084-0. doi: 10.1016/S2468-2667(20)30084-0. [Epub ahead of print] PubMed PMID: 32275858.	No appropriate outcome
7: Joob B, Wiwanitkit V. COVID-19 , School Closings and Weight Gain. Obesity(Silver Spring). 2020 Apr 10. doi: 10.1002/oby.22825. [Epub ahead of print]PubMed PMID: 32275803.	No appropriate outcome
8: Dario Mandato V, Aguzzoli L. Management of ovarian cancer during the COVID-19 pandemic. Int J Gynaecol Obstet. 2020 Apr 10. doi: 10.1002/ijgo.13167. [Epubahead of print] PubMed PMID: 32275775.	No appropriate population sample

9: Hotez PJ, Bottazzi ME, Singh SK, Brindley PJ, Kamhawi S. Will COVID-19 become the next neglected tropical disease? PLoS Negl Trop Dis. 2020 Apr10;14(4):e0008271. doi: 10.1371/journal.pntd.0008271. eCollection 2020 Apr.PubMed PMID: 32275667.	No appropriate outcome
10: Tavazzi G, Pellegrini C, Maurelli M, Belliato M, Sciutti F, Bottazzi A, Sepe PA, Resasco T, Camporotondo R, Bruno R, Baldanti F, Paolucci S, Pelenghi S, IottiGA, Mojoli F, Arbustini E. Myocardial localization of coronavirus in COVID-19cardiogenic shock. Eur J Heart Fail. 2020 Apr 10. doi: 10.1002/ejhf.1828. [Epubahead of print] PubMed PMID: 32275347.	No appropriate outcome
11: Hartley DM, Perencevich EN. Public Health Interventions for COVID-19:Emerging Evidence and Implications for an Evolving Public Health Crisis. JAMA.2020 Apr 10. doi: 10.1001/jama.2020.5910. [Epub ahead of print] PubMed PMID:32275299	No original article
12: Pan A, Liu L, Wang C, Guo H, Hao X, Wang Q, Huang J, He N, Yu H, Lin X, WeiS, Wu T. Association of Public Health Interventions With the Epidemiology of the COVID-19 Outbreak in Wuhan, China. JAMA. 2020 Apr 10. doi:10.1001/jama.2020.6130. [Epub ahead of print] PubMed PMID: 32275295.	No appropriate outcome
13: Sinha A. King Lear Under COVID-19 Lockdown. JAMA. 2020 Apr 10. doi:10.1001/jama.2020.6186. [Epub ahead of print] PubMed PMID: 32275290.	No original article
14: Rabaan AA, Al-Ahmed SH, Haque S, Sah R, Tiwari R, Malik YS, Dhama K, YatooMI, Bonilla-Aldana DK, Rodriguez-Morales AJ. Exploring the genetics, ecology ofSARS-CoV-2 and climatic factors as possible control strategies against COVID-19. Infez Med. 2020 Ahead of print Jun 1;28(2):174-184. PubMed PMID: 32275259.	No original article

15: Cossarizza A, Gibellini L, De Biasi S, Lo Tartaro D, Mattioli M, Paolini A,Fidanza L, Bellinazzi C, Borella R, Castaniere I, Meschiari M, Sita M, Manco G,Clini E, Gelmini R, Girardis M, Guaraldi G, Mussini C. Handling and Processing ofBlood Specimens from Patients with COVID-19 for Safe Studies on Cell Phenotypeand Cytokine Storm. Cytometry A. 2020 Apr 10. doi: 10.1002/cyto.a.24009. [Epubahead of print] PubMed PMID: 32275124.	No appropriate outcome
16: de Ángel Solá DE, Wang L, Vázquez M, Méndez Lázaro PA. Weathering thepandemic: How the Caribbean Basin can use viral and environmental patterns topredict, prepare and respond to COVID-19. J Med Virol. 2020 Apr 10. doi:10.1002/jmv.25864. [Epub ahead of print] Review. PubMed PMID: 32275090.	No appropriate outcome
17: Lippi G, Mattiuzzi C, Sanchis-Gomar F, Henry BM. Clinical and demographic characteristics of patients dying from COVID-19 in Italy versus China. J MedVirol. 2020 Apr 10. doi: 10.1002/jmv.25860. [Epub ahead of print] PubMed PMID:32275075	No appropriate outcome
18: He G, Sun W, Fang P, Huang J, Gamber M, Cai J, Wu J. The clinical feature of silent infections of novel coronavirus infection (COVID-19) in Wenzhou. J MedVirol. 2020 Apr 10. doi: 10.1002/jmv.25861. [Epub ahead of print] PubMed PMID:32275074	No appropriate population sample
19: Xiong X, Wei H, Zhang Z, Chang J, Ma X, Gao X, Chen Q, Pang Q. VaginalDelivery Report of a Healthy Neonate Born to a Convalescent Mother with COVID-19.J Med Virol. 2020 Apr 10. doi: 10.1002/jmv.25857. [Epub ahead of print] PubMedPMID: 32275072.	Included
20: Porpiglia F, Checcucci E, Amparore D, Verri P, Campi R, Claps F, Esperto F,Fiori C, Carrieri G, Ficarra V, Scarpa RM, Dasgupta P; European Society ofResidents in Urology (ESRU) of European Association of Urology (EAU). Slowdown ofurology residents' learning curve during COVID-19 emergency. BJU Int. 2020 Apr 9.doi: 10.1111/bju.15076.	No appropriate population sample

[Epub ahead of print] PubMed PMID: 32274879.	
21: Kranke P, Weibel S, Sitter M, Meybohm P, Girard T. [Obstetric AnesthesiaDuring the SARS-CoV-2 Pandemic - a Brief Overview of Published Recommendationsfor Action by National and International Specialist Societies and Committees].Anasthesiol Intensivmed Notfallmed Schmerzther. 2020 Apr;55(4):266-274. doi:10.1055/a-1144-5562. Epub 2020 Apr 9. German. PubMed PMID: 32274774.	No original article
22: McIntosh JJ. Corticosteroid Guidance for Pregnancy during COVID-19 Pandemic. Am J Perinatol. 2020 Apr 9. doi: 10.1055/s-0040-1709684. [Epub ahead of print]PubMed PMID: 32274772.	No original article
23: Gonzalez-Brown VM, Reno J, Lortz H, Fiorini K, Costantine MM. Operating Room Guide for Confirmed or Suspected COVID-19 Pregnant Patients Requiring CesareanDelivery. Am J Perinatol. 2020 Apr 9. doi: 10.1055/s-0040-1709683. [Epub ahead ofprint] PubMed PMID: 32274771.	No original article
24: Li J, Fan JG. Characteristics and Mechanism of Liver Injury in 2019Coronavirus Disease. J Clin Transl Hepatol. 2020 Mar 28;8(1):13-17. doi:10.14218/JCTH.2020.00019. Epub 2020 Mar 30. Review. PubMed PMID: 32274341; PubMedCentral PMCID: PMC7132021.	No appropriate outcome
25: Lloyd R. Six ways to juggle science and childcare from home. Nature. 2020 Apr9. doi: 10.1038/d41586-020-01060-x. [Epub ahead of print] PubMed PMID: 32273622.	No appropriate outcome
26: Rossman H, Keshet A, Shilo S, Gavrieli A, Bauman T, Cohen O, Shelly E,Balicer R, Geiger B, Dor Y, Segal E. A framework for identifying regionaloutbreak and spread of COVID-19 from one-minute population-wide	No appropriate outcome

surveys. Nat Med.2020 Apr 9. doi: 10.1038/s41591-020-0857-9. [Epub ahead of print] PubMed PMID:32273611	
27: Stower H. Clinical and epidemiological characteristics of children withCOVID-19. Nat Med. 2020 Apr 9. doi: 10.1038/s41591-020-0846-z. [Epub ahead ofprint] PubMed PMID: 32273605.	No appropriate population sample
28: Balasubramanian S, Rao NM, Goenka A, Roderick M, Ramanan AV. CoronavirusDisease (COVID-19) in Children - What We Know So Far and What We Do Not? IndianPediatr. 2020 Apr 9. pii: S097475591600159. [Epub ahead of print] PubMed PMID:32273490	No original article
29: Guiqing HE, Sun W, Jing WU, Cai J. Serial Computed Tomography Manifestations in a Child with Coronavirus Disease (COVID-19) Pneumonia. Indian Pediatr. 2020Apr 9. pii: S097475591600158. [Epub ahead of print] PubMed PMID: 32273489.	No appropriate population sample
30: Kulkarni RK, Kinikar AA, Chandanwale A. Impact of COVID-19 on Children andPediatricians. Indian Pediatr. 2020 Apr 9. pii: S097475591600157. [Epub ahead of print] PubMed PMID: 32273488.	No appropriate population sample
31: Vessey JA, Betz CL. Everything old is new again: COVID-19 and public health. J Pediatr Nurs. 2020 Mar 26. pii: S0882-5963(20)30205-0. doi:10.1016/j.pedn.2020.03.014. [Epub ahead of print] PubMed PMID: 32273143.	No appropriate outcome
32: Coccolini F, Sartelli M, Kluger Y, Pikoulis E, Karamagioli E, Moore EE, BifflWL, Peitzman A, Hecker A, Chirica M, Damaskos D, Ordonez C, Vega F, Fraga GP,Chiarugi M, Di Saverio S, Kirkpatrick AW, Abu-Zidan F, Mefire AC, Leppaniemi A,Khokha V, Sakakushev B, Catena R, Coimbra R, Ansaloni L, Corbella D, Catena F.COVID-19 the showdown for mass casualty preparedness and management: theCassandra Syndrome.	No appropriate population sample

World J Emerg Surg. 2020 Apr 9;15(1):26. doi:10.1186/s13017-020-00304-5. PubMed PMID: 32272957.	
33: Cianfarani S. Pediatric Endocrinology in the Time of the COVID-19 Pandemic.Horm Res Paediatr. 2020 Apr 9:1-2. doi: 10.1159/000507703. [Epub ahead of print] PubMed PMID: 32272473.	No appropriate population sample
34: Ghaysouri A, Sadeghifard N, Nazari A, Kalvandi G, Feizi J, Qasemi E,Kafashian M, Borji M, Kokhazadeh T, Tavan H. New clinical experiences andevaluation of clinical and paraclinical features of deceased patients withCOVID-19 infection referred to Shahid Mostafa Khomeini Hospital of Ilam, Iranin. Travel Med Infect Dis. 2020 Apr 6:101657. doi: 10.1016/j.tmaid.2020.101657. [Epubahead of print] PubMed PMID: 32272197.	No appropriate population sample
35: Viner RM, Russell SJ, Croker H, Packer J, Ward J, Stansfield C, Mytton O,Bonell C, Booy R. School closure and management practices during coronavirusoutbreaks including COVID-19: a rapid systematic review. Lancet Child AdolescHealth. 2020 Apr 6. pii: S2352-4642(20)30095-X. doi:10.1016/S2352-4642(20)30095-X. [Epub ahead of print] Review. PubMed PMID:32272089	No appropriate population sample
36: Bayefsky MJ, Bartz D, Watson KL. Abortion during the Covid-19 Pandemic - Ensuring Access to an Essential Health Service. N Engl J Med. 2020 Apr 9. doi:10.1056/NEJMp2008006. [Epub ahead of print] PubMed PMID: 32272002.	No appropriate population sample
37: Parazzini F, Bortolus R, Mauri PA, Favilli A, Gerli S, Ferrazzi E. Deliveryin pregnant women infected with SARS-CoV-2: A fast review. Int J Gynaecol Obstet.2020 Apr 9. doi: 10.1002/ijgo.13166. [Epub ahead of print] Review. PubMed PMID:32271947	No original article

38: Hooli S, King C. Generalizability of COVID-19 Clinical Prediction Models.Clin Infect Dis. 2020 Apr 9. pii: ciaa417. doi: 10.1093/cid/ciaa417. [Epub ahead of print] PubMed PMID: 32271865.	No appropriate outcome
39: CDC COVID-19 Response Team. Coronavirus Disease 2019 in Children - UnitedStates, February 12-April 2, 2020. MMWR Morb Mortal Wkly Rep. 2020 Apr10;69(14):422-426. doi: 10.15585/mmwr.mm6914e4. PubMed PMID: 32271728.	No appropriate outcome
40: Zwald ML, Lin W, Sondermeyer Cooksey GL, Weiss C, Suarez A, Fischer M, Bonin BJ, Jain S, Langley GE, Park BJ, Moulia D, Benedict R, Nguyen N, Han GS. RapidSentinel Surveillance for COVID-19 - Santa Clara County, California, March 2020. MMWR Morb Mortal Wkly Rep. 2020 Apr 10;69(14):419-421. doi:10.15585/mmwr.mm6914e3. PubMed PMID: 32271724.	No appropriate outcome
41: Stuebe A. Should Infants Be Separated from Mothers with COVID-19? First, DoNo Harm. Breastfeed Med. 2020 Apr 7. doi: 10.1089/bfm.2020.29153.ams. [Epub aheadof print] PubMed PMID: 32271625.	No original article
42: Huang X, Wei F, Hu L, Wen L, Chen K. Epidemiology and ClinicalCharacteristics of COVID-19. Arch Iran Med. 2020 Apr 1;23(4):268-271. doi:10.34172/aim.2020.09. Review. PubMed PMID: 32271601.	No original article
43: Bizzarri M, Laganà AS, Aragona D, Unfer V. Inositol and pulmonary function.Could myo-inositol treatment downregulate inflammation and cytokine releasesyndrome in SARS-CoV-2? Eur Rev Med Pharmacol Sci. 2020 Mar;24(6):3426-3432. doi:10.26355/eurrev_202003_20715. PubMed PMID: 32271462.	No appropriate outcome

44: Abu-Raya B. Predictors of refractory Coronavirus disease (COVID-19)pneumonia. Clin Infect Dis. 2020 Apr 9. pii: ciaa409. doi: 10.1093/cid/ciaa409.[Epub ahead of print] PubMed PMID: 32271373.	No appropriate outcome
45: Lighter J, Phillips M, Hochman S, Sterling S, Johnson D, Francois F, Stachel A. Obesity in patients younger than 60 years is a risk factor for Covid-19hospital admission. Clin Infect Dis. 2020 Apr 9. pii: ciaa415. doi:10.1093/cid/ciaa415. [Epub ahead of print] PubMed PMID: 32271368.	No appropriate population sample
46: Stoian AP, Banerjee Y, Rizvi AA, Rizzo M. Diabetes and the COVID-19 Pandemic:How Insights from Recent Experience Might Guide Future Management. Metab SyndrRelat Disord. 2020 Apr 8. doi: 10.1089/met.2020.0037. [Epub ahead of print]PubMed PMID: 32271125.	No appropriate population sample
47: Mohammadi S, Tabatabaei Yazdi SM, Eshghi P, Norooznezhad AH. Coronavirus-19Disease (COVID-19) and Decrease in Blood Donation: Experience of Iranian BloodTransfusion Organization (IBTO). Vox Sang. 2020 Apr 9. doi: 10.1111/vox.12930.[Epub ahead of print] PubMed PMID: 32270880.	No appropriate outcome
48: Lippi G, Henry BM, Sanchis-Gomar F. Physical inactivity and cardiovasculardisease at the time of coronavirus disease 2019 (COVID-19). Eur J Prev Cardiol.2020 Apr 9:2047487320916823. doi: 10.1177/2047487320916823. [Epub ahead of print]PubMed PMID: 32270698.	No appropriate outcome
49: Edelson DP, Sasson C, Chan PS, Atkins DL, Aziz K, Becker LB, Berg RA, BradleySM, Brooks SC, Cheng A, Escobedo M, Flores GE, Girotra S, Hsu A, Kamath-Rayne BD,Lee HC, Lehotzky RE, Mancini ME, Merchant RM, Nadkarni VM, Panchal AR, PeberdyMAR, Raymond TT, Walsh B, Wang DS, Zelop CM, Topjian A. Interim Guidance forBasic and Advanced Life Support in Adults, Children, and Neonates With Suspected or Confirmed COVID-19: From the Emergency	No original article

Cardiovascular Care Committee and GetWith the Guidelines(®)-Resuscitation Adult and Pediatric Task Forces of theAmerican Heart Association in Collaboration with the American Academy ofPediatrics, American Association for Respiratory Care, American College ofEmergency Physicians, The Society of Critical Care Anesthesiologists, andAmerican Society of Anesthesiologists: Supporting Organizations: AmericanAssociation of Critical Care Nurses and National EMS Physicians. Circulation.2020 Apr 9. doi: 10.1161/CIRCULATIONAHA.120.047463. [Epub ahead of print] PubMed PMID: 32270695.	
50: Zhu L, Wang J, Huang R, Liu L, Zhao H, Wu C, Zhu C. Clinical characteristics of a case series of children with coronavirus disease 2019. Pediatr Pulmonol.2020 Apr 8. doi: 10.1002/ppul.24767. [Epub ahead of print] PubMed PMID: 32270592.	No appropriate population sample
51: Kowalski LP, Sanabria A, Ridge JA, Ng WT, de Bree R, Rinaldo A, Takes RP,Mäkitie AA, Carvalho AL, Bradford CR, Paleri V, Hartl DM, Poorten VV, Nixon IJ,Piazza C, Lacy P, Rodrigo JP, Guntinas-Lichius O, Mendenhall WM, D'Cruz A, LeeAWM, Ferlito A. COVID-19 pandemic: effects and evidence-based recommendations forotolaryngology and head and neck surgery practice. Head Neck. 2020 Apr 9. doi:10.1002/hed.26164. [Epub ahead of print] PubMed PMID: 32270581.	No appropriate population sample
52: Wu X, Sun R, Chen J, Xie Y, Zhang S, Wang X. Radiological findings andclinical characteristics of pregnant women with COVID-19 pneumonia. Int JGynaecol Obstet. 2020 Apr 8. doi: 10.1002/ijgo.13165. [Epub ahead of print]PubMed PMID: 32270479.	Included
53: Ciavattini A, Delli Carpini G, Giannella L, De Vincenzo R, Frega A, CattaniP, Boselli F, Sopracordevole F, Barbero M. Expert consensus from the ItalianSociety for Colposcopy and Cervico-Vaginal Pathology (SICPCV) for colposcopy and outpatient surgery of the lower genital tract during the	No appropriate population sample

COVID-19 pandemic. Int JGynaecol Obstet. 2020 Apr 8. doi: 10.1002/ijgo.13158. [Epub ahead of print]PubMed PMID: 32270477.	
54: Salako O, Okunade K, Allsop M, Habeebu M, Toye M, Oluyede G, Fagbenro G,Salako B. Upheaval in cancer care during the COVID-19 outbreak.Ecancermedicalscience. 2020 Apr 1;14:ed97. doi: 10.3332/ecancer.2020.ed97.eCollection 2020. PubMed PMID: 32269597; PubMed Central PMCID: PMC7134578.	No appropriate population sample
55: Morray BH, Gordon BM, Crystal MA, Goldstein BH, Qureshi AM, Torres AJ,Epstein SM, Crittendon I, Ing FF, Sathanandam SK. Resource Allocation andDecision Making for Pediatric and Congenital Cardiac Catheterization During theNovel Coronavirus SARS-CoV-2 (COVID-19) Pandemic: A U.S. Multi-InstitutionalPerspective. J Invasive Cardiol. 2020 Apr 9. pii: JIC20200409-2. [Epub ahead ofprint] PubMed PMID: 32269177.	No appropriate population sample
56: Zhang ZJ, Yu XJ, Fu T, Liu Y, Jiang Y, Yang BX, Bi Y. Novel CoronavirusInfection in Newborn Babies Under 28 Days in China. Eur Respir J. 2020 Apr 8.pii: 2000697. doi: 10.1183/13993003.00697-2020. [Epub ahead of print] PubMedPMID: 32269087.	Included
57: Juurlink DN. Safety considerations with chloroquine, hydroxychloroquine andazithromycin in the management of SARS-CoV-2 infection. CMAJ. 2020 Apr 8. pii:cmaj.200528. doi: 10.1503/cmaj.200528. [Epub ahead of print] PubMed PMID:32269021	No appropriate outcome
58: Du L, Gu YB, Cui MQ, Li WX, Wang J, Zhu LP, Xu B. [Investigation on demandsfor antenatal care services among 2 002 pregnant women during the epidemic ofCOVID-19 in Shanghai]. Zhonghua Fu Chan Ke Za Zhi. 2020 Mar 25;55(3):160-165.doi: 10.3760/cma.j.cn112141-20200218-00112. Chinese. PubMed PMID: 32268713.	No appropriate outcome

59: Sun LL, Gong Q, Liao J, Ke JJ, Wang Y, Zhang YZ, Zhang W, Li JF.[Perioperative management of cesarean section for pregnant women with suspectedor confirmed COVID-19]. Zhonghua Fu Chan Ke Za Zhi. 2020 Mar 25;55(3):157-159.doi: 10.3760/cma.j.cn112141-20200220-00118. Chinese. PubMed PMID: 32268712.	No useful data
60: Pu J, Liu XX. [Systematic perinatal management of the pregnant women andneonates during the epidemic of COVID-19]. Zhonghua Fu Chan Ke Za Zhi. 2020 Mar25;55(3):153-156. doi: 10.3760/cma.j.cn112141-20200221-00123. Chinese. PubMedPMID: 32268711.	No original article
61: National Center for Health Care Quality Management in Obstetrics.[Suggestions on delivery management of pregnant women with COVID-19]. Zhonghua FuChan Ke Za Zhi. 2020 Mar 25;55(3):150-152. doi:10.3760/cma.j.cn112141-20200224-00128. Chinese. PubMed PMID: 32268710.	No original article
62: Wang C, Yang HX. [Attach importance to COVID-19 infection on maternal andinfant outcomes]. Zhonghua Fu Chan Ke Za Zhi. 2020 Mar 25;55(3):147-149. doi:10.3760/cma.j.cn112141-20200229-00151. Chinese. PubMed PMID: 32268709.	No original article
63: Yang C, Ma QY, Zheng YH, Yang YX. [Transmission routes of 2019-novelcoronavirus (2019-nCoV)]. Zhonghua Yu Fang Yi Xue Za Zhi. 2020 Apr6;54(4):374-377. doi: 10.3760/cma.j.cn112150-20200216-0016. Review. Chinese.PubMed PMID: 32268644.	No original article
64: Chen Y, Jin YL, Zhu LJ, Fang ZM, Wu N, Du MX, Jiang MM, Wang J, Yao YS. [The network investigation on knowledge, attitude and practice about COVID-19 of theresidents in Anhui Province]. Zhonghua Yu Fang Yi Xue Za Zhi. 2020 Apr6;54(4):367-373. doi: 10.3760/cma.j.cn112150-20200205-00069. Chinese. PubMedPMID: 32268643.	No appropriate outcome

65: Grange ES, Neil EJ, Stoffel M, Singh AP, Tseng E, Resco-Summers K, FellnerBJ, Lynch JB, Mathias PC, Mauritz-Miller K, Sutton PR, Leu MG. Responding toCOVID-19: The UW Medicine Information Technology Services Experience. Appl ClinInform. 2020 Mar;11(2):265-275. doi: 10.1055/s-0040-1709715. Epub 2020 Apr 8.PubMed PMID: 32268390.	No appropriate outcome
66: Chandrasekharan P, Vento M, Trevisanuto D, Partridge E, Underwood MA,Wiedeman J, Katheria A, Lakshminrusimha S. Neonatal Resuscitation andPostresuscitation Care of Infants Born to Mothers with Suspected or ConfirmedSARS-CoV-2 Infection. Am J Perinatol. 2020 Apr 8. doi: 10.1055/s-0040-1709688.[Epub ahead of print] PubMed PMID: 32268381.	No original article
67: Zhu L, Lu X, Chen L. Possible causes for decreased susceptibility of childrento coronavirus. Pediatr Res. 2020 Apr 8. doi: 10.1038/s41390-020-0892-8. [Epubahead of print] PubMed PMID: 32268343.68: Wang KW, Gao J, Wang H, Wu XL, Yuan QF, Guo FY, Zhang ZJ, Cheng Y.	No original article
68: Wang KW, Gao J, Wang H, Wu XL, Yuan QF, Guo FY, Zhang ZJ, Cheng Y.Epidemiology of 2019 novel coronavirus in Jiangsu Province, China after wartimecontrol measures: A population-level retrospective study. Travel Med Infect Dis. 2020 Apr 5:101654. doi: 10.1016/j.tmaid.2020.101654. [Epub ahead of print] PubMedPMID: 32268195; PubMed Central PMCID: PMC7130124.	No appropriate outcome
69: Molloy EJ, Murphy N. Vitamin D, Covid-19 and Children. Ir Med J. 2020 Apr3;113(4):64. PubMed PMID: 32268052.	No appropriate population sample

70: O'Brien M, Moore K, McNicholas F. Social Media Spread During Covid-19: ThePros and Cons of Likes and Shares. Ir Med J. 2020 Apr 3;113(4):52. PubMed PMID:32268046	No appropriate outcome
71: Gavin B, Hayden J, Adamis D, McNicholas F. Caring for the PsychologicalWell-Being of Healthcare Professionals in the Covid-19 Pandemic Crisis. Ir Med J.2020 Apr 3;113(4):51. PubMed PMID: 32268045.	No appropriate outcome
72: Wang J, Hajizadeh N, Moore EE, McIntyre RC, Moore PK, Veress LA, Yaffe MB,Moore HB, Barrett CD. Tissue Plasminogen Activator (tPA) Treatment for COVID-19Associated Acute Respiratory Distress Syndrome (ARDS): A Case Series. J ThrombHaemost. 2020 Apr 8. doi: 10.1111/jth.14828. [Epub ahead of print] PubMed PMID:32267998	No appropriate population sample
73: Bourne T, Leonardi M, Kyriacou C, Al-Memar M, Landolfo C, Cibula D, CondousG, Metzger U, Fischerova D, Timmerman D, van den Bosch T. ISUOG ConsensusStatement on rationalization of gynecological ultrasound services in context ofSARS-CoV-2. Ultrasound Obstet Gynecol. 2020 Apr 8. doi: 10.1002/uog.22047. [Epub ahead of print] PubMed PMID: 32267984.	No original article
74: Bourne T, Kyriacou C, Coomarasamy A, Al-Memar M, Leonardi M, Kirk E, LandolfoC, Blanchette-Porter M, Small R, Condous G, Timmerman D. ISUOG ConsensusStatement on rationalization of early-pregnancy care and provision ofultrasonography in context of SARS-CoV-2. Ultrasound Obstet Gynecol. 2020 Apr 8. doi: 10.1002/uog.22046. [Epub ahead of print] PubMed PMID: 32267981.	No original article
75: Huang H, Fan C, Li M, Nie HL, Wang FB, Wang H, Wang R, Xia J, Zheng X, Zuo X,Huang J. COVID-19: A Call for Physical Scientists and Engineers. ACS Nano. 2020Apr 8. doi: 10.1021/acsnano.0c02618. [Epub ahead of print] PubMed PMID: 32267678;PubMed Central PMCID: PMC7144807.	No appropriate population sample

76: Mahony R. Pregnancy and Sars-Cov-2: A Novel Virus in a Unique Population. Ir Med J. 2020 Apr 3;113(4):49. PubMed PMID: 32267662.	No original article
77: Ferrazzi EM, Frigerio L, Cetin I, Vergani P, Spinillo A, Prefumo F,Pellegrini E, Gargantini G. COVID-19 Obstetrics Task Force, Lombardy, Italy:executive management summary and short report of outcome. Int J Gynaecol Obstet. 2020 Apr 8. doi: 10.1002/ijgo.13162. [Epub ahead of print] PubMed PMID: 32267531.	No original article
78: Tagarro A, Epalza C, Santos M, Sanz-Santaeufemia FJ, Otheo E, Moraleda C,Calvo C. Screening and Severity of Coronavirus Disease 2019 (COVID-19) inChildren in Madrid, Spain. JAMA Pediatr. 2020 Apr 8. doi:10.1001/jamapediatrics.2020.1346. [Epub ahead of print] PubMed PMID: 32267485.	No appropriate population sample
79: Lippi G, Henry BM, Bovo C, Sanchis-Gomar F. Health risks and potentialremedies during prolonged lockdowns for coronavirus disease 2019 (COVID-19).Diagnosis (Berl). 2020 Apr 7. pii:/j/dx.ahead-of-print/dx-2020-0041/dx-2020-0041.xml. doi: 10.1515/dx-2020-0041.[Epub ahead of print] PubMed PMID: 32267243.	No appropriate outcome
80: Davenne E, Giot JB, Huynen P. [Coronavirus and COVID-19 : focus on agalopping pandemic]. Rev Med Liege. 2020 Apr;75(4):218-225. French. PubMed PMID: 3226710981: Thampi S, Yap A, Lijia F, Ong J. Special considerations for the management of	No original article
81: Thampi S, Yap A, Lijia F, Ong J. Special considerations for the management ofCOVID-19 pediatric patients in the operating room and pediatric intensive careunit in a tertiary hospital in Singapore. Paediatr Anaesth. 2020 Apr 8. doi:10.1111/pan.13863. [Epub ahead of print] PubMed PMID: 32267047.	No original article

82: Lippi G, South AM, Henry BM. ANNALS EXPRESS: Electrolyte Imbalances inPatients with Severe Coronavirus Disease 2019 (COVID-19). Ann Clin Biochem. 2020 Apr 8:4563220922255. doi: 10.1177/0004563220922255. [Epub ahead of print] PubMed PMID: 32266828.	No appropriate population sample
83: Wang C, Zhou YH, Yang HX, Poon LC. Intrauterine vertical transmission ofSARS-CoV-2: what we know so far. Ultrasound Obstet Gynecol. 2020 Apr 7. doi:10.1002/uog.22045. [Epub ahead of print] PubMed PMID: 32266753.84: Iaccarino G, Borghi C, Cicero AFG, Ferri C, Minuz P, Muiesan ML, Mulatero P,	No original article
84: Iaccarino G, Borghi C, Cicero AFG, Ferri C, Minuz P, Muiesan ML, Mulatero P, Mulè G, Pucci G, Salvetti M, Savoia C, Sechi LA, Volpe M, Grassi G.Renin-Angiotensin System Inhibition in Cardiovascular Patients at the Time ofCOVID19: Much Ado for Nothing? A Statement of Activity from the Directors of the Board and the Scientific Directors of the Italian Society of Hypertension. HighBlood Press Cardiovasc Prev. 2020 Apr 7. doi: 10.1007/s40292-020-00380-3. [Epub	No appropriate population sample
85: Chen Y, Peng H, Wang L, Zhao Y, Zeng L, Gao H, Liu Y. Infants Born to MothersWith a New Coronavirus (COVID-19). Front Pediatr. 2020 Mar 16;8:104. doi:10.3389/fped.2020.00104. eCollection 2020. PubMed PMID: 32266184; PubMed Central PMCID: PMC7098456.	Overlapping data
86: Bauer M, Bernstein K, Dinges E, Delgado C, El-Sharawi N, Sultan P, Mhyre JM, Landau R. Obstetric Anesthesia During the COVID-19 Pandemic. Anesth Analg. 2020Apr 6. doi: 10.1213/ANE.0000000000004856. [Epub ahead of print] PubMed PMID:32265365	No original article
87: Li K, Li Z, Wohlford-Lenane C, Meyerholz DK, Channappanavar R, An D, Perlman S, McCray PB Jr, He B. Single-Dose, Intranasal Immunization with RecombinantParainfluenza Virus 5 Expressing Middle East Respiratory	No appropriate outcome

Syndrome Coronavirus(MERS-CoV) Spike Protein Protects Mice from Fatal MERS-CoV Infection. mBio. 2020 Apr 7;11(2). pii: e00554-20. doi: 10.1128/mBio.00554-20. PubMed PMID: 32265331.	
88: Swennen GRJ, Pottel L, Haers PE. Custom-made 3D-printed face masks in case ofpandemic crisis situations with a lack of commercially available FFP2/3 masks.Int J Oral Maxillofac Surg. 2020 Apr 2. pii: S0901-5027(20)30123-5. doi:10.1016/j.ijom.2020.03.015. [Epub ahead of print] PubMed PMID: 32265088.	No appropriate outcome
89: Penel N, Bonvalot S, Minard V, Orbach D, Gouin F, Corradini N, Brahmi M,Marec-Berard P, Briand S, Gaspar N, Llacer C, Carrere S, Dufresne A, Le Cesne A, Blay JY. [French Sarcoma Group proposals for management of sarcoma patientsduring COVID-19 outbreak]. Bull Cancer. 2020 Apr 1. pii: S0007-4551(20)30160-0.doi: 10.1016/j.bulcan.2020.03.009. [Epub ahead of print] French. PubMed PMID:32265035; PubMed Central PMCID: PMC7118687.	No appropriate population sample
90: COVID-19 National Incident Room Surveillance Team. COVID-19, Australia:Epidemiology Report 9 (Reporting week to 23:59 AEDT 29 March 2020). Commun DisIntell (2018). 2020 Apr 3;44. doi: 10.33321/cdi.2020.44.29. PubMed PMID:32264802	No appropriate outcome
91: Haşlak F, Yıldız M, Adrovic A, Barut K, Kasapçopur Ö. Childhood RheumaticDiseases and COVID-19 Pandemic: An Intriguing Linkage and a New Horizon. BalkanMed J. 2020 Apr 8. doi: 10.4274/balkanmedj.galenos.2020.2020.4.43. [Epub ahead ofprint] PubMed PMID: 32264666.	No appropriate outcome
92: Bansal P, Bingemann TA, Greenhawt M, Mosnaim G, Nanda A, Oppenheimer J,Sharma H, Stukus D, Shaker M. Clinician Wellness During the COVID-19 Pandemic:Extraordinary Times and Unusual Challenges for the Allergist/Immunologist.	No appropriate population sample

JAllergy Clin Immunol Pract. 2020 Apr 4. pii: S2213-2198(20)30327-5. doi:10.1016/j.jaip.2020.04.001. [Epub ahead of print] Review. PubMed PMID: 32259628; PubMed Central PMCID: PMC7129776.	
93: Kim YI, Kim SG, Kim SM, Kim EH, Park SJ, Yu KM, Chang JH, Kim EJ, Lee S,Casel MAB, Um J, Song MS, Jeong HW, Lai VD, Kim Y, Chin BS, Park JS, Chung KH,Foo SS, Poo H, Mo IP, Lee OJ, Webby RJ, Jung JU, Choi YK. Infection and RapidTransmission of SARS-CoV-2 in Ferrets. Cell Host Microbe. 2020 Apr 5. pii:S1931-3128(20)30187-6. doi: 10.1016/j.chom.2020.03.023. [Epub ahead of print]PubMed PMID: 32259477; PubMed Central PMCID: PMC7144857.	No appropriate population sample
94: Shen Q, Guo W, Guo T, Li J, He W, Ni S, Ouyang X, Liu J, Xie Y, Tan X, ZhouZ, Peng H. Novel coronavirus infection in children outside of Wuhan, China.Pediatr Pulmonol. 2020 Apr 7. doi: 10.1002/ppul.24762. [Epub ahead of print]PubMed PMID: 32259403.	No appropriate population sample
95: Chen CC, Chi CY. Biosafety in the preparation and processing of cytologyspecimens with potential coronavirus (COVID-19) infection: Perspectives fromTaiwan. Cancer Cytopathol. 2020 Apr 7. doi: 10.1002/cncy.22280. [Epub ahead ofprint] PubMed PMID: 32259402.	No appropriate outcome
96: Yang S, Zhang Y, Cai J, Wang Z. Clinical Characteristics of COVID-19 After Gynecologic Oncology Surgery in Three Women: A Retrospective Review of MedicalRecords. Oncologist. 2020 Apr 7. doi: 10.1634/theoncologist.2020-0157. [Epubahead of print] PubMed PMID: 32259322.	No appropriate population sample
97: Zaigham M, Andersson O. Maternal and Perinatal Outcomes with COVID-19: a systematic review of 108 pregnancies. Acta Obstet Gynecol Scand. 2020 Apr 7. doi:10.1111/aogs.13867. [Epub ahead of print] Review. PubMed PMID: 32259279.	No original article

98: Boccia S, Ricciardi W, Ioannidis JPA. What Other Countries Can Learn FromItaly During the COVID-19 Pandemic. JAMA Intern Med. 2020 Apr 7. doi:10.1001/jamainternmed.2020.1447. [Epub ahead of print] PubMed PMID: 32259190.	No appropriate outcome
99: Mallick R, Odejinmi F, Clark TJ. Covid 19 pandemic and gynaecological laparoscopic surgery: knowns and unknowns. Facts Views Vis Obgyn. 2020 Apr1;12(1):3-7. PubMed PMID: 32259155; PubMed Central PMCID: PMC7117791.	No appropriate outcome
100: Gharebaghi R, Heidary F, Moradi M, Parvizi M. Metronidazole; a PotentialNovel Addition to the COVID-19 Treatment Regimen. Arch Acad Emerg Med. 2020 Mar30;8(1):e40. eCollection 2020. PubMed PMID: 32259129; PubMed Central PMCID:PMC7114714.	No appropriate outcome
101: Gaeta C, Brennessel R. COVID-19: Emergency Medicine Physician Empowered toShape Perspectives on This Public Health Crisis. Cureus. 2020 Apr 1;12(4):e7504. doi: 10.7759/cureus.7504. PubMed PMID: 32257730; PubMed Central PMCID:PMC7117614.	No appropriate outcome
102: Hasan A, Mehmood N, Fergie J. Coronavirus Disease (COVID-19) and PediatricPatients: A Review of Epidemiology, Symptomatology, Laboratory and ImagingResults to Guide the Development of a Management Algorithm. Cureus. 2020 Mar31;12(3):e7485. doi: 10.7759/cureus.7485. Review. PubMed PMID: 32257728; PubMedCentral PMCID: PMC7123290.	No original article
103: Leng Z, Zhu R, Hou W, Feng Y, Yang Y, Han Q, Shan G, Meng F, Du D, Wang S,Fan J, Wang W, Deng L, Shi H, Li H, Hu Z, Zhang F, Gao J, Liu H, Li X, Zhao Y,Yin K, He X, Gao Z, Wang Y, Yang B, Jin R, Stambler I, Lim LW, Su H, Moskalev A, Cano A, Chakrabarti S, Min KJ, Ellison-Hughes G, Caruso C, Jin K, Zhao RC.Transplantation of ACE2(-) Mesenchymal Stem Cells Improves the Outcome ofPatients with COVID-19	No appropriate population sample

Pneumonia. Aging Dis. 2020 Mar 9;11(2):216-228. doi:10.14336/AD.2020.0228. eCollection 2020 Apr. PubMed PMID: 32257537; PubMedCentral PMCID: PMC7069465.	
104: Asim M, Sathian B, van Teijlingen E, Mekkodathil A, Subramanya SH, Simkhada P. COVID-19 Pandemic: Public Health Implications in Nepal. Nepal J Epidemiol.2020 Mar 30;10(1):817-820. doi: 10.3126/nje.v10i1.28269. eCollection 2020 Mar.PubMed PMID: 32257511; PubMed Central PMCID: PMC7112957.	No appropriate outcome
105: Chan PS, Berg RA, Nadkarni VM. Code Blue During the COVID-19 Pandemic. Circ Cardiovasc Qual Outcomes. 2020 Apr 7. doi: 10.1161/CIRCOUTCOMES.120.006779. [Epubahead of print] PubMed PMID: 32255661.	No appropriate outcome
106: Abramowicz JS, Basseal JM, Brezinka C, Dall'Asta A, Deng J, Harrison G, Lee JCS, Lim A, Maršal K, Miloro P, Poon LC, Salvesen KÅ, Sande R, Ter Haar G,Westerway SC, Xie MX, Lees C. ISUOG Safety Committee Position Statement on use of personal protective equipment and hazard mitigation in relation to SARS-CoV-2 forpractitioners undertaking obstetric and gynecological ultrasound. UltrasoundObstet Gynecol. 2020 Apr 7. doi: 10.1002/uog.22035. [Epub ahead of print] PubMed PMID: 32255535.	No appropriate outcome
107: Li B, Shen J, Li L, Yu C. Radiographic and Clinical Features of Childrenwith 2019 Novel Coronavirus (COVID-19) Pneumonia. Indian Pediatr. 2020 Apr 7.pii: S097475591600156. [Epub ahead of print] PubMed PMID: 32255437.	No appropriate population sample
108: Yadav U, Pal R. Challenging Times for Children With Transfusion-dependent Thalassemia Amid the COVID-19 Pandemic. Indian Pediatr. 2020 Apr 7. pii:S097475591600155. [Epub ahead of print] PubMed PMID: 32255436.	No appropriate population sample

109: Rubin GD, Ryerson CJ, Haramati LB, Sverzellati N, Kanne JP, Raoof S,Schluger NW, Volpi A, Yim JJ, Martin IBK, Anderson DJ, Kong C, Altes T, Bush A,Desai SR, Goldin J, Goo JM, Humbert M, Inoue Y, Kauczor HU, Luo F, Mazzone PJ,Prokop M, Remy-Jardin M, Richeldi L, Schaefer-Prokop CM, Tomiyama N, Wells AU,Leung AN. The Role of Chest Imaging in Patient Management during the COVID-19Pandemic: A Multinational Consensus Statement from the Fleischner Society.Radiology. 2020 Apr 7:201365. doi: 10.1148/radiol.2020201365. [Epub ahead ofprint] PubMed PMID: 32255413.	No appropriate outcome
110: Mo Y, Deng L, Zhang L, Lang Q, Liao C, Wang N, Qin M, Huang H. Work stress among Chinese nurses to support Wuhan for fighting against the COVID-19 epidemic.J Nurs Manag. 2020 Apr 7. doi: 10.1111/jonm.13014. [Epub ahead of print] PubMedPMID: 32255222.	No appropriate outcome
111: Ji T, Chen HL, Xu J, Wu LN, Li JJ, Chen K, Qin G. Lockdown contained thespread of 2019 novel coronavirus disease in Huangshi city, China: Earlyepidemiological findings. Clin Infect Dis. 2020 Apr 7. pii: ciaa390. doi:10.1093/cid/ciaa390. [Epub ahead of print] PubMed PMID: 32255183.	No appropriate outcome
112: Bloch EM, Shoham S, Casadevall A, Sachais BS, Shaz B, Winters JL, vanBuskirk C, Grossman BJ, Joyner M, Henderson JP, Pekosz A, Lau B, Wesolowski A,Katz L, Shan H, Auwaerter PG, Thomas D, Sullivan DJ, Paneth N, Gehrie E,Spitalnik S, Hod E, Pollack L, Nicholson WT, Pirofski LA, Bailey JA, Tobian AA.Deployment of convalescent plasma for the prevention and treatment of COVID-19. JClin Invest. 2020 Apr 7. pii: 138745. doi: 10.1172/JCI138745. [Epub ahead ofprint] Review. PubMed PMID: 32254064.	No appropriate outcome

113: Sheahan TP, Sims AC, Zhou S, Graham RL, Pruijssers AJ, Agostini ML, LeistSR, Schäfer A, Dinnon KH 3rd, Stevens LJ, Chappell JD, Lu X, Hughes TM, GeorgeAS, Hill CS, Montgomery SA, Brown AJ, Bluemling GR, Natchus MG, Saindane M,Kolykhalov AA, Painter G, Harcourt J, Tamin A, Thornburg NJ, Swanstrom R, DenisonMR, Baric RS. An orally bioavailable broad-spectrum antiviral inhibits SARS-CoV-2in human airway epithelial cell cultures and multiple coronaviruses in mice. Sci Transl Med. 2020 Apr 6. pii: eabb5883. doi: 10.1126/scitranslmed.abb5883. [Epubahead of print] PubMed PMID: 32253226.	No appropriate outcome
114: Davanzo R. Breast feeding at the time of COVID-19: do not forget expressed mother's milk, please. Arch Dis Child Fetal Neonatal Ed. 2020 Apr 6. pii:fetalneonatal-2020-319149. doi: 10.1136/archdischild-2020-319149. [Epub ahead of print] PubMed PMID: 32253201.	No original article
115: Morand A, Fabre A, Minodier P, Boutin A, Vanel N, Bosdure E, Fournier PE.COVID-19 virus and children: What do we know? Arch Pediatr. 2020Apr;27(3):117-118. doi: 10.1016/j.arcped.2020.03.001. PubMed PMID: 32253003.	No original article
116: Park SE. Epidemiology, virology, and clinical features of severe acuterespiratory syndrome -coronavirus-2 (SARS-CoV-2; Coronavirus Disease-19). ClinExp Pediatr. 2020 Apr 2. doi: 10.3345/cep.2020.00493. [Epub ahead of print]PubMed PMID: 32252141.	No appropriate outcome
117: Choi SH, Kim HW, Kang JM, Kim DH, Cho EY. Epidemiology and Clinical Featuresof Coronavirus disease 2019 in Children. Clin Exp Pediatr. 2020 Apr 6. doi:10.3345/cep.2020.00535. [Epub ahead of print] PubMed PMID: 32252139.	No original article
118: Hong X, Xiong J, Feng Z, Shi Y. Extracorporeal membrane oxygenation (ECMO): does it have a role in the treatment of severe COVID-19? Int J Infect Dis. 2020Apr 3. pii: S1201-9712(20)30191-0. doi:	No appropriate outcome

10.1016/j.ijid.2020.03.058. [Epub aheadof print] PubMed PMID: 32251794.	
119: Gray PE, Belessis Y. The use of Traditional Chinese Medicines to treatSARS-CoV-2 may cause more harm than good. Pharmacol Res. 2020 Apr 3:104776. doi: 10.1016/j.phrs.2020.104776. [Epub ahead of print] PubMed PMID: 32251726; PubMedCentral PMCID: PMC7129235.	No appropriate outcome
120: Weber Lebrun EE, Moawad NS, Rosenberg EI, Morey TE, Davies L, Collins WO,Smulian JC. COVID-19 Pandemic: Staged Management of Surgical Services forGynecology and Obstetrics. Am J Obstet Gynecol. 2020 Apr 3. pii:S0002-9378(20)30389-6. doi: 10.1016/j.ajog.2020.03.038. [Epub ahead of print]PubMed PMID: 32251649.	No appropriate outcome
121: Favalli EG, Biggioggero M, Maioli G, Caporali R. Baricitinib for COVID-19: asuitable treatment? Lancet Infect Dis. 2020 Apr 3. pii: S1473-3099(20)30262-0.doi: 10.1016/S1473-3099(20)30262-0. [Epub ahead of print] PubMed PMID: 32251638.	No appropriate outcome
122: Bayham J, Fenichel EP. Impact of school closures for COVID-19 on the UShealth-care workforce and net mortality: a modelling study. Lancet Public Health.2020 Apr 3. pii: S2468-2667(20)30082-7. doi: 10.1016/S2468-2667(20)30082-7. [Epubahead of print] PubMed PMID: 32251626.	No appropriate outcome
123: Zamberg I, Manzano S, Posfay-Barbe K, Windisch O, Agoritsas T, Schiffer E.Utilization of a mobile platform for the dissemination of validated institutionalmeasurements during CoVid-19 Outbreak: A practical example in the Children'sHospital. JMIR Public Health Surveill. 2020 Apr 3. doi: 10.2196/18668. [Epubahead of print] PubMed PMID: 32250958.	No appropriate outcome

124: Mallineni SK, Innes NP, Raggio DP, Araujo MP, Robertson MD, Jayaraman J.Coronavirus Disease (COVID-19): Characteristics in children and considerationsfor Dentists providing their care. Int J Paediatr Dent. 2020 Apr 6. doi:10.1111/ipd.12653. [Epub ahead of print] PubMed PMID: 32250505.	No appropriate outcome
125: Kaufman KR, Petkova E, Bhui KS, Schulze TG. A global needs assessment intimes of a global crisis: world psychiatry response to the COVID-19 pandemic.BJPsych Open. 2020 Apr 6:1-11. doi: 10.1192/bjo.2020.25. [Epub ahead of print]PubMed PMID: 32250235.	No appropriate outcome
126: Joob B, Wiwanitkit V. Hemorrhagic Problem Among the Patients With COVID-19: Clinical Summary of 41 Thai Infected Patients. Clin Appl Thromb Hemost. 2020Jan-Dec;26:1076029620918308. doi: 10.1177/1076029620918308. PubMed PMID:32250159	No appropriate outcome
127: Canarutto D, Priolo A, Russo G, Pitea M, Vigone MC, Barera G. COVID-19infection in a paucisymptomatic infant: Raising the index of suspicion inepidemic settings. Pediatr Pulmonol. 2020 Apr 6. doi: 10.1002/ppul.24754. [Epubahead of print] PubMed PMID: 32250049.	No appropriate population sample
128: Han YN, Feng ZW, Sun LN, Ren XX, Wang H, Xue YM, Wang Y, Fang Y. A comparative-descriptive analysis of clinical characteristics in2019-Coronavirus-infected children and adults. J Med Virol. 2020 Apr 6. doi:10.1002/jmv.25835. [Epub ahead of print] PubMed PMID: 32249943.	No appropriate population sample
129: Gidlöf S, Savchenko J, Brune T, Josefsson H. COVID-19 in pregnancy withcomorbidities: More liberal testing strategy is needed. Acta Obstet GynecolScand. 2020 Apr 6. doi: 10.1111/aogs.13862. [Epub ahead of print] PubMed PMID:32249924	Included

130: Li N, Han L, Peng M, Lv Y, Ouyang Y, Liu K, Yue L, Li Q, Sun G, Chen L, YangL. Maternal and neonatal outcomes of pregnant women with COVID-19 pneumonia: acase-control study. Clin Infect Dis. 2020 Mar 30. pii: ciaa352. doi:10.1093/cid/ciaa352. [Epub ahead of print] PubMed PMID: 32249918.	Overlapping data
131: Kalafat E, Yaprak E, Cinar G, Varli B, Ozisik S, Uzun C, Azap A, Koc A. Lung ultrasound and computed tomographic findings in pregnant woman with COVID-19.Ultrasound Obstet Gynecol. 2020 Apr 6. doi: 10.1002/uog.22034. [Epub ahead ofprint] PubMed PMID: 32249471.	Included
132: Rey Galán C, Manrique de Lara LA, Antón Gamero M, Cano Garcinuño A, SolísSánchez G. [Coronavirus infection (COVID-19) in Anales de Pediatría]. An Pediatr (Barc). 2020 Apr 2. pii: S1695-4033(20)30132-6. doi:10.1016/j.anpedi.2020.03.004. [Epub ahead of print] Spanish. PubMed PMID:32249164; PubMed Central PMCID: PMC7128409.	No original article
133: Jiao WY, Wang LN, Liu J, Fang SF, Jiao FY, Pettoello-Mantovani M, Somekh E. Behavioral and Emotional Disorders in Children during the COVID-19 Epidemic. JPediatr. 2020 Apr 2. pii: S0022-3476(20)30336-X. doi:10.1016/j.jpeds.2020.03.013. [Epub ahead of print] PubMed PMID: 32248989; PubMed Central PMCID: PMC7127630.	No appropriate outcome
134: Sahu KK, Mishra AK, Lal A. A twin challenge to handle: COVID-19 withpregnancy. J Med Virol. 2020 Apr 5. doi: 10.1002/jmv.25784. [Epub ahead of print]PubMed PMID: 32248565.	No original article
135: Poon LC, Yang H, Kapur A, Melamed N, Dao B, Divakar H, David McIntyre H,Kihara AB, Ayres-de-Campos D, Ferrazzi EM, Carlo Di Renzo G, Hod M. Global interim guidance on coronavirus disease 2019 (COVID-19) during pregnancy andpuerperium from FIGO and allied partners: Information for healthcareprofessionals. Int J Gynaecol Obstet. 2020 Apr 4. doi: 10.1002/ijgo.13156.	No original article

[Epub ahead of print] PubMed PMID: 32248521.	
136: Grech V. Unknown unknowns - COVID-19 and potential global mortality. EarlyHum Dev. 2020 Mar 31;144:105026. doi: 10.1016/j.earlhumdev.2020.105026. [Epubahead of print] PubMed PMID: 32247898.	No appropriate outcome
137: Morris SN, Fader AN, Milad MP, Dionisi HJ. Understanding the "Scope" of the Problem: Why Laparoscopy is Considered Safe During the COVID-19 Pandemic. J MinimInvasive Gynecol. 2020 Apr 2. pii: S1553-4650(20)30171-0. doi:10.1016/j.jmig.2020.04.002. [Epub ahead of print] PubMed PMID: 32247882; PubMedCentral PMCID: PMC7129473.	No appropriate outcome
138: Norsa L, Indriolo A, Sansotta N, Cosimo P, Greco S, D'Antiga L. Uneventful course in IBD patients during SARS-CoV-2 outbreak in northern Italy.Gastroenterology. 2020 Apr 2. pii: S0016-5085(20)30445-5. doi:10.1053/j.gastro.2020.03.062. [Epub ahead of print] PubMed PMID: 32247695.	No appropriate population sample
139: Akladios C, Azais H, Ballester M, Bendifallah S, Bolze PA, Bourdel N, BricouA, Canlorbe G, Carcopino X, Chauvet P, Collinet P, Coutant C, Dabi Y, Dion L,Gauthier T, Graesslin O, Huchon C, Koskas M, Kridelka F, Lavoue V, Lecointre L,Mezzadri M, Mimoun C, Ouldamer L, Raimond E, Touboul C. Recommendations for thesurgical management of gynecological cancers during the COVID-19 pandemic -FRANCOGYN group for the CNGOF. J Gynecol Obstet Hum Reprod. 2020 Apr 1:101729.doi: 10.1016/j.jogoh.2020.101729. [Epub ahead of print] PubMed PMID: 32247066;PubMed Central PMCID: PMC7118621.	No appropriate population sample

140: Al-Tawfiq JA. Middle East Respiratory Syndrome Coronavirus (MERS-CoV) andCOVID-19 infection during pregnancy. Travel Med Infect Dis. 2020 Apr 1:101641.doi: 10.1016/j.tmaid.2020.101641. [Epub ahead of print] PubMed PMID: 32247017;PubMed Central PMCID: PMC7118624.	No original article
141: Baker T, Schell CO, Petersen DB, Sawe H, Khalid K, Mndolo S, Rylance J,McAuley DF, Roy N, Marshall J, Wallis L, Molyneux E. Essential care of critical illness must not be forgotten in the COVID-19 pandemic. Lancet. 2020 Apr 1. pii: S0140-6736(20)30793-5. doi: 10.1016/S0140-6736(20)30793-5. [Epub ahead of print] PubMed PMID: 32246914.	No appropriate outcome
142: He Y, Lin Z, Tang D, Yang Y, Wang T, Yang M. Strategic plan for managementof COVID-19 in paediatric haematology and oncology departments. Lancet Haematol. 2020 Apr 1. pii: S2352-3026(20)30104-6. doi: 10.1016/S2352-3026(20)30104-6. [Epubahead of print] PubMed PMID: 32246912.	No appropriate outcome
143: Furlow B. US NICUs and donor milk banks brace for COVID-19. Lancet ChildAdolesc Health. 2020 Apr 1. pii: S2352-4642(20)30103-6. doi:10.1016/S2352-4642(20)30103-6. [Epub ahead of print] PubMed PMID: 32246910.	No appropriate outcome
144: Altena E, Baglioni C, Espie CA, Ellis J, Gavriloff D, Holzinger B, SchlarbA, Frase L, Jernelöv S, Riemann D. Dealing with sleep problems during homeconfinement due to the COVID-19 outbreak: practical recommendations from a taskforce of the European CBT-I Academy. J Sleep Res. 2020 Apr 4. doi:10.1111/jsr.13052. [Epub ahead of print] PubMed PMID: 32246787.	No appropriate outcome
145: Nagata JM. Rapid Scale-Up of Telehealth during the COVID-19 Pandemic and Implications for Subspecialty Care in Rural Areas. J Rural Health. 2020 Apr 3.doi: 10.1111/jrh.12433. [Epub ahead of print] PubMed PMID: 32246490.	No appropriate outcome

146: Matava CT, Yu J, Denning S. Clear plastic drapes may be effective atlimiting aerosolization and droplet spray during extubation: implications forCOVID-19. Can J Anaesth. 2020 Apr 3. doi: 10.1007/s12630-020-01649-w. [Epub aheadof print] PubMed PMID: 32246431; PubMed Central PMCID: PMC7124129.	No appropriate outcome
147: South AM, Tomlinson L, Edmonston D, Hiremath S, Sparks MA. Controversies of renin-angiotensin system inhibition during the COVID-19 pandemic. Nat RevNephrol. 2020 Apr 3. doi: 10.1038/s41581-020-0279-4. [Epub ahead of print] PubMedPMID: 32246101.	No appropriate outcome
148: Henrickson SE. Learning from our immunological history: What can SARS-CoVteach us about SARS-CoV-2? Sci Immunol. 2020 Apr 3;5(46). pii: eabb8618. doi:10.1126/sciimmunol.abb8618. PubMed PMID: 32245885.	No appropriate outcome
149: Kickbusch I, Leung GM, Bhutta ZA, Matsoso MP, Ihekweazu C, Abbasi K.Covid-19: how a virus is turning the world upside down. BMJ. 2020 Apr3;369:m1336. doi: 10.1136/bmj.m1336. PubMed PMID: 32245802.	No appropriate outcome
150: Gardner W, States D, Bagley N. The Coronavirus and the Risks to the Elderly in Long-Term Care. J Aging Soc Policy. 2020 Apr 3:1-6. doi:10.1080/08959420.2020.1750543. [Epub ahead of print] PubMed PMID: 32245346.	No appropriate population sample
151: Narzisi A. Handle the Autism Spectrum Condition During Coronavirus(COVID-19) Stay At Home period: Ten Tips for Helping Parents and Caregivers ofYoung Children. Brain Sci. 2020 Apr 1;10(4). pii: E207. doi:10.3390/brainsci10040207. PubMed PMID: 32244776.	No appropriate outcome

181: CDC COVID-19 Response Team. Preliminary Estimates of the Prevalence ofSelected Underlying Health Conditions Among Patients with Coronavirus Disease2019 - United States, February 12-March 28, 2020. MMWR Morb Mortal Wkly Rep. 2020Apr 3;69(13):382-386. doi: 10.15585/mmwr.mm6913e2. PubMed PMID: 32240123; PubMed Central PMCID: PMC7119513.	No appropriate outcome
191: Eduardo C. [The novel coronavirus covid-19 pandemic]. Rev Fac Cien Med Univ Nac Cordoba. 2020 Mar 18;77(1):1-3. doi: 10.31053/1853.0605.v77.n1.27935.Spanish. PubMed PMID: 32238250.	No original article
203: Russell TW, Hellewell J, Jarvis CI, van Zandvoort K, Abbott S, Ratnayake R, Cmmid Covid-Working Group, Flasche S, Eggo RM, Edmunds WJ, Kucharski AJ.Estimating the infection and case fatality ratio for coronavirus disease(COVID-19) using age-adjusted data from the outbreak on the Diamond Princesscruise ship, February 2020. Euro Surveill. 2020 Mar;25(12). doi:10.2807/1560-7917.ES.2020.25.12.2000256. PubMed PMID: 32234121; PubMed CentralPMCID: PMC7118348.	No appropriate population sample
240: Udugama B, Kadhiresan P, Kozlowski HN, Malekjahani A, Osborne M, Li VYC,Chen H, Mubareka S, Gubbay JB, Chan WCW. Diagnosing COVID-19: The Disease andTools for Detection. ACS Nano. 2020 Apr 9. doi: 10.1021/acsnano.0c02624. [Epubahead of print] PubMed PMID: 32223179; PubMed Central PMCID: PMC7144809.	No appropriate outcome
370: Cruz AT, Zeichner SL. COVID-19 in Children: Initial Characterization of the Pediatric Disease. Pediatrics. 2020 Mar 16. pii: e20200834. doi:10.1542/peds.2020-0834. [Epub ahead of print] PubMed PMID: 32179659.	No original article

1: Molloy EJ, Bearer CF. COVID-19 in children and altered inflammatory responses.Pediatr Res. 2020 Apr 3. doi: 10.1038/s41390-020-0881-y. [Epub ahead of print]PubMed PMID: 32244248.	No appropriate population sample
2: Liu H, Wang LL, Zhao SJ, Kwak-Kim J, Mor G, Liao AH. Why are pregnant womensusceptible to COVID-19? An immunological viewpoint. J Reprod Immunol. 2020 Mar19;139:103122. doi: 10.1016/j.jri.2020.103122. [Epub ahead of print] Review.PubMed PMID: 32244166.	No original article
3: Zhang B, Liu S, Tan T, Huang W, Dong Y, Chen L, Chen Q, Zhang L, Zhong Q, Zhang X, Zou Y, Zhang S. Treatment with convalescent plasma for critically ill patients with SARS-CoV-2 infection. Chest. 2020 Mar 31. pii:S0012-3692(20)30571-7. doi: 10.1016/j.chest.2020.03.039. [Epub ahead of print]	No useful data
4: Ngoi N, Lim J, Ow S, Jen WY, Lee M, Teo W, Ho J, Sundar R, Tung ML, Lee YM,Ngo E, Lim SW, Ong J, Lim F, Bonney G, Vellayappan B, Ho F, Tey J, Chan N, de MelS, Poon M, Lee SY, Koh LP, Liu TC, Tan LK, Wong A, Wong A, Lim SE, Yeoh A, WongJE, Tan D, Goh BC, Chng WJ, Soo R, Chee CE, Chee YL, Lee SC, Jeyasekharan AD;National University Cancer Institute, Singapore (NCIS). A segregated-team modelto maintain cancer care during the COVID-19 outbreak at an academic center inSingapore. Ann Oncol. 2020 Mar 31. pii: S0923-7534(20)36410-3. doi:10.1016/j.annonc.2020.03.306. [Epub ahead of print] PubMed PMID: 32243893.	No appropriate population sample
5: Dalton L, Rapa E, Stein A. Protecting the psychological health of childrenthrough effective communication about COVID-19. Lancet Child Adolesc Health. 2020Mar 31. pii: S2352-4642(20)30097-3. doi: 10.1016/S2352-4642(20)30097-3. [Epubahead of print] PubMed PMID: 32243784.	No appropriate outcome

6: Rosenthal DM, Ucci M, Heys M, Hayward A, Lakhanpaul M. Impacts of COVID-19 on vulnerable children in temporary accommodation in the UK. Lancet Public Health.2020 Mar 31. pii: S2468-2667(20)30080-3. doi: 10.1016/S2468-2667(20)30080-3.[Epub ahead of print] PubMed PMID: 32243776.	No appropriate population sample
7: Lin J, Duan J, Tan T, Fu Z, Dai J. The isolation period should be longer:Lesson from a child infected with SARS-CoV-2 in Chongqing, China. PediatrPulmonol. 2020 Apr 3. doi: 10.1002/ppul.24763. [Epub ahead of print] PubMed PMID:32243729	No appropriate population sample
8: Yonker LM, Shen K, Kinane TB. Lessons unfolding from pediatric cases ofCOVID-19 disease caused by SARS-CoV-2 infection. Pediatr Pulmonol. 2020 Apr 3.doi: 10.1002/ppul.24748. [Epub ahead of print] PubMed PMID: 32243722.	No appropriate population sample
9: Schiariti V. Los derechos humanos de los niños con discapacidad duranteemergencias sanitarias: el desafío de COVID-19. Dev Med Child Neurol. 2020 Apr 3.doi: 10.1111/dmcn.14529. [Epub ahead of print] PubMed PMID: 32243566.	No appropriate population sample
10: Shander A, Goobie SM, Warne MA, Aapro M, Bisbe E, Perez-Calatayud AA, Callum J, Cushing MM, Dyer WB, Erhard J, Faraoni D, Farmer S, Fedorova T, Frank SM,Froessler B, Gombotz H, Gross I, Guinn NR, Haas T, Hamdorf J, Isbister JP,Javidroozi M, Ji H, Kim YW, Kor DJ, Kurz J, Lasocki S, Leahy MF, Lee CK, Lee JJ, Louw V, Meier J, Mezzacasa A, Munoz M, Ozawa S, Pavesi M, Shander N, Spahn DR,Spiess BD, Thomson J, Trentino K, Zenger C, Hofmann A; International Foundationof Patient Blood Management (IFPBM) and Society for the Advancement of BloodManagement (SABM) Work Group. The Essential Role of Patient Blood Management in aPandemic: A Call for Action. Anesth Analg. 2020 Mar 31. doi:10.1213/ANE.0000000000004844. [Epub ahead of print] PubMed PMID: 32243296.	No appropriate outcome

11: Bong CL, Brasher C, Chikumba E, McDougall R, Mellin-Olsen J, Enright A. TheCOVID-19 Pandemic: Effects on Low and Middle-Income Countries. Anesth Analg. 2020Apr 1. doi: 10.1213/ANE.0000000000004846. [Epub ahead of print] PubMed PMID:32243287	No appropriate outcome
12: Davanzo R, Moro G, Sandri F, Agosti M, Moretti C, Mosca F. Breastfeeding and Coronavirus Disease-2019. Ad interim indications of the Italian Society ofNeonatology endorsed by the Union of European Neonatal & Perinatal Societies.Matern Child Nutr. 2020 Apr 3:e13010. doi: 10.1111/mcn.13010. [Epub ahead ofprint] Review. PubMed PMID: 32243068.	No original article
13: Skulstad H, Cosyns B, Popescu BA, Galderisi M, Salvo GD, Donal E, Petersen S,Gimelli A, Haugaa KH, Muraru D, Almeida AG, Schulz-Menger J, Dweck MR, Pontone G,Sade LE, Gerber B, Maurovich-Horvat P, Bharucha T, Cameli M, Magne J, Westwood M,Maurer G, Edvardsen T. COVID-19 pandemic and cardiac imaging: EACVIrecommendations on precautions, indications, prioritization, and protection forpatients and healthcare personnel. Eur Heart J Cardiovasc Imaging. 2020 Apr 3.pii: jeaa072. doi: 10.1093/ehjci/jeaa072. [Epub ahead of print] PubMed PMID:32242891	No appropriate population sample
14: Marinelli KA, Lawrence RM. Safe Handling of Containers of Expressed HumanMilk in all Settings During the SARS-CoV-2 (COVID-19) Pandemic. J Hum Lact. 2020 Apr 3:890334420919083. doi: 10.1177/0890334420919083. [Epub ahead of print]PubMed PMID: 32242762.	No appropriate outcome
15: Du Y, Tu L, Zhu P, Mu M, Wang R, Yang P, Wang X, Hu C, Ping R, Hu P, Li T,Cao F, Chang C, Hu Q, Jin Y, Xu G. Clinical Features of 85 Fatal Cases ofCOVID-19 from Wuhan: A Retrospective Observational Study. Am J Respir Crit CareMed. 2020 Apr 3. doi:	No appropriate population sample

10.1164/rccm.202003-0543OC. [Epub ahead of print] PubMedPMID: 32242738.	
16: Siskind D, Honer WG, Clark S, Correll CU, Hasan A, Howes O, Kane JM, KellyDL, Laitman R, Lee J, MacCabe JH, Myles N, Nielsen J, Schulte PF, Taylor D,Verdoux H, Wheeler A, Freudenreich O. Consensus statement on the use of clozapineduring the COVID-19 pandemic. J Psychiatry Neurosci. 2020 Apr 3;45(3):2. doi:10.1503/jpn.200061. [Epub ahead of print] PubMed PMID: 32242646.	No appropriate outcome
17: Kim S, Kim YJ, Peck KR, Jung E. School Opening Delay Effect on TransmissionDynamics of Coronavirus Disease 2019 in Korea: Based on Mathematical Modeling andSimulation Study. J Korean Med Sci. 2020 Apr 6;35(13):e143. doi:10.3346/jkms.2020.35.e143. PubMed PMID: 32242349.	No appropriate outcome
18: Kim ES, Chin BS, Kang CK, Kim NJ, Kang YM, Choi JP, Oh DH, Kim JH, Koh B, KimSE, Yun NR, Lee JH, Kim JY, Kim Y, Bang JH, Song KH, Kim HB, Chung KH, Oh MD;Korea National Committee for Clinical Management of COVID-19. Clinical Course andOutcomes of Patients with Severe Acute Respiratory Syndrome Coronavirus 2Infection: a Preliminary Report of the First 28 Patients from the Korean CohortStudy on COVID-19. J Korean Med Sci. 2020 Apr 6;35(13):e142. doi:10.3346/jkms.2020.35.e142. PubMed PMID: 32242348.	No appropriate population sample
19: Kimmig R, Verheijen RHM, Rudnicki M; SERGS Council. Robot assisted surgeryduring the COVID-19 pandemic, especially for gynecological cancer: a statement ofthe Society of European Robotic Gynaecological Surgery (SERGS). J Gynecol Oncol. 2020 Apr 3. doi: 10.3802/jgo.2020.31.e59. [Epub ahead of print] PubMed PMID:32242340	No appropriate population sample

20: Scuccimarri R, Sutton E, Fitzcharles MA. Hydroxychloroquine: a potentialethical dilemma for rheumatologists during the COVID-19 pandemic. J Rheumatol.2020 Apr 2. pii: jrheum.200369. doi: 10.3899/jrheum.200369. [Epub ahead of print]PubMed PMID: 32241801.	No appropriate outcome
21: Dong Y, Wang L, Burgner DP, Miller JE, Song Y, Ren X, Li Z, Xing Y, Ma J,Sawyer SM, Patton GC. Infectious diseases in children and adolescents in China:analysis of national surveillance data from 2008 to 2017. BMJ. 2020 Apr2;369:m1043. doi: 10.1136/bmj.m1043. PubMed PMID: 32241761.	No appropriate population sample
22: Calvo C, Tagarro A, Otheo E, Epalza C; Grupo de Seguimiento de la Infecciónpor SARS-CoV-2 en la Comunidad de Madrid; Miembros del Grupo de Seguimiento de laInfección por SARS-CoV-2 en la Comunidad de Madrid. [Epidemiological update onSARS-CoV-2 infection in Spain. Comments on the management of infection inpediatrics]. An Pediatr (Barc). 2020 Mar 30. pii: S1695-4033(20)30097-7. doi:10.1016/j.anpedi.2020.03.001. [Epub ahead of print] Spanish. PubMed PMID:32241653	No appropriate population sample
23: Qiu L, Liu X, Xiao M, Xie J, Cao W, Liu Z, Morse A, Xie Y, Li T, Zhu L.SARS-CoV-2 is not detectable in the vaginal fluid of women with severe COVID-19infection. Clin Infect Dis. 2020 Apr 2. pii: ciaa375. doi: 10.1093/cid/ciaa375.[Epub ahead of print] PubMed PMID: 32241022.	No appropriate population sample
24: Salman S, Salem ML. Routine childhood immunization may protect againstCOVID-19. Med Hypotheses. 2020 Mar 25;140:109689. doi:10.1016/j.mehy.2020.109689. [Epub ahead of print] PubMed PMID: 32240961.	No appropriate population sample
25: Weaver MS, Wiener L. Applying Palliative Care Principles to Communicate with Children about COVID-19. J Pain Symptom Manage. 2020 Mar 30. pii:S0885-3924(20)30171-8. doi:	No appropriate outcome

10.1016/j.jpainsymman.2020.03.020. [Epub ahead ofprint] PubMed PMID: 32240751.	
26: Giulio M, Maggioni D, Montroni I, Ugolini G, Capelli P, Ceppi L, Bonfanti P, Mariani A, Achilli F. Being a doctor will never be the same after the COVID-19pandemic. Am J Med. 2020 Mar 30. pii: S0002-9343(20)30216-3. doi:10.1016/j.amjmed.2020.03.003. [Epub ahead of print] PubMed PMID: 32240630.	No appropriate outcome
27: Green CA, Quraishi MN, Shabir S, Sharma N, Hansen R, Gaya DR, Hart AL, Loman NJ, Iqbal TH. Screening faecal microbiota transplant donors for SARS-CoV-2 bymolecular testing of stool is the safest way forward. Lancet GastroenterolHepatol. 2020 Mar 30. pii: S2468-1253(20)30089-3. doi:10.1016/S2468-1253(20)30089-3. [Epub ahead of print] PubMed PMID: 32240618.	No appropriate population sample
28: Casanova M, Bagliacca EP, Silva M, Patriarca C, Veneroni L, Clerici CA,Spreafico F, Luksch R, Terenziani M, Meazza C, Podda M, Biassoni V, Schiavello E,Chiaravalli S, Puma N, Bergamaschi L, Gattuso G, Sironi G, Massimino M, FerrariA. How young patients with cancer perceive the Covid-19 (coronavirus) epidemic inMilan, Italy: is there room for other fears? Pediatr Blood Cancer. 2020 Apr2:e28318. doi: 10.1002/pbc.28318. [Epub ahead of print] PubMed PMID: 32240567.	No appropriate population sample
29: Turer RW, Jones I, Rosenbloom ST, Slovis C, Ward MJ. Electronic PersonalProtective Equipment: A Strategy to Protect Emergency Department Providers in theAge of COVID-19. J Am Med Inform Assoc. 2020 Apr 2. pii: ocaa048. doi:10.1093/jamia/ocaa048. [Epub ahead of print] PubMed PMID: 32240303.	No appropriate outcome

30: Geldsetzer P. Use of Rapid Online Surveys to Assess People's PerceptionsDuring Infectious Disease Outbreaks: A Cross-sectional Survey on COVID-19. J Med Internet Res. 2020 Apr 2;22(4):e18790. doi: 10.2196/18790. PubMed PMID: 32240094.	No appropriate outcome
31: Bouffet E, Challinor J, Sullivan M, Biondi A, Rodriguez-Galindo C,Pritchard-Jones K. Early advice on managing children with cancer during theCOVID-19 pandemic and a call for sharing experiences. Pediatr Blood Cancer. 2020 Apr 2:e28327. doi: 10.1002/pbc.28327. [Epub ahead of print] PubMed PMID:32239747	No appropriate population sample
32: Lovato A, Rossettini G, de Filippis C. Sore throat in COVID-19: comment onClinical characteristics of hospitalized patients with SARS-CoV-2 infection: A single arm meta-analysis. J Med Virol. 2020 Apr 1. doi: 10.1002/jmv.25815. [Epubahead of print] PubMed PMID: 32239511.	No appropriate population sample
33: Agostini A, Floridi C, Borgheresi A, Badaloni M, Esposto Pirani P, Terilli F,Ottaviani L, Giovagnoni A. Proposal of a low-dose, long-pitch, dual-source chest CT protocol on third-generation dual-source CT using a tin filter for spectralshaping at 100 kVp for CoronaVirus Disease 2019 (COVID-19) patients: afeasibility study. Radiol Med. 2020 Apr 1. doi: 10.1007/s11547-020-01179-x. [Epubahead of print] PubMed PMID: 32239472.	No appropriate population sample
34: Chen Z, He S, Li F, Yin J, Chen X. Mobile field hospitals, an effective wayof dealing with COVID-19 in China: sharing our experience. Biosci Trends. 2020Apr 1. doi: 10.5582/bst.2020.01110. [Epub ahead of print] PubMed PMID: 32238673.	No appropriate outcome
35: Chawla D, Chirla D, Dalwai S, Deorari AK, Ganatra A, Gandhi A, Kabra NS,Kumar P, Mittal P, Parekh BJ, Sankar MJ, Singhal T, Sivanandan S, Tank P;Federation Of Obstetric And Gynecological Societies Of India (FOGSI), NationalNeonatology Forum Of India (NNF), And Indian Academy Of	No original article

Pediatrics (IAP).Perinatal-Neonatal Management of COVID-19 Infection - Guidelines of theFederation of Obstetric and Gynecological Societies of India (FOGSI), NationalNeonatology Forum of India (NNF), and Indian Academy of Pediatrics (IAP). Indian Pediatr. 2020 Apr 1. pii: S097475591600154. [Epub ahead of print] PubMed PMID:32238615	
36: Shanker V. Measles Immunization: Worth Considering Containment Strategy forSARS-CoV-2 Global Outbreak. Indian Pediatr. 2020 Mar 29. pii: S097475591600153.[Epub ahead of print] PubMed PMID: 32238614.	No appropriate outcome
37: Sundaram M, Ravikumar N, Bansal A, Nallasamy K, Basavaraja GV, Lodha R, GuptaD, Odena MP, Ashwath RNR, Jayashree M; For Intensive Care Chapter Of IndianAcademy Of Pediatrics. Novel Coronavirus 2019 (2019-nCoV) Infection: Part II -Respiratory Support in the Pediatric Intensive Care Unit in Resource-limitedSettings. Indian Pediatr. 2020 Mar 29. pii: S097475591600152. [Epub ahead ofprint] PubMed PMID: 32238613.	No appropriate population sample
38: Ravikumar N, Nallasamy K, Bansal A, Angurana SK, Basavaraja GV, Sundaram M,Lodha R, Gupta D, Jayashree M; For Intensive Care Chapter Of Indian Academy OfPediatrics. Novel Coronavirus 2019 (2019-nCoV) Infection: Part I - Preparednessand Management in the Pediatric Intensive Care Unit in Resource-limited Settings.Indian Pediatr. 2020 Mar 29. pii: S097475591600151. [Epub ahead of print] PubMed PMID: 32238612.	No appropriate population sample
39: Karimi-Zarchi M, Neamatzadeh H, Dastgheib SA, Abbasi H, Mirjalili SR,Behforouz A, Ferdosian F, Bahrami R. Vertical Transmission of Coronavirus Disease19 (COVID-19) from Infected Pregnant Mothers to Neonates: A Review. Fetal PediatrPathol. 2020 Apr 2:1-5. doi: 10.1080/15513815.2020.1747120. [Epub ahead of print]PubMed PMID: 32238084.	No original article

40: Broxmeyer HE, Parker GC. Impact of COVID-19 and Future Emerging Viruses onHematopoietic Cell Transplantation and Other Cellular Therapies. Stem Cells Dev. 2020 Apr 3. doi: 10.1089/scd.2020.0064. [Epub ahead of print] PubMed PMID:32237972	No appropriate outcome
41: Saghazadeh A, Rezaei N. Immune-epidemiological parameters of the novelcoronavirus - a perspective. Expert Rev Clin Immunol. 2020 Apr 1. doi:10.1080/1744666X.2020.1750954. [Epub ahead of print] PubMed PMID: 32237901.	No original article
42: Ye Q, Wang B, Mao J, Fu J, Shang S, Shu Q, Zhang T. Epidemiological analysis of COVID-19 and practical experience from China. J Med Virol. 2020 Apr 1. doi:10.1002/jmv.25813. [Epub ahead of print] Review. PubMed PMID: 32237160.	No original article
43: Yung CF, Kam KQ, Wong MSY, Maiwald M, Tan YK, Tan BH, Thoon KC. Environmentand Personal Protective Equipment Tests for SARS-CoV-2 in the Isolation Room ofan Infant With Infection. Ann Intern Med. 2020 Apr 1. doi: 10.7326/M20-0942.[Epub ahead of print] PubMed PMID: 32236490.	No appropriate population sample
44: Lazzeri M, Lanza A, Bellini R, Bellofiore A, Cecchetto S, Colombo A,D'Abrosca F, Del Monaco C, Gaudiello G, Paneroni M, Privitera E, Retucci M, RossiV, Santambrogio M, Sommariva M, Frigerio P. Respiratory physiotherapy in patientswith COVID-19 infection in acute setting: a Position Paper of the ItalianAssociation of Respiratory Physiotherapists (ARIR). Monaldi Arch Chest Dis. 2020 Mar 26;90(1). doi: 10.4081/monaldi.2020.1285. PubMed PMID: 32236089.	No appropriate population sample
45: Turner D, Huang Y, Martín-de-Carpi J, Aloi M, Focht G, Kang B, Zhou Y,Sanchez C, Kappelman MD, Uhlig HH, Pujol-Muncunill G, Ledder O, Lionetti P, Dias JA, Ruemmele FM, Russell RK; Paediatric IBD Porto group of ESPGHAN. COVID-19 and Paediatric Inflammatory Bowel Diseases: Global	No appropriate population sample

Experience and ProvisionalGuidance (March 2020) from the Paediatric IBD Porto group of ESPGHAN. J PediatrGastroenterol Nutr. 2020 Mar 31. doi: 10.1097/MPG.0000000000002729. [Epub aheadof print] PubMed PMID: 32235161.	
46: Tian H, Liu Y, Li Y, Wu CH, Chen B, Kraemer MUG, Li B, Cai J, Xu B, Yang Q,Wang B, Yang P, Cui Y, Song Y, Zheng P, Wang Q, Bjornstad ON, Yang R, GrenfellBT, Pybus OG, Dye C. An investigation of transmission control measures during thefirst 50 days of the COVID-19 epidemic in China. Science. 2020 Mar 31. pii:eabb6105. doi: 10.1126/science.abb6105. [Epub ahead of print] PubMed PMID:32234804	No appropriate outcome
47: Hagmann SHF. COVID-19 in children: More than meets the eye. Travel Med InfectDis. 2020 Mar 28:101649. doi: 10.1016/j.tmaid.2020.101649. [Epub ahead of print] PubMed PMID: 32234457.	No appropriate population sample
49: Feng K, Yun YX, Wang XF, Yang GD, Zheng YJ, Lin CM, Wang LF. [Analysis of CT features of 15 children with 2019 novel coronavirus infection]. Zhonghua Er Ke ZaZhi. 2020 Apr 2;58(4):275-278. doi: 10.3760/cma.j.cn112140-20200210-00071.Chinese. PubMed PMID: 32234131.	No appropriate population sample
50: Mark K, Steel K, Stevenson J, Evans C, McCormick D, Willocks L, McCallum A,Jones L, Johannessen I, Templeton K, Koch O, Mackintosh C. Coronavirus disease(COVID-19) Community Testing Team in Scotland: A 14-day review, 6 to 20 February 2020. Euro Surveill. 2020 Mar;25(12). doi:10.2807/1560-7917.ES.2020.25.12.2000217. PubMed PMID: 32234116.	No appropriate outcome
51: Hartley DM, Reisinger HS, Perencevich EN. When infection prevention entersthe temple: Intergenerational social distancing and COVID-19. Infect Control HospEpidemiol. 2020 Apr 1:1-3. doi: 10.1017/ice.2020.100. [Epub ahead of print]PubMed PMID: 32234091.	No appropriate population sample

52: Polites SF, Azarow KS. Perspectives on Pediatric Appendicitis andAppendectomy During the Severe Acute Respiratory Syndrome Coronavirus 2 Pandemic.J Laparoendosc Adv Surg Tech A. 2020 Apr 1. doi: 10.1089/lap.2020.0197. [Epubahead of print] PubMed PMID: 32233967.	No appropriate population sample
53: Kamali Aghdam M, Jafari N, Eftekhari K. Novel coronavirus in a 15-day-oldneonate with clinical signs of sepsis, a case report. Infect Dis (Lond). 2020 Apr1:1-3. doi: 10.1080/23744235.2020.1747634. [Epub ahead of print] PubMed PMID:32233816	Included
54: Korean Society of Infectious Diseases and Korea Centers for Disease Controland Prevention. Analysis on 54 Mortality Cases of Coronavirus Disease 2019 in theRepublic of Korea from January 19 to March 10, 2020. J Korean Med Sci. 2020 Mar30;35(12):e132. doi: 10.3346/jkms.2020.35.e132. PubMed PMID: 32233161.	No appropriate population sample
55: Gandolfini I, Delsante M, Fiaccadori E, Zaza G, Manenti L, Degli Antoni A,Peruzzi L, Riella LV, Cravedi P, Maggiore U. COVID-19 in Kidney TransplantRecipients. Am J Transplant. 2020 Mar 31. doi: 10.1111/ajt.15891. [Epub ahead of print] PubMed PMID: 32233067.	No appropriate population sample
56: Abu-Rustum RS, Akolekar R, Sotiriadis A, Salomon LJ, Da Silva Costa F, Wu Q, Frusca T, Bilardo CM, Prefumo F, Poon LC. ISUOG Consensus Statement onorganization of routine and specialist obstetric ultrasound services in thecontext of COVID-19. Ultrasound Obstet Gynecol. 2020 Mar 31. doi:10.1002/uog.22029. [Epub ahead of print] PubMed PMID: 32233049.	No original article
57: Bousquet J, Akdis C, Jutel M, Bachert C, Klimek L, Agache I, Ansotegui IJ,Bedbrook A, Bosnic-Anticevich S, Canonica GW, Chivato T, Cruz AA, Czarlewski W,Del Giacco S, Du H, Fonseca JA, Gao Y, Haahtela T, Hoffmann-Sommergruber K,Ivancevich JC, Khaltaev N, Knol EF, Kuna P, Larenas-Linnemann D, Mullol J,Naclerio R, Ohta K, Okamoto Y,	No appropriate population sample

O'Mahony L, Onorato GL, Papadopoulos NG, Pfaar O, Samolinski B, Schwarze J, Toppila-Salmi S, Teresa Ventura M, Valiulis A,Yorgancioglu A, Zuberbier T; ARIA-MASK study group. Intranasal corticosteroids inallergic rhinitis in COVID-19 infected patients: An ARIA-EAACI statement.Allergy. 2020 Mar 31. doi: 10.1111/all.14302. [Epub ahead of print] PubMed PMID: 32233040	
58: Jiatong S, Lanqin L, Wenjun L. COVID-19 epidemic: disease characteristics in children. J Med Virol. 2020 Mar 31. doi: 10.1002/jmv.25807. [Epub ahead of print]Review. PubMed PMID: 32232980.	No original article
59: Giovagnoni A. Facing the COVID-19 emergency: we can and we do. Radiol Med.2020 Mar 30. doi: 10.1007/s11547-020-01178-y. [Epub ahead of print] PubMed PMID: 32232651	No appropriate outcome
60: Panahi L, Amiri M, Pouy S. Risks of Novel Coronavirus Disease (COVID-19) inPregnancy; a Narrative Review. Arch Acad Emerg Med. 2020 Mar 23;8(1):e34.eCollection 2020. Review. PubMed PMID: 32232217; PubMed Central PMCID:PMC7092922.	No original article
61: Lee DH, Lee J, Kim E, Woo K, Park HY, An J. Emergency cesarean section onsevere acute respiratory syndrome coronavirus 2 (SARS- CoV-2) confirmed patient. Korean J Anesthesiol. 2020 Mar 31. doi: 10.4097/kja.20116. [Epub ahead of print] PubMed PMID: 32229802.	Included
62: Cao W, Fang Z, Hou G, Han M, Xu X, Dong J, Zheng J. The psychological impact of the COVID-19 epidemic on college students in China. Psychiatry Res. 2020 Mar20;287:112934. doi: 10.1016/j.psychres.2020.112934. [Epub ahead of print] PubMed PMID: 32229390; PubMed Central PMCID: PMC7102633.	No appropriate population sample
63: Rahimi F, Talebi Bezmin Abadi A. Practical Strategies Against the NovelCoronavirus and COVID-19-the Imminent Global Threat. Arch Med Res. 2020 Mar 27.pii: S0188-4409(20)30287-3. doi:	No appropriate outcome

10.1016/j.arcmed.2020.03.005. [Epub ahead ofprint] PubMed PMID: 32229157.	
64: South AM, Diz D, Chappell MC. COVID-19, ACE2 and the CardiovascularConsequences. Am J Physiol Heart Circ Physiol. 2020 Mar 31. doi:10.1152/ajpheart.00217.2020. [Epub ahead of print] PubMed PMID: 32228252.	No appropriate outcome
65: Dunn CG, Kenney E, Fleischhacker SE, Bleich SN. Feeding Low-Income Childrenduring the Covid-19 Pandemic. N Engl J Med. 2020 Mar 30. doi:10.1056/NEJMp2005638. [Epub ahead of print] PubMed PMID: 32227759.	No appropriate outcome
66: Rundle AG, Park Y, Herbstman JB, Kinsey EW, Wang YC. COVID-19 Related School Closings and Risk of Weight Gain Among Children. Obesity (Silver Spring). 2020Mar 30. doi: 10.1002/oby.22813. [Epub ahead of print] PubMed PMID: 32227671.	No appropriate outcome
67: Szperka CL, Ailani J, Barmherzig R, Klein BC, Minen MT, Halker Singh RB,Shapiro RE. Migraine Care in the Era of COVID-19: Clinical Pearls and Plea toInsurers. Headache. 2020 Mar 30. doi: 10.1111/head.13810. [Epub ahead of print]PubMed PMID: 32227596.	No appropriate population sample
68: Soldati G, Smargiassi A, Inchingolo R, Buonsenso D, Perrone T, Briganti DF,Perlini S, Torri E, Mariani A, Mossolani EE, Tursi F, Mento F, Demi L. Proposalfor international standardization of the use of lung ultrasound for COVID-19patients; a simple, quantitative, reproducible method. J Ultrasound Med. 2020 Mar30. doi: 10.1002/jum.15285. [Epub ahead of print] PubMed PMID: 32227492.	No original article
69: Cassaniti I, Novazzi F, Giardina F, Salivaro F, Sachs M, Perlini S, Bruno R, Mojoli F, Baldanti F; San Matteo Pavia COVID-19 Task Force. Performance ofVivaDiagTM COVID-19 IgM/IgG Rapid Test is inadequate	No appropriate population sample

for diagnosis of COVID-19 inacute patients referring to emergency room department. J Med Virol. 2020 Mar 30. doi: 10.1002/jmv.25800. [Epub ahead of print] PubMed PMID: 32227490.	
70: Schiariti V. The human rights of children with disabilities during healthemergencies: the challenge of COVID-19. Dev Med Child Neurol. 2020 Mar 30. doi:10.1111/dmcn.14526. [Epub ahead of print] PubMed PMID: 32227474.	No appropriate population sample
71: Zhu H, Wei L, Niu P. The novel coronavirus outbreak in Wuhan, China. GlobHealth Res Policy. 2020 Mar 2;5:6. doi: 10.1186/s41256-020-00135-6. eCollection2020. PubMed PMID: 32226823; PubMed Central PMCID: PMC7050114.	No appropriate outcome
72: Sifuentes-Rodríguez E, Palacios-Reyes D. COVID-19: The outbreak caused by anew coronavirus. Bol Med Hosp Infant Mex. 2020;77(2):47-53. doi:10.24875/BMHIM.20000039. Review. PubMed PMID: 32226003.	No appropriate outcome
73: Simonato A, Giannarini G, Abrate A, Bartoletti R, Crestani A, De Nunzio C,Gregori A, Liguori G, Novara G, Pavan N, Trombetta C, Tubaro A, Porpiglia F,Ficarra V; Members of the Research Urology Network (RUN). Pathways for urologypatients during the COVID-19 pandemic. Minerva Urol Nefrol. 2020 Mar 30. doi:10.23736/S0393-2249.20.03861-8. [Epub ahead of print] PubMed PMID: 32225135.	No appropriate population sample
74: Alhazzani W, Møller MH, Arabi YM, Loeb M, Gong MN, Fan E, Oczkowski S, LevyMM, Derde L, Dzierba A, Du B, Aboodi M, Wunsch H, Cecconi M, Koh Y, Chertow DS,Maitland K, Alshamsi F, Belley-Cote E, Greco M, Laundy M, Morgan JS, KeseciogluJ, McGeer A, Mermel L, Mammen MJ, Alexander PE, Arrington A, Centofanti JE,Citerio G, Baw B, Memish ZA, Hammond N, Hayden FG, Evans L, Rhodes A. SurvivingSepsis Campaign: Guidelines on the Management of Critically Ill Adults withCoronavirus Disease	No appropriate population sample

2019 (COVID-19). Crit Care Med. 2020 Mar 27. doi:10.1097/CCM.0000000000004363. [Epub ahead of print] PubMed PMID: 32224769.	
75: Paintsil E. COVID-19 threatens health systems in sub-Saharan Africa: the eye of the crocodile. J Clin Invest. 2020 Mar 30. pii: 138493. doi:10.1172/JCI138493. [Epub ahead of print] PubMed PMID: 32224550.	No appropriate outcome
76: Rajgor DD, Lee MH, Archuleta S, Bagdasarian N, Quek SC. The many estimates ofthe COVID-19 case fatality rate. Lancet Infect Dis. 2020 Mar 27. pii:S1473-3099(20)30244-9. doi: 10.1016/S1473-3099(20)30244-9. [Epub ahead of print] PubMed PMID: 32224313.	No appropriate outcome
77: Sinha IP, Harwood R, Semple MG, Hawcutt DB, Thursfield R, Narayan O, KennySE, Viner R, Hewer SL, Southern KW. COVID-19 infection in children. Lancet RespirMed. 2020 Mar 27. pii: S2213-2600(20)30152-1. doi: 10.1016/S2213-2600(20)30152-1.[Epub ahead of print] PubMed PMID: 32224304.	No original article
78: Liu JJ, Bao Y, Huang X, Shi J, Lu L. Mental health considerations forchildren quarantined because of COVID-19. Lancet Child Adolesc Health. 2020 Mar27. pii: S2352-4642(20)30096-1. doi: 10.1016/S2352-4642(20)30096-1. [Epub aheadof print] PubMed PMID: 32224303.	No appropriate outcome
79: Shaker MS, Oppenheimer J, Grayson M, Stukus D, Hartog N, Hsieh EWY, Rider N, Dutmer CM, Vander Leek TK, Kim H, Chan ES, Mack D, Ellis AK, Lang D, Lieberman J,Fleischer D, Golden DBK, Wallace D, Portnoy J, Mosnaim G, Greenhawt M. COVID-19: Pandemic Contingency Planning for the Allergy and Immunology Clinic. J AllergyClin Immunol Pract. 2020 Mar 26. pii: S2213-2198(20)30253-1. doi:	No appropriate outcome

80: Huang YC, Lee PI, Hsueh PR. Evolving reporting criteria of COVID-19 in Taiwanduring the epidemic. J Microbiol Immunol Infect. 2020 Mar 19. pii:S1684-1182(20)30074-8. doi: 10.1016/j.jmii.2020.03.014. [Epub ahead of print]PubMed PMID: 32224117.	No appropriate outcome
81: Ma X, Su L, Zhang Y, Zhang X, Gai Z, Zhang Z. Do children need a longer time to shed SARS-CoV-2 in stool than adults? J Microbiol Immunol Infect. 2020 Mar 19.pii: S1684-1182(20)30070-0. doi: 10.1016/j.jmii.2020.03.010. [Epub ahead ofprint] PubMed PMID: 32224116.	No appropriate population sample
82: COVID-19 National Incident Room Surveillance Team. COVID-19, Australia:Epidemiology Report 8 (Reporting period from 19:00 AEDT 14 March to 23:59 AEDT 22March 2020). Commun Dis Intell (2018). 2020 Mar 27;44. doi:10.33321/cdi.2020.44.28. PubMed PMID: 32223725.	No appropriate outcome
83: Marinelli KA. International Perspectives Concerning Donor Milk Banking Duringthe SARS-CoV-2 (COVID-19) Pandemic. J Hum Lact. 2020 Mar 30:890334420917661. doi:10.1177/0890334420917661. [Epub ahead of print] PubMed PMID: 32223581.	No appropriate outcome
84: Capanna F, Haydar A, McCarey C, Bernini Carri E, Bartha Rasero J, TsibizovaV, Helmer H, Makatsarya A, Di Renzo GC. Preparing an obstetric unit in the heart of the epidemic strike of COVID-19: quick reorganization tips. J Matern FetalNeonatal Med. 2020 Mar 29:1-11. doi: 10.1080/14767058.2020.1749258. [Epub aheadof print] PubMed PMID: 32223490.	No original article
85: Mirzadeh M, Khedmat L. Pregnant Women in the Exposure to COVID-19 InfectionOutbreak: The Unseen Risk Factors and Preventive Healthcare Patterns. J MaternFetal Neonatal Med. 2020 Mar 29:1-4. doi: 10.1080/14767058.2020.1749257. [Epubahead of print] PubMed PMID: 32223477.	No original article

86: Wollenberg A, Flohr C, Simon D, Cork MJ, Thyssen JP, Bieber T, deBruin-Weller MS, Weidinger S, Deleuran M, Taieb A, Paul C, Trzeciak M, Werfel T, Seneschal J, Barbarot S, Darsow U, Torrelo A, Stalder JF, Svensson Å, Hijnen D,Gelmetti C, Szalai Z, Gieler U, De Raeve L, Kunz B, Spuls P, von Kobyletzki LB,Fölster-Holst R, Chernyshov PV, Cristen-Zaech S, Heratizadeh A, Ring J,Vestergaard C. European Task Force on Atopic Dermatitis (ETFAD) statement onsevere acute respiratory syndrome coronavirus 2 (SARS-Cov-2)-infection and atopicdermatitis. J Eur Acad Dermatol Venereol. 2020 Mar 29. doi: 10.1111/jdv.16411.[Epub ahead of print] PubMed PMID: 32223003.	No appropriate population sample
87: Zehender G, Lai A, Bergna A, Meroni L, Riva A, Balotta C, Tarkowski M,Gabrieli A, Bernacchia D, Rusconi S, Rizzardini G, Antinori S, Galli M. GENOMICCHARACTERISATION AND PHYLOGENETIC ANALYSIS OF SARS-COV-2 IN ITALY. J Med Virol.2020 Mar 29. doi: 10.1002/jmv.25794. [Epub ahead of print] PubMed PMID: 32222993.	No appropriate outcome
88: Zhang T, Cui X, Zhao X, Wang J, Zheng J, Zheng G, Guo W, Cai C, He S, Xu Y.Detectable SARS-CoV-2 Viral RNA in Feces of Three Children during Recovery Periodof COVID-19 Pneumonia. J Med Virol. 2020 Mar 29. doi: 10.1002/jmv.25795. [Epubahead of print] PubMed PMID: 32222992.	No appropriate population sample
89: Tian Y, Rong L, Nian W, He Y. Review article: gastrointestinal features inCOVID-19 and the possibility of faecal transmission. Aliment Pharmacol Ther. 2020Mar 29. doi: 10.1111/apt.15731. [Epub ahead of print] Review. PubMed PMID:32222988	No original article
90: Henry BM, Lippi G. Chronic kidney disease is associated with severecoronavirus disease 2019 (COVID-19) infection. Int Urol Nephrol. 2020 Mar 28.doi: 10.1007/s11255-020-02451-9. [Epub ahead of print] PubMed PMID: 32222883.	No appropriate population sample

92: Zambrano LI, Fuentes-Barahona IC, Bejarano-Torres DA, Bustillo C, Gonzales G,Vallecillo-Chinchilla G, Sanchez-Martínez FE, Valle-Reconco JA, Sierra M,Bonilla-Aldana DK, Cardona-Ospina JA, Rodríguez-Morales AJ. A pregnant woman withCOVID-19 in Central America. Travel Med Infect Dis. 2020 Mar 25:101639. doi:10.1016/j.tmaid.2020.101639. [Epub ahead of print] PubMed PMID: 32222420.	Included
93: La Marca A, Niederberger C, Pellicer A, Nelson SM. COVID-19: lessons from theItalian reproductive medical experience. Fertil Steril. 2020 Mar 25. pii:S0015-0282(20)30297-1. doi: 10.1016/j.fertnstert.2020.03.021. [Epub ahead ofprint] PubMed PMID: 32222253.	No appropriate population sample
94: Li Y, Hu Y, Zhang X, Yu Y, Li B, Wu J, Wu Y, Xia X, Xu J. [Follow-up testing of viral nucleic acid in discharged patients with moderate type of 2019coronavirus disease (COVID-19)]. Zhejiang Da Xue Xue Bao Yi Xue Ban. 2020 May25;49(1):0. Chinese. PubMed PMID: 32222122.	No appropriate population sample
95: Chen S, Liao E, Shao Y. Clinical analysis of pregnant women with 2019 novelcoronavirus pneumonia. J Med Virol. 2020 Mar 28. doi: 10.1002/jmv.25789. [Epubahead of print] PubMed PMID: 32222119.	Overlapping data
96: Abdelmaksoud A, Kroumpouzos G, Jafferany M, Lotti T, Sadoughifar R, GoldustM. COVID-19 in the Pediatric Population. Dermatol Ther. 2020 Mar 27:e13339. doi: 10.1111/dth.13339. [Epub ahead of print] PubMed PMID: 32222027.	No original article
97: Yang C. Does hand hygiene reduce SARS-CoV-2 transmission? Graefes Arch ClinExp Ophthalmol. 2020 Mar 27. doi: 10.1007/s00417-020-04652-5. [Epub ahead ofprint] PubMed PMID: 32221693.	No appropriate outcome

98: Wurcel AG, Dauria E, Zaller N, Nijhawan A, Beckwith C, Nowotny K,Brinkley-Rubinstein L. Spotlight on Jails: COVID-19 Mitigation Policies NeededNow. Clin Infect Dis. 2020 Mar 28. pii: ciaa346. doi: 10.1093/cid/ciaa346. [Epub ahead of print] PubMed PMID: 32221515.	No appropriate outcome
99: Chidini G, Villa C, Calderini E, Marchisio P, De Luca D. SARS-CoV-2 Infectionin a Pediatric Department in Milan: A Logistic Rather Than a Clinical Emergency. Pediatr Infect Dis J. 2020 Mar 25. doi: 10.1097/INF.0000000000002687. [Epub aheadof print] PubMed PMID: 32221165.	No appropriate population sample
100: Ramirez PT, Chiva L, Eriksson AGZ, Frumovitz M, Fagotti A, Gonzalez MartinA, Jhingran A, Pareja R. COVID-19 Global Pandemic: Options for Management ofGynecologic Cancers. Int J Gynecol Cancer. 2020 Mar 27. pii: ijgc-2020-001419.doi: 10.1136/ijgc-2020-001419. [Epub ahead of print] PubMed PMID: 32221023.	No appropriate population sample
101: Sparks MA, South A, Welling P, Luther JM, Cohen J, Byrd JB, Burrell LM,Batlle D, Tomlinson L, Bhalla V, Rheault MN, Soler MJ, Swaminathan S, Hiremath S.Sound Science before Quick Judgement Regarding RAS Blockade in COVID-19. Clin JAm Soc Nephrol. 2020 Mar 27. pii: CJN.03530320. doi: 10.2215/CJN.03530320. [Epub ahead of print] PubMed PMID: 32220930.	No appropriate outcome
102: Kotecha RS. Challenges posed by COVID-19 to children with cancer. LancetOncol. 2020 Mar 25. pii: S1470-2045(20)30205-9. doi:10.1016/S1470-2045(20)30205-9. [Epub ahead of print] PubMed PMID: 32220660.	No appropriate population sample
103: Cluver L, Lachman JM, Sherr L, Wessels I, Krug E, Rakotomalala S, Blight S, Hillis S, Bachman G, Green O, Butchart A, Tomlinson M, Ward CL, Doubt J, McDonaldK. Parenting in a time of COVID-19. Lancet. 2020 Mar 25. pii:S0140-6736(20)30736-4. doi: 10.1016/S0140-	No original article

6736(20)30736-4. [Epub ahead of print] PubMed PMID: 32220657.	
104: Prem K, Liu Y, Russell TW, Kucharski AJ, Eggo RM, Davies N; Centre for theMathematical Modelling of Infectious Diseases COVID-19 Working Group, Jit M,Klepac P. The effect of control strategies to reduce social mixing on outcomes ofthe COVID-19 epidemic in Wuhan, China: a modelling study. Lancet Public Health.2020 Mar 25. pii: S2468-2667(20)30073-6. doi: 10.1016/S2468-2667(20)30073-6.[Epub ahead of print] PubMed PMID: 32220655.	No appropriate outcome
105: Kelvin AA, Halperin S. COVID-19 in children: the link in the transmissionchain. Lancet Infect Dis. 2020 Mar 25. pii: S1473-3099(20)30236-X. doi:10.1016/S1473-3099(20)30236-X. [Epub ahead of print] PubMed PMID: 32220651.	No appropriate outcome
106: Qiu H, Wu J, Hong L, Luo Y, Song Q, Chen D. Clinical and epidemiologicalfeatures of 36 children with coronavirus disease 2019 (COVID-19) in Zhejiang,China: an observational cohort study. Lancet Infect Dis. 2020 Mar 25. pii:S1473-3099(20)30198-5. doi: 10.1016/S1473-3099(20)30198-5. [Epub ahead of print] PubMed PMID: 32220650.	No appropriate population sample
107: Portnoy J, Waller M, Elliott T. Telemedicine in the Era of COVID-19. JAllergy Clin Immunol Pract. 2020 Mar 24. pii: S2213-2198(20)30249-X. doi:10.1016/j.jaip.2020.03.008. [Epub ahead of print] PubMed PMID: 32220575; PubMedCentral PMCID: PMC7104202.	No appropriate outcome
108: Hsih WH, Cheng MY, Ho MW, Chou CH, Lin PC, Chi CY, Liao WC, Chen CY, LeongLY, Tien N, Lai HC, Lai YC, Lu MC. Featuring COVID-19 cases via screeningsymptomatic patients with epidemiologic link during flu season in a medicalcenter of central Taiwan. J Microbiol Immunol Infect. 2020 Mar 13. pii:S1684-	No appropriate population sample

1182(20)30068-2. doi: 10.1016/j.jmii.2020.03.008. [Epub ahead of print]PubMed PMID: 32220574; PubMed Central PMCID: PMC7102665.	
109: Majumder MS, Mandl KD. Early in the epidemic: impact of preprints on global discourse about COVID-19 transmissibility. Lancet Glob Health. 2020 Mar 24. pii: S2214-109X(20)30113-3. doi: 10.1016/S2214-109X(20)30113-3. [Epub ahead of print] PubMed PMID: 32220289.	No appropriate outcome
110: Luo Y, Yin K. Management of pregnant women infected with COVID-19. LancetInfect Dis. 2020 Mar 24. pii: S1473-3099(20)30191-2. doi:10.1016/S1473-3099(20)30191-2. [Epub ahead of print] PubMed PMID: 32220285.	No original article
111: Yu N, Li W, Kang Q, Xiong Z, Wang S, Lin X, Liu Y, Xiao J, Liu H, Deng D,Chen S, Zeng W, Feng L, Wu J. Clinical features and obstetric and neonataloutcomes of pregnant patients with COVID-19 in Wuhan, China: a retrospective,single-centre, descriptive study. Lancet Infect Dis. 2020 Mar 24. pii:S1473-3099(20)30176-6. doi: 10.1016/S1473-3099(20)30176-6. [Epub ahead of print] PubMed PMID: 32220284.	Overlapping data
112: Henry BM, Vikse J. Clinical Characteristics of Covid-19 in China. N Engl JMed. 2020 Mar 27;382. pii: 10.1056/NEJMc2005203#sa4. doi: 10.1056/NEJMc2005203.[Epub ahead of print] PubMed PMID: 32220205.	No original article
113: Wang Y, Zhou CC, Shu R, Zou J. [Oral Health Management of Children duringthe Epidemic Period of Coronavirus Disease 2019]. Sichuan Da Xue Xue Bao Yi XueBan. 2020 Mar;51(2):151-154. doi: 10.12182/20200360101. Review. Chinese. PubMedPMID: 32220180.	No appropriate outcome

114: Qi H, Luo X, Zheng Y, Zhang H, Li J, Zou L, Feng L, Chen D, Shi Y, Tong C,Baker PN. Safe Delivery for COVID-19 Infected Pregnancies. BJOG. 2020 Mar 26.doi: 10.1111/1471-0528.16231. [Epub ahead of print] PubMed PMID: 32219995.	No original article
115: Chen Y, Li Z, Zhang YY, Zhao WH, Yu ZY. Maternal health care managementduring the outbreak of coronavirus disease 2019 (COVID-19). J Med Virol. 2020 Mar26. doi: 10.1002/jmv.25787. [Epub ahead of print] Review. PubMed PMID: 32219871.	No useful data
116: Wang SS, Zhou X, Lin XG, Liu YY, Wu JL, Sharifu LM, Hu XL, Rong ZH, Liu W,Luo XP, Chen Z, Zeng WJ, Chen SH, Ma D, Chen L, Feng L. Experience of ClinicalManagement for Pregnant Women and Newborns with Novel Coronavirus Pneumonia inTongji Hospital, China. Curr Med Sci. 2020 Mar 26. doi:10.1007/s11596-020-2174-4. [Epub ahead of print] PubMed PMID: 32219626.	No original article
117: Michels G, Ochmann U, Cranen R. [Maternity protection-also during the coronacrisis]. Med Klin Intensivmed Notfmed. 2020 Mar 26. doi:10.1007/s00063-020-00677-0. [Epub ahead of print] Review. German. PubMed PMID:32219471; PubMed Central PMCID: PMC7100415.	No appropriate population sample
118: Chen WH, Strych U, Hotez PJ, Bottazzi ME. The SARS-CoV-2 Vaccine Pipeline:an Overview. Curr Trop Med Rep. 2020 Mar 3:1-4. doi: 10.1007/s40475-020-00201-6. [Epub ahead of print] Review. PubMed PMID: 32219057; PubMed Central PMCID:PMC7094941.	No appropriate outcome
119: Voitl P, Niehues T. [Coronavirus disease in children-initial data fromWuhan]. Monatsschr Kinderheilkd. 2020 Mar 24:1-2. doi:10.1007/s00112-020-00907-x. [Epub ahead of print] German. PubMed PMID: 32218613; PubMed Central PMCID: PMC7095346.	No original article

120: Liu M, Song Z, Xiao K. High-Resolution Computed Tomography Manifestations of5 Pediatric Patients With 2019 Novel Coronavirus. J Comput Assist Tomogr. 2020Mar 25. doi: 10.1097/RCT.0000000000001023. [Epub ahead of print] PubMed PMID:32217900	No appropriate population sample
121: Chen G, Wu D, Guo W, Cao Y, Huang D, Wang H, Wang T, Zhang X, Chen H, Yu H, Zhang X, Zhang M, Wu S, Song J, Chen T, Han M, Li S, Luo X, Zhao J, Ning Q.Clinical and immunologic features in severe and moderate Coronavirus Disease2019. J Clin Invest. 2020 Mar 27. pii: 137244. doi: 10.1172/JCI137244. [Epubahead of print] PubMed PMID: 32217835.	No appropriate population sample
122: Chen T, Wu D, Chen H, Yan W, Yang D, Chen G, Ma K, Xu D, Yu H, Wang H, Wang T, Guo W, Chen J, Ding C, Zhang X, Huang J, Han M, Li S, Luo X, Zhao J, Ning Q.Clinical characteristics of 113 deceased patients with coronavirus disease 2019: retrospective study. BMJ. 2020 Mar 26;368:m1091. doi: 10.1136/bmj.m1091. Erratum in: BMJ. 2020 Mar 31;368:m1295. PubMed PMID: 32217556.	No appropriate population sample
123: Dashraath P, Jing Lin Jeslyn W, Mei Xian Karen L, Li Min L, Sarah L, Biswas A, Arjandas Choolani M, Mattar C, Lin SL. Coronavirus Disease 2019 (COVID-19)Pandemic and Pregnancy. Am J Obstet Gynecol. 2020 Mar 23. pii:S0002-9378(20)30343-4. doi: 10.1016/j.ajog.2020.03.021. [Epub ahead of print]PubMed PMID: 32217113.	No original article
127: Atluri S, Manchikanti L, Hirsch JA. Expanded Umbilical Cord Mesenchymal StemCells (UC-MSCs) as a Therapeutic Strategy in Managing Critically Ill COVID-19Patients: The Case for Compassionate Use. Pain Physician. 2020 Mar;23(2):E71-E83.PubMed PMID: 32214286.	No appropriate outcome

128: CDC COVID-19 Response Team. Severe Outcomes Among Patients with Coronavirus Disease 2019 (COVID-19) - United States, February 12-March 16, 2020. MMWR MorbMortal Wkly Rep. 2020 Mar 27;69(12):343-346. doi: 10.15585/mmwr.mm6912e2. PubMed PMID: 32214079.	No appropriate outcome
147: Poon LC, Abramowicz JS, Dall'Asta A, Sande R, Ter Haar G, Maršal K, BrezinkaC, Miloro P, Basseal J, Westerway SC, Abu-Rustum RS, Lees C. ISUOG SafetyCommittee Position Statement: safe performance of obstetric and gynecologicalscans and equipment cleaning in the context of COVID-19. Ultrasound ObstetGynecol. 2020 Mar 23. doi: 10.1002/uog.22027. [Epub ahead of print] PubMed PMID: 32207189	No original article
1: Zeng L, Xia S, Yuan W, Yan K, Xiao F, Shao J, Zhou W. Neonatal Early-OnsetInfection With SARS-CoV-2 in 33 Neonates Born to Mothers With COVID-19 in Wuhan, China. JAMA Pediatr. 2020 Mar 26. doi: 10.1001/jamapediatrics.2020.0878. [Epubahead of print] PubMed PMID: 32215598.	Included
2: Zeng H, Xu C, Fan J, Tang Y, Deng Q, Zhang W, Long X. Antibodies in Infants Born to Mothers With COVID-19 Pneumonia. JAMA. 2020 Mar 26. doi:10.1001/jama.2020.4861. [Epub ahead of print] PubMed PMID: 32215589.	Included
3: Dong L, Tian J, He S, Zhu C, Wang J, Liu C, Yang J. Possible VerticalTransmission of SARS-CoV-2 From an Infected Mother to Her Newborn. JAMA. 2020 Mar26. doi: 10.1001/jama.2020.4621. [Epub ahead of print] PubMed PMID: 32215581.	Included
4: Rasmussen SA, Jamieson DJ. Coronavirus Disease 2019 (COVID-19) and Pregnancy: Responding to a Rapidly Evolving Situation. Obstet Gynecol. 2020 Mar 19. doi:10.1097/AOG.0000000000003873. [Epub ahead of print] PubMed PMID: 32213786.	No original article

5: Kraemer MUG, Yang CH, Gutierrez B, Wu CH, Klein B, Pigott DM; Open COVID-19Data Working Group, du Plessis L, Faria NR, Li R, Hanage WP, Brownstein JS, LayanM, Vespignani A, Tian H, Dye C, Pybus OG, Scarpino SV. The effect of humanmobility and control measures on the COVID-19 epidemic in China. Science. 2020Mar 25. pii: eabb4218. doi: 10.1126/science.abb4218. [Epub ahead of print] PubMedPMID: 32213647.	No appropriate outcome
6: Bhutta ZA, Basnyat B, Saha S, Laxminarayan R. Covid-19 risks and response inSouth Asia. BMJ. 2020 Mar 25;368:m1190. doi: 10.1136/bmj.m1190. PubMed PMID:32213488	No appropriate outcome
7: Koo JR, Cook AR, Park M, Sun Y, Sun H, Lim JT, Tam C, Dickens BL.Interventions to mitigate early spread of SARS-CoV-2 in Singapore: a modellingstudy. Lancet Infect Dis. 2020 Mar 23. pii: S1473-3099(20)30162-6. doi:10.1016/S1473-3099(20)30162-6. [Epub ahead of print] PubMed PMID: 32213332.	No appropriate outcome
8: Le HT, Nguyen LV, Tran DM, Do HT, Tran HT, Le YT, Phan PH. The first infantcase of COVID-19 acquired from a secondary transmission in Vietnam. Lancet Child Adolesc Health. 2020 Mar 23. pii: S2352-4642(20)30091-2. doi:10.1016/S2352-4642(20)30091-2. [Epub ahead of print] PubMed PMID: 32213326.	No appropriate population sample
9: Gan H, Zhang Y, Yuan M, Wu XY, Liu ZR, Liu M, Wu JB, Xu SJ, Gong L, Xu HL, TaoFB. [Epidemiological analysis on 1 052 cases of COVID-19 in epidemic clusters].Zhonghua Liu Xing Bing Xue Za Zhi. 2020 Mar 26;41(5):E027. doi:10.3760/cma.j.cn112338-20200301-00223. [Epub ahead of print] Chinese. PMID: 32213270. PubMed	No appropriate population sample
10: Chen XB, Du SH, Lu JC, Tan XH, Li DR, Yue X, Wang Q, Wang HJ, Qiao DF.Retrospective Analysis of 61 Cases of Children Died of Viral Pneumonia. Fa Yi XueZa Zhi. 2020 Mar 25;36(2). doi: 10.12116/j.issn.1004-5619.2020.02.002.	No appropriate population sample

[Epubahead of print] English, Chinese. PubMed PMID: 32212512.	
11: Szerlip M, Anwaruddin S, Aronow HD, Cohen MG, Daniels MJ, Dehghani P,Drachman DE, Elmariah S, Feldman DN, Garcia S, Giri J, Kaul P, Kapur N, Kumbhani DJ, Meraj PM, Morray B, Nayak KR, Parikh SA, Sakhuja R, Schussler JM, Seto A,Shah B, Swaminathan RV, Zidar DA, Naidu SS. Considerations for CardiacCatheterization Laboratory Procedures During the COVID-19 Pandemic Perspectivesfrom the Society for Cardiovascular Angiography and Interventions Emerging LeaderMentorship (SCAI ELM) Members and Graduates. Catheter Cardiovasc Interv. 2020 Mar25. doi: 10.1002/ccd.28887. [Epub ahead of print] PubMed PMID: 32212409.	No appropriate outcome
12: Brodin P. Why is COVID-19 so mild in children? Acta Paediatr. 2020 Mar 25.doi: 10.1111/apa.15271. [Epub ahead of print] PubMed PMID: 32212348.	No original article
13: Candido DDS, Watts A, Abade L, Kraemer MUG, Pybus OG, Croda J, Oliveira W,Khan K, Sabino EC, Faria NR. Routes for COVID-19 importation in Brazil. J Travel Med. 2020 Mar 23. pii: taaa042. doi: 10.1093/jtm/taaa042. [Epub ahead of print]PubMed PMID: 32211799.	No appropriate outcome
14: Xu B, Gutierrez B, Mekaru S, Sewalk K, Goodwin L, Loskill A, Cohn EL, HswenY, Hill SC, Cobo MM, Zarebski AE, Li S, Wu CH, Hulland E, Morgan JD, Wang L,O'Brien K, Scarpino SV, Brownstein JS, Pybus OG, Pigott DM, Kraemer MUG.Epidemiological data from the COVID-19 outbreak, real-time case information. Sci Data. 2020 Mar 24;7(1):106. doi: 10.1038/s41597-020-0448-0. PubMed PMID:32210236	No appropriate outcome

15: Cron RQ, Chatham WW. The Rheumatologist's Role in Covid-19. J Rheumatol. 2020Mar 24. pii: jrheum.200334. doi: 10.3899/jrheum.200334. [Epub ahead of print]PubMed PMID: 32209661.	No appropriate outcome
16: Unadkat S, Farquhar M. Doctors' wellbeing: self-care during the covid-19pandemic. BMJ. 2020 Mar 24;368:m1150. doi: 10.1136/bmj.m1150. PubMed PMID:32209559	No appropriate population sample
17: An P, Zhang M. Novel coronavirus SARS-CoV-2: familial spread resulting inCOVID-19 pneumonia in a pediatric patient. Diagn Interv Radiol. 2020 Mar 25. doi:10.5152/dir.2020.20157. [Epub ahead of print] PubMed PMID: 32209502.	No appropriate population sample
18: Su L, Ma X, Yu H, Zhang Z, Bian P, Han Y, Sun J, Liu Y, Yang C, Geng J, ZhangZ, Gai Z. The different clinical characteristics of corona virus disease casesbetween children and their families in China - the character of children withCOVID-19. Emerg Microbes Infect. 2020 Dec;9(1):707-713. doi:10.1080/22221751.2020.1744483. PubMed PMID: 32208917.	No appropriate population sample
19: Giwa AL, Desai A, Duca A. Novel 2019 coronavirus SARS-CoV-2 (COVID-19): Anupdated overview for emergency clinicians. Emerg Med Pract. 2020 May1;22(5):1-28. Epub 2020 Mar 24. PubMed PMID: 32207910.	No original article
20: Kang X, Zhang R, He H, Yao Y, Zheng Y, Wen X, Zhu S. [Anesthesia managementin cesarean section for a patient with coronavirus disease 2019]. Zhejiang Da XueXue Bao Yi Xue Ban. 2020 May 25;49(1):0. Chinese. PubMed PMID: 32207592.	No appropriate outcome
21: Moro F, Buonsenso D, Moruzzi MC, Inchingolo R, Smargiassi A, Demi L, LariciAR, Scambia G, Lanzone A, Testa AC. How to perform lung ultrasound in pregnantwomen with suspected COVID-19 infection. Ultrasound Obstet Gynecol. 2020 Mar 24. doi:	No appropriate outcome

10.1002/uog.22028. [Epub ahead of print] PubMed PMID: 32207208.	
22: Lees C. ISUOG Safety Committee Position Statement: safe performance ofobstetric and gynecological scans and equipment cleaning in the context ofCOVID-19. Ultrasound Obstet Gynecol. 2020 Mar 23. doi: 10.1002/uog.22027. [Epubahead of print] PubMed PMID: 32207189.	No appropriate outcome
23: Zheng F, Liao C, Fan QH, Chen HB, Zhao XG, Xie ZG, Li XL, Chen CX, Lu XX, LiuZS, Lu W, Chen CB, Jiao R, Zhang AM, Wang JT, Ding XW, Zeng YG, Cheng LP, HuangQF, Wu J, Luo XC, Wang ZJ, Zhong YY, Bai Y, Wu XY, Jin RM. ClinicalCharacteristics of Children with Coronavirus Disease 2019 in Hubei, China. CurrMed Sci. 2020 Mar 24. doi: 10.1007/s11596-020-2172-6. [Epub ahead of print]PubMed PMID: 32207032.	No appropriate population sample
24: Buckee CO, Balsari S, Chan J, Crosas M, Dominici F, Gasser U, Grad YH,Grenfell B, Halloran ME, Kraemer MUG, Lipsitch M, Metcalf CJE, Meyers LA, PerkinsTA, Santillana M, Scarpino SV, Viboud C, Wesolowski A, Schroeder A. Aggregatedmobility data could help fight COVID-19. Science. 2020 Mar 23. pii: eabb8021.doi: 10.1126/science.abb8021. [Epub ahead of print] PubMed PMID: 32205458.	No appropriate population sample
25: Rimmer A. Covid-19: doctors in final trimester of pregnancy should avoiddirect patient contact. BMJ. 2020 Mar 23;368:m1173. doi: 10.1136/bmj.m1173.PubMed PMID: 32205356.	No appropriate outcome
26: Favalli EG, Ingegnoli F, De Lucia O, Cincinelli G, Cimaz R, Caporali R.COVID-19 infection and rheumatoid arthritis: Faraway, so close! Autoimmun Rev.2020 Mar 20:102523. doi: 10.1016/j.autrev.2020.102523. [Epub ahead of print]Review. PubMed PMID: 32205186.	No appropriate population sample

27: Asperges E, Novati S, Muzzi A, Biscarini S, Sciarra M, Lupi M, Sambo M,Gallazzi I, Peverini M, Lago P, Mojoli F, Perlini S, Bruno R; COVID-19 IRCCS San Matteo Pavia Task Force. Rapid response to COVID-19 outbreak in Northern Italy:how to convert a classic infectious disease ward into a COVID-19 response centre.J Hosp Infect. 2020 Mar 20. pii: S0195-6701(20)30119-5. doi:10.1016/j.jhin.2020.03.020. [Epub ahead of print] PubMed PMID: 32205162.	No appropriate population sample
28: Palacios Cruz M, Santos E, Velázquez Cervantes MA, León Juárez M. COVID-19, aworldwide public health emergency. Rev Clin Esp. 2020 Mar 20. pii:S0014-2565(20)30092-8. doi: 10.1016/j.rce.2020.03.001. [Epub ahead of print]Review. English, Spanish. PubMed PMID: 32204922.	No appropriate population sample
29: Chen Z, DU LZ, Fu JF, Shu Q, Chen ZM, Shi LP, Wang W, Chen SH, Ma XL, Ye S,Sun W, Chen MY, Zhu HH, Huang GL, Luo FX. [Emergency plan for inter-hospitaltransfer of newborns with SARS-CoV-2 infection]. Zhongguo Dang Dai Er Ke Za Zhi. 2020 Mar;22(3):226-230. Chinese. PubMed PMID: 32204758.	No original article
30: Zhang GX, Zhang AM, Huang L, Cheng LY, Liu ZX, Peng XL, Wang HW. [Twin girls infected with SARS-CoV-2]. Zhongguo Dang Dai Er Ke Za Zhi. 2020Mar;22(3):221-225. Chinese. PubMed PMID: 32204757.	No appropriate population sample
31: Zhou Y, Yang GD, Feng K, Huang H, Yun YX, Mou XY, Wang LF. [Clinical featuresand chest CT findings of coronavirus disease 2019 in infants and young children].Zhongguo Dang Dai Er Ke Za Zhi. 2020 Mar;22(3):215-220. Chinese. PubMed PMID:32204756	No appropriate population sample
32: Wang J, Wang D, Chen GC, Tao XW, Zeng LK. [SARS-CoV-2 infection withgastrointestinal symptoms as the first manifestation in a neonate]. Zhongguo DangDai Er Ke Za Zhi. 2020 Mar;22(3):211-214. Chinese. PubMed PMID: 32204755.	Included

33: Medical Association of Chinese People's Liberation Army; Editorial Committee of Chinese Journal of Contemporary Pediatrics; Preparatory Group of PediatricDisaster, Pediatric Society, Chinese Medical Association. [Response plan in theneonatal intensive care unit during epidemic of SARS-CoV-2 infection (2ndEdition)]. Zhongguo Dang Dai Er Ke Za Zhi. 2020 Mar;22(3):205-210. Chinese.	No appropriate outcome
34: Wang LS, Hu XJ, Zhou WH. [An interpretation on perinatal and neonatalmanagement plan for prevention and control of SARS-CoV-2 infection (2ndEdition)]. Zhongguo Dang Dai Er Ke Za Zhi. 2020 Mar;22(3):199-204. Chinese.PubMed PMID: 32204753.	No original article
35: Working Group for the Prevention and Control of Neonatal SARS-CoV-2 Infectionin the Perinatal Period of the Editorial Committee of Chinese Journal ofContemporary Pediatrics. [Perinatal and neonatal management plan for preventionand control of SARS-CoV-2 infection (2nd Edition)]. Zhongguo Dang Dai Er Ke ZaZhi. 2020 Mar;22(3):195-198. Chinese. PubMed PMID: 32204752.	No original article
36: Marraro GA, Spada C. Consideration of the respiratory support strategy ofsevere acute respiratory failure caused by SARS-CoV-2 infection in children.Zhongguo Dang Dai Er Ke Za Zhi. 2020 Mar;22(3):183-194. PubMed PMID: 32204751.	No appropriate population sample
37: Subspecialty Group of Hematology and Oncology, Society of Pediatrics ofHubei. [Standardized management guideline for pediatric wards of hematology andoncology during the epidemic of coronavirus disease 2019]. Zhongguo Dang Dai ErKe Za Zhi. 2020 Mar;22(3):177-182. Chinese. PubMed PMID: 32204750.	No appropriate population sample

100: Li D, Wang D, Dong J, Wang N, Huang H, Xu H, Xia C. False-Negative Results of Real-Time Reverse-Transcriptase Polymerase Chain Reaction for Severe Acute Respiratory Syndrome Coronavirus 2: Role of Deep-Learning-Based CT Diagnosis and Insights from Two Cases. Korean J Radiol. 2020 Apr;21(4):505-508. doi: 10.3348/kjr.2020.0146. Epub 2020 Mar 5. Erratum in: Korean J Radiol. 2020 Mar 20;:. PubMed PMID: 32174053; PubMed Central PMCID: PMC7082661.	No appropriate population sample
1: Ramanathan K, Antognini D, Combes A, Paden M, Zakhary B, Ogino M, MacLaren G, Brodie D, Shekar K. Planning and provision of ECMO services for severe ARDS during the COVID-19 pandemic and other outbreaks of emerging infectious diseases. Lancet Respir Med. 2020 Mar 20. pii: S2213-2600(20)30121-1. doi: 10.1016/S2213-2600(20)30121-1. [Epub ahead of print] Review. PubMed PMID: 32203711	No appropriate outcome
2: Feng S, Shen C, Xia N, Song W, Fan M, Cowling BJ. Rational use of face masks in the COVID-19 pandemic. Lancet Respir Med. 2020 Mar 20. pii: S2213-2600(20)30134-X. doi: 10.1016/S2213-2600(20)30134-X. [Epub ahead of print] PubMed PMID: 32203710.	No appropriate outcome
3: Buonsenso D, Pata D, Chiaretti A. COVID-19 outbreak: less stethoscope, more ultrasound. Lancet Respir Med. 2020 Mar 20. pii: S2213-2600(20)30120-X. doi: 10.1016/S2213-2600(20)30120-X. [Epub ahead of print] PubMed PMID: 32203708. 4: Hu TY, Frieman M, Wolfram J. Insights from nanomedicine into chloroquine	No appropriate outcome
4: Hu TY, Frieman M, Wolfram J. Insights from nanomedicine into chloroquine efficacy against COVID-19. Nat Nanotechnol. 2020 Mar 23. doi: 10.1038/s41565-020-0674-9. [Epub ahead of print] PubMed PMID: 32203437.	No appropriate outcome

5: Ficarra V, Novara G, Abrate A, Bartoletti R, Crestani A, De Nunzio C, Giannarini G, Gregori A, Liguori G, Mirone V, Pavan N, Scarpa RM, Simonato A, Trombetta C, Tubaro A, Porpiglia F; Members of the Research Urology Network (RUN). Urology practice during COVID-19 pandemic. Minerva Urol Nefrol. 2020 Mar 23. doi: 10.23736/S0393-2249.20.03846-1. [Epub ahead of print] PubMed PMID: 32202401	No appropriate population sample
6: Ludvigsson JF. Systematic review of COVID-19 in children show milder cases and a better prognosis than adults. Acta Paediatr. 2020 Mar 23. doi: 10.1111/apa.15270. [Epub ahead of print] PubMed PMID: 32202343.	No original article
7: Britton PN, Marais BJ. Questions raised by COVID-19 case descriptions. J Paediatr Child Health. 2020 Mar 22. doi: 10.1111/jpc.14872. [Epub ahead of print] PubMed PMID: 32202002.	No original article
8: Qian G, Yang N, Ma AHY, Wang L, Li G, Chen X, Chen X. A COVID-19 Transmission within a family cluster by presymptomatic infectors in China. Clin Infect Dis. 2020 Mar 23. pii: ciaa316. doi: 10.1093/cid/ciaa316. [Epub ahead of print] PubMed PMID: 32201889.	No appropriate population sample
9: Colaneri M, Seminari E, Piralla A, Zuccaro V, Filippo AD, Baldanti F, Bruno R, Mondelli MU; COVID19 IRCCS San Matteo Pavia Task Force. Lack of SARS-CoV-2 RNA environmental contamination in a tertiary referral hospital for infectious diseases in Northern Italy. J Hosp Infect. 2020 Mar 19. pii: S0195-6701(20)30117-1. doi: 10.1016/j.jhin.2020.03.018. [Epub ahead of print] PubMed PMID: 32201338.	No appropriate outcome
10: Peyronnet V, Sibiude J, Deruelle P, Huissoud C, Lescure X, Lucet JC, Mandelbrot L, Nisand I, Vayssière C, Yazpandanah Y, Luton D, Picone O. [Infection with SARS-CoV-2 in pregnancy. Information and proposed care. CNGOF.]. Gynecol Obstet Fertil Senol. 2020 Mar 18. pii: S2468-	No original article

7189(20)30110-0. doi: 10.1016/j.gofs.2020.03.014. [Epub ahead of print] French. PubMed PMID: 32199996.	
11: Hong H, Wang Y, Chung HT, Chen CJ. Clinical characteristics of novel coronavirus disease 2019 (COVID-19) in newborns, infants and children. Pediatr Neonatol. 2020 Mar 10. pii: S1875-9572(20)30026-7. doi: 10.1016/j.pedneo.2020.03.001. [Epub ahead of print] PubMed PMID: 32199864.	No original article
12: Lazzerini M, Putoto G. COVID-19 in Italy: momentous decisions and many uncertainties. Lancet Glob Health. 2020 Mar 18. pii: S2214-109X(20)30110-8. doi: 10.1016/S2214-109X(20)30110-8. [Epub ahead of print] PubMed PMID: 32199072.	No appropriate outcome
13: Soldati G, Smargiassi A, Inchingolo R, Buonsenso D, Perrone T, Briganti DF, Perlini S, Torri E, Mariani A, Mossolani EE, Tursi F, Mento F, Demi L. Is there a role for lung ultrasound during the COVID-19 pandemic? J Ultrasound Med. 2020 Mar 20. doi: 10.1002/jum.15284. [Epub ahead of print] PubMed PMID: 32198775.	No appropriate outcome
14: Deng Y, Lei L, Chen Y, Zhang W. The potential added value of FDG PET/CT for COVID-19 pneumonia. Eur J Nucl Med Mol Imaging. 2020 Mar 21. doi: 10.1007/s00259-020-04767-1. [Epub ahead of print] PubMed PMID: 32198615.	No appropriate outcome
15: Duan Y, Zhu HL, Zhou C. Advance of promising targets and agents against 2019-nCoV in China. Drug Discov Today. 2020 Mar 17. pii: S1359-6446(20)30098-2. doi: 10.1016/j.drudis.2020.02.011. [Epub ahead of print] PubMed PMID: 32198066.	No appropriate outcome
16: Wen R, Sun Y, Xing QS. A patient with SARS-CoV-2 infection during pregnancy in Qingdao, China. J Microbiol Immunol Infect. 2020 Mar 10. pii: S1684-1182(20)30061-X. doi:	Included

10.1016/j.jmii.2020.03.004. [Epub ahead of print] PubMed PMID: 32198004.	
17: Yang T, Wang YC, Shen CF, Cheng CM. Point-of-Care RNA-Based Diagnostic Device for COVID-19. Diagnostics (Basel). 2020 Mar 18;10(3). pii: E165. doi: 10.3390/diagnostics10030165. PubMed PMID: 32197339.	No appropriate outcome
18: Kinner SA, Young JT, Snow K, Southalan L, Lopez-Acuña D, Ferreira-Borges C, O'Moore É. Prisons and custodial settings are part of a comprehensive response to COVID-19. Lancet Public Health. 2020 Mar 17. pii: S2468-2667(20)30058-X. doi: 10.1016/S2468-2667(20)30058-X. [Epub ahead of print] PubMed PMID: 32197116.	No appropriate population sample
19: Schmid MB, Fontijn J, Ochsenbein-Kölble N, Berger C, Bassler D. COVID-19 in pregnant women. Lancet Infect Dis. 2020 Mar 17. pii: S1473-3099(20)30175-4. doi: 10.1016/S1473-3099(20)30175-4. [Epub ahead of print] PubMed PMID: 32197098.	No original article
20: Baud D, Giannoni E, Pomar L, Qi X, Nielsen-Saines K, Musso D, Favre G. COVID-19 in pregnant women - Authors' reply. Lancet Infect Dis. 2020 Mar 17. pii: S1473-3099(20)30192-4. doi: 10.1016/S1473-3099(20)30192-4. [Epub ahead of print] PubMed PMID: 32197096.	No original article
21: Yasri S, Wiwanitkit V. Clinical features in pediatric COVID-19. Pediatr Pulmonol. 2020 Mar 20. doi: 10.1002/ppul.24737. [Epub ahead of print] PubMed PMID: 32196995.	No appropriate population sample
22: D'Antiga L. Coronaviruses and immunosuppressed patients. The facts during the third epidemic. Liver Transpl. 2020 Mar 20. doi: 10.1002/lt.25756. [Epub ahead of print] PubMed PMID: 32196933.	No appropriate population sample

23: Dong X, Cao YY, Lu XX, Zhang JJ, Du H, Yan YQ, Akdis CA, Gao YD. Eleven Faces of Coronavirus Disease 2019. Allergy. 2020 Mar 20. doi: 10.1111/all.14289. [Epub ahead of print] PubMed PMID: 32196678.	No appropriate population sample
24: Chen D, Yang H, Cao Y, Cheng W, Duan T, Fan C, Fan S, Feng L, Gao Y, He F, He J, Hu Y, Jiang Y, Li Y, Li J, Li X, Li X, Lin K, Liu C, Liu J, Liu X, Pan X, Pang Q, Pu M, Qi H, Shi C, Sun Y, Sun J, Wang X, Wang Y, Wang Z, Wang Z, Wang C, Wu S, Xin H, Yan J, Zhao Y, Zheng J, Zhou Y, Zou L, Zeng Y, Zhang Y, Guan X, Eppes CS, Fox K, Belfort MA. Expert consensus for managing pregnant women and neonates born to mothers with suspected or confirmed novel coronavirus (COVID-19) infection.Int J Gynaecol Obstet. 2020 Mar 20. doi: 10.1002/ijgo.13146. [Epub ahead of print] PubMed PMID: 32196655.	No original article
25: Buonsenso D, Piano A, Raffaelli F, Bonadia N, de Gaetano Donati K, Franceschi F. Point-of-Care Lung Ultrasound findings in novel coronavirus disease-19 pnemoniae: a case report and potential applications during COVID-19 outbreak. Eur Rev Med Pharmacol Sci. 2020 Mar;24(5):2776-2780. doi: 10.26355/eurrev_202003_20549. PubMed PMID: 32196627.	No appropriate population sample
26: Smith AC, Thomas E, Snoswell CL, Haydon H, Mehrotra A, Clemensen J, Caffery LJ. Telehealth for global emergencies: Implications for coronavirus disease 2019 (COVID-19). J Telemed Telecare. 2020 Mar 20:1357633X20916567. doi: 10.1177/1357633X20916567. [Epub ahead of print] PubMed PMID: 32196391.	No appropriate outcome
27: Zhou D, Dai SM, Tong Q. COVID-19: a recommendation to examine the effect of hydroxychloroquine in preventing infection and progression. J Antimicrob Chemother. 2020 Mar 20. pii: dkaa114. doi: 10.1093/jac/dkaa114. [Epub ahead of print] PubMed PMID: 32196083.	No appropriate outcome

28: Choe YJ, Choi EH. Are We Ready for Coronavirus Disease 2019 Arriving at Schools? J Korean Med Sci. 2020 Mar 23;35(11):e127. doi: 10.3346/jkms.2020.35.e127. PubMed PMID: 32193906.	No appropriate population sample
29: Park JY, Han MS, Park KU, Kim JY, Choi EH. First Pediatric Case of Coronavirus Disease 2019 in Korea. J Korean Med Sci. 2020 Mar 23;35(11):e124. doi: 10.3346/jkms.2020.35.e124. PubMed PMID: 32193905.	No appropriate population sample
30: Sun D, Li H, Lu XX, Xiao H, Ren J, Zhang FR, Liu ZS. Clinical features of severe pediatric patients with coronavirus disease 2019 in Wuhan: a single center's observational study. World J Pediatr. 2020 Mar 19. doi: 10.1007/s12519-020-00354-4. [Epub ahead of print] PubMed PMID: 32193831.	No appropriate population sample
31: Mahase E. Covid-19: schools set to close across UK except for children of health and social care workers. BMJ. 2020 Mar 19;368:m1140. doi: 10.1136/bmj.m1140. PubMed PMID: 32193197.	No appropriate population sample
32: Lippi G, Henry BM. Active smoking is not associated with severity of coronavirus disease 2019 (COVID-19). Eur J Intern Med. 2020 Mar 16. pii: S0953-6205(20)30110-2. doi: 10.1016/j.ejim.2020.03.014. [Epub ahead of print] PubMed PMID: 32192856.	No appropriate population sample
33: Mehta P, McAuley DF, Brown M, Sanchez E, Tattersall RS, Manson JJ; HLH Across Speciality Collaboration, UK. COVID-19: consider cytokine storm syndromes and immunosuppression. Lancet. 2020 Mar 16. pii: S0140-6736(20)30628-0. doi: 10.1016/S0140-6736(20)30628-0. [Epub ahead of print] PubMed PMID: 32192578.	No appropriate outcome
34: COVID-19 National Incident Room Surveillance Team. COVID-19, Australia: Epidemiology Report 7 (Reporting week ending 19:00 AEDT 14 March 2020). Commun Dis Intell (2018). 2020 Mar 19;44. doi:	No appropriate outcome

10.33321/cdi.2020.44.23. PubMed PMID: 32192424	
35: Baglivo M, Baronio M, Natalini G, Beccari T, Chiurazzi P, Fulcheri E, Petralia PP, Michelini S, Fiorentini G, Miggiano GA, Morresi A, Tonini G, Bertelli M. Natural small molecules as inhibitors of coronavirus lipid-dependent attachment to host cells: a possible strategy for reducing SARS-COV-2 infectivity? Acta Biomed. 2020 Mar 19;91(1):161-164. doi: 10.23750/abm.v91i1.9402. PubMed PMID: 32191676.	No appropriate outcome
36: Sahu KK, Lal A, Mishra AK. COVID-2019 and Pregnancy: A plea for transparent reporting of all cases. Acta Obstet Gynecol Scand. 2020 Mar 19. doi: 10.1111/aogs.13850. [Epub ahead of print] PubMed PMID: 32191350.	No original article
37: Rimmer A. Covid-19: pregnant doctors should speak to occupational health, say experts. BMJ. 2020 Mar 18;368:m1104. doi: 10.1136/bmj.m1104. PubMed PMID: 32188586	No appropriate outcome
38: Li Y, Guo F, Cao Y, Li L, Guo Y. Insight into COVID-2019 for pediatricians. Pediatr Pulmonol. 2020 Mar 18. doi: 10.1002/ppul.24734. [Epub ahead of print] PubMed PMID: 32187887.	No appropriate population sample
39: Cossarizza A, De Biasi S, Guaraldi G, Girardis M, Mussini C; Modena Covid-19 Working Group (MoCo19)#. SARS-CoV-2, the Virus that Causes COVID-19: Cytometry and the New Challenge for Global Health. Cytometry A. 2020 Mar 18. doi: 10.1002/cyto.a.24002. [Epub ahead of print] PubMed PMID: 32187834.	No appropriate outcome

40: Lu X, Zhang L, Du H, Zhang J, Li YY, Qu J, Zhang W, Wang Y, Bao S, Li Y, Wu C, Liu H, Liu D, Shao J, Peng X, Yang Y, Liu Z, Xiang Y, Zhang F, Silva RM, Pinkerton KE, Shen K, Xiao H, Xu S, Wong GWK; Chinese Pediatric Novel Coronavirus Study Team. SARS-CoV-2 Infection in Children. N Engl J Med. 2020 Mar 18. doi: 10.1056/NEJMc2005073. [Epub ahead of print] PubMed PMID: 32187458.	No original article
41: Liu D, Li L, Wu X, Zheng D, Wang J, Yang L, Zheng C. Pregnancy and Perinatal Outcomes of Women With Coronavirus Disease (COVID-19) Pneumonia: A Preliminary Analysis. AJR Am J Roentgenol. 2020 Mar 18:1-6. doi: 10.2214/AJR.20.23072. [Epub ahead of print] PubMed PMID: 32186894.	Included
42: Rashidi Fakari F, Simbar M. Coronavirus Pandemic and Worries during Pregnancy; a Letter to Editor. Arch Acad Emerg Med. 2020 Mar 16;8(1):e21. eCollection 2020. PubMed PMID: 32185371; PubMed Central PMCID: PMC7075675.	No original article
43: Mizumoto K, Kagaya K, Zarebski A, Chowell G. Estimating the asymptomatic proportion of coronavirus disease 2019 (COVID-19) cases on board the Diamond Princess cruise ship, Yokohama, Japan, 2020. Euro Surveill. 2020 Mar;25(10). doi: 10.2807/1560-7917.ES.2020.25.10.2000180. PubMed PMID: 32183930.	No appropriate population sample
44: Fan C, Lei D, Fang C, Li C, Wang M, Liu Y, Bao Y, Sun Y, Huang J, Guo Y, Yu Y, Wang S. Perinatal Transmission of COVID-19 Associated SARS-CoV-2: Should We Worry? Clin Infect Dis. 2020 Mar 17. pii: ciaa226. doi: 10.1093/cid/ciaa226. [Epub ahead of print] PubMed PMID: 32182347.	Included
45: Gao Y, Li T, Han M, Li X, Wu D, Xu Y, Zhu Y, Liu Y, Wang X, Wang L. Diagnostic Utility of Clinical Laboratory Data Determinations for Patients with the Severe COVID-19. J Med Virol. 2020 Mar 17. doi:	No appropriate outcome

10.1002/jmv.25770. [Epub ahead of print] PubMed PMID: 32181911.	
46: Jiao J. Under the epidemic situation of COVID-19, should special attention to pregnant women be given? J Med Virol. 2020 Mar 17. doi: 10.1002/jmv.25771. [Epub ahead of print] PubMed PMID: 32181904.	No original article
47: Schwartz DA. An Analysis of 38 Pregnant Women with COVID-19, Their Newborn Infants, and Maternal-Fetal Transmission of SARS-CoV-2: Maternal Coronavirus Infections and Pregnancy Outcomes. Arch Pathol Lab Med. 2020 Mar 17. doi: 10.5858/arpa.2020-0901-SA. [Epub ahead of print] PubMed PMID: 32180426.	No original article
48: Mullins E, Evans D, Viner RM, O'Brien P, Morris E. Coronavirus in pregnancy and delivery: rapid review. Ultrasound Obstet Gynecol. 2020 Mar 17. doi: 10.1002/uog.22014. [Epub ahead of print] Review. PubMed PMID: 32180292.	No original article
49: Chen R, Zhang Y, Huang L, Cheng BH, Xia ZY, Meng QT. Safety and efficacy of different anesthetic regimens for parturients with COVID-19 undergoing Cesarean delivery: a case series of 17 patients. Can J Anaesth. 2020 Mar 16. doi: 10.1007/s12630-020-01630-7. [Epub ahead of print] PubMed PMID: 32180175.	Included
50: Ji LN, Chao S, Wang YJ, Li XJ, Mu XD, Lin MG, Jiang RM. Clinical features of pediatric patients with COVID-19: a report of two family cluster cases. World J Pediatr. 2020 Mar 16. doi: 10.1007/s12519-020-00356-2. [Epub ahead of print] PubMed PMID: 32180140.	No appropriate population sample

51: Wang Y, Liu Y, Liu L, Wang X, Luo N, Ling L. Clinical outcome of 55 asymptomatic cases at the time of hospital admission infected with SARS-Coronavirus-2 in Shenzhen, China. J Infect Dis. 2020 Mar 17. pii: jiaa119. doi: 10.1093/infdis/jiaa119. [Epub ahead of print] PubMed PMID: 32179910.	No appropriate population sample
52: Cui Y, Tian M, Huang D, Wang X, Huang Y, Fan L, Wang L, Chen Y, Liu W, Zhang K, Wu Y, Yang Z, Tao J, Feng J, Liu K, Ye X, Wang R, Zhang X, Zha Y. A 55-Day-Old Female Infant infected with COVID 19: presenting with pneumonia, liver injury, and heart damage. J Infect Dis. 2020 Mar 17. pii: jiaa113. doi: 10.1093/infdis/jiaa113. [Epub ahead of print] PubMed PMID: 32179908.	No appropriate population sample
53: Dong Y, Mo X, Hu Y, Qi X, Jiang F, Jiang Z, Tong S. Epidemiological Characteristics of 2143 Pediatric Patients With 2019 Coronavirus Disease in China. Pediatrics. 2020 Mar 16. pii: e20200702. doi: 10.1542/peds.2020-0702. [Epub ahead of print] PubMed PMID: 32179660.	No appropriate population sample
54: COVID-19 in Children: Initial Characterization of the Pediatric Disease. Pediatrics. 2020 Mar 16. pii: e20200834. doi: 10.1542/peds.2020-0834. [Epub ahead of print] PubMed PMID: 32179659.	No appropriate population sample
55: Rodriguez-Morales AJ, Cardona-Ospina JA, Gutiérrez-Ocampo E, Villamizar-Peña R, Holguin-Rivera Y, Escalera-Antezana JP, Alvarado-Arnez LE, Bonilla-Aldana DK, Franco-Paredes C, Henao-Martinez AF, Paniz-Mondolfi A, Lagos-Grisales GJ, Ramírez-Vallejo E, Suárez JA, Zambrano LI, Villamil-Gómez WE, Balbin-Ramon GJ, Rabaan AA, Harapan H, Dhama K, Nishiura H, Kataoka H, Ahmad T, Sah R; Latin American Network of Coronavirus Disease 2019-COVID-19 Research (LANCOVID-19).Electronic address: https://www.lancovid.org. Clinical, laboratory and imaging features of COVID-19: A systematic review and meta-analysis. Travel Med Infect Dis. 2020 Mar 13:101623. doi: 10.1016/j.tmaid.2020.101623. [Epub ahead of print] Review. PubMed PMID: 32179124.	No original article

56: Lippi G, Plebani M, Michael Henry B. Thrombocytopenia is associated with severe coronavirus disease 2019 (COVID-19) infections: A meta-analysis. Clin Chim Acta. 2020 Mar 13. pii: S0009-8981(20)30124-8. doi: 10.1016/j.cca.2020.03.022. [Epub ahead of print] PubMed PMID: 32178975.	No appropriate population sample
57: Zhao S, Ling K, Yan H, Zhong L, Peng X, Yao S, Huang J, Chen X. Anesthetic Management of Patients With Suspected or Confirmed 2019 Novel Coronavirus Infection During Emergency Procedures. J Cardiothorac Vasc Anesth. 2020 Feb 28. pii: S1053-0770(20)30197-X. doi: 10.1053/j.jvca.2020.02.039. [Epub ahead of print] PubMed PMID: 32178954.	No appropriate population sample
58: Henry BM. COVID-19, ECMO, and lymphopenia: a word of caution. Lancet Respir Med. 2020 Mar 13. pii: S2213-2600(20)30119-3. doi: 10.1016/S2213-2600(20)30119-3. [Epub ahead of print] PubMed PMID: 32178774.	No appropriate outcome
59: Cimolai N. Potentially repurposing adamantanes for COVID-19. J Med Virol. 2020 Mar 16. doi: 10.1002/jmv.25752. [Epub ahead of print] PubMed PMID: 32176361.	No appropriate outcome
60: Yang S, Cao P, Du P, Wu Z, Zhuang Z, Yang L, Yu X, Zhou Q, Feng X, Wang X, Li W, Liu E, Chen J, Chen Y, He D. Early estimation of the case fatality rate of COVID-19 in mainland China: a data-driven analysis. Ann Transl Med. 2020 Feb;8(4):128. doi: 10.21037/atm.2020.02.66. PubMed PMID: 32175421; PubMed Central PMCID: PMC7049028.	No appropriate outcome
61: Zhang J, Peng P, Li X, Zha YF, Zhang GN, Zhang Y, Xiang Y. [Management strategies for patients with gynecological malignancies during the outbreak of COVID19]. Zhonghua Fu Chan Ke Za Zhi. 2020 Mar 16;55(0):E011. doi: 10.3760/cma.j.cn112141-20200302-00168. [Epub ahead of print] Chinese. PubMed PMID: 32174096.	No appropriate population sample

62: Korean Society of Infectious Diseases; Korean Society of Pediatric Infectious Diseases; Korean Society of Epidemiology; Korean Society for Antimicrobial Therapy; Korean Society for Healthcare-associated Infection Control and Prevention; Korea Centers for Disease Control and Prevention. Report on the Epidemiological Features of Coronavirus Disease 2019 (COVID-19) Outbreak in the Republic of Korea from January 19 to March 2, 2020. J Korean Med Sci. 2020 Mar16;35(10):e112. doi: 10.3346/jkms.2020.35.e112. PubMed PMID: 32174069; PubMed Central PMCID: PMC7073313.	No appropriate outcome
63: Lai CC, Liu YH, Wang CY, Wang YH, Hsueh SC, Yen MY, Ko WC, Hsueh PR. Asymptomatic carrier state, acute respiratory disease, and pneumonia due to severe acute respiratory syndrome coronavirus 2 (SARS-CoV-2): Facts and myths. J Microbiol Immunol Infect. 2020 Mar 4. pii: S1684-1182(20)30040-2. doi: 10.1016/j.jmii.2020.02.012. [Epub ahead of print] Review. PubMed PMID: 32173241.	No appropriate population sample
64: Calvo C, García López-Hortelano M, de Carlos Vicente JC, Vázquez Martínez JL; Grupo de trabajo de la Asociación Española de Pediatría para el brote de infección por Coronavirus, colaboradores con el Ministerio de Sanidad; Miembros del Grupo de Expertos de la AEP. [Recommendations on the clinical management of the COVID-19 infection by the «new coronavirus» SARS-CoV2. Spanish Paediatric Association working group]. An Pediatr (Barc). 2020 Mar 12. pii:S1695-4033(20)30076-X. doi: 10.1016/j.anpedi.2020.02.001. [Epub ahead of print] Spanish. PubMed PMID: 32173188.	No original article
65: Henry BM, Lippi G, Plebani M. Laboratory abnormalities in children with novel coronavirus disease 2019. Clin Chem Lab Med. 2020 Mar 16. pii: /j/cclm.ahead-of-print/cclm-2020-0272/cclm-2020-0272.xml. doi: 10.1515/cclm-2020-0272. [Epub ahead of print] PubMed PMID: 32172227.	No original article

66: Liu H, Liu F, Li J, Zhang T, Wang D, Lan W. Clinical and CT Imaging Features of the COVID-19 Pneumonia: Focus on Pregnant Women and Children. J Infect. 2020 Mar 11. pii: S0163-4453(20)30118-3. doi: 10.1016/j.jinf.2020.03.007. [Epub ahead of print] PubMed PMID: 32171865.	Overlapping data
67: Baud D, Qi X, Nielsen-Saines K, Musso D, Pomar L, Favre G. Real estimates of mortality following COVID-19 infection. Lancet Infect Dis. 2020 Mar 12. pii: S1473-3099(20)30195-X. doi: 10.1016/S1473-3099(20)30195-X. [Epub ahead of print] PubMed PMID: 32171390.	No appropriate population sample
68: Thompson LA, Rasmussen SA. What Does the Coronavirus Disease 2019 (COVID-19) Mean for Families? JAMA Pediatr. 2020 Mar 13. doi: 10.1001/jamapediatrics.2020.0828. [Epub ahead of print] PubMed PMID: 32167533.	No appropriate outcome
69: Wu C, Chen X, Cai Y, Xia J, Zhou X, Xu S, Huang H, Zhang L, Zhou X, Du C, Zhang Y, Song J, Wang S, Chao Y, Yang Z, Xu J, Zhou X, Chen D, Xiong W, Xu L, Zhou F, Jiang J, Bai C, Zheng J, Song Y. Risk Factors Associated With Acute Respiratory Distress Syndrome and Death in Patients With Coronavirus Disease 2019 Pneumonia in Wuhan, China. JAMA Intern Med. 2020 Mar 13. doi: 10.1001/jamainternmed.2020.0994. [Epub ahead of print] PubMed PMID: 32167524;PubMed Central PMCID: PMC7070509.	No appropriate population sample
70: Singhal T. A Review of Coronavirus Disease-2019 (COVID-19). Indian J Pediatr. 2020 Apr;87(4):281-286. doi: 10.1007/s12098-020-03263-6. Epub 2020 Mar 13. Review. PubMed PMID: 32166607.	No original article
71: Ng OT, Marimuthu K, Chia PY, Koh V, Chiew CJ, De Wang L, Young BE, Chan M, Vasoo S, Ling LM, Lye DC, Kam KQ, Thoon KC, Kurupatham L, Said Z, Goh E, Low C, Lim SK, Raj P, Oh O, Koh VTJ, Poh C, Mak TM, Cui L, Cook AR, Lin RTP, Leo YS, Lee VJM. SARS-CoV-2 Infection among Travelers Returning from Wuhan, China. N Engl J Med.	No appropriate population sample

2020 Mar 12. doi: 10.1056/NEJMc2003100. [Epub ahead of print] PubMed PMID: 32163698	
72: Liu W, Zhang Q, Chen J, Xiang R, Song H, Shu S, Chen L, Liang L, Zhou J, You L, Wu P, Zhang B, Lu Y, Xia L, Huang L, Yang Y, Liu F, Semple MG, Cowling BJ, Lan K, Sun Z, Yu H, Liu Y. Detection of Covid-19 in Children in Early January 2020 in Wuhan, China. N Engl J Med. 2020 Mar 12. doi: 10.1056/NEJMc2003717. [Epub ahead of print] PubMed PMID: 32163697.	No original article
73: Li W, Cui H, Li K, Fang Y, Li S. Chest computed tomography in children with COVID-19 respiratory infection. Pediatr Radiol. 2020 Mar 11. doi: 10.1007/s00247-020-04656-7. [Epub ahead of print] PubMed PMID: 32162081.	No appropriate population sample
74: Wang S, Guo L, Chen L, Liu W, Cao Y, Zhang J, Feng L. A case report of neonatal COVID-19 infection in China. Clin Infect Dis. 2020 Mar 12. pii: ciaa225. doi: 10.1093/cid/ciaa225. [Epub ahead of print] PubMed PMID: 32161941.	Overlapping data
75: Stower H. Lack of maternal-fetal SARS-CoV-2 transmission. Nat Med. 2020 Mar;26(3):312. doi: 10.1038/s41591-020-0810-y. PubMed PMID: 32161408.	No original article
76: Poon LC, Yang H, Lee JCS, Copel JA, Leung TY, Zhang Y, Chen D, Prefumo F. ISUOG Interim Guidance on 2019 novel coronavirus infection during pregnancy and puerperium: information for healthcare professionals. Ultrasound Obstet Gynecol. 2020 Mar 11. doi: 10.1002/uog.22013. [Epub ahead of print] PubMed PMID: 32160345.	No original article

77: Spiteri G, Fielding J, Diercke M, Campese C, Enouf V, Gaymard A, Bella A, Sognamiglio P, Sierra Moros MJ, Riutort AN, Demina YV, Mahieu R, Broas M, Bengnér M, Buda S, Schilling J, Filleul L, Lepoutre A, Saura C, Mailles A, Levy-Bruhl D, Coignard B, Bernard-Stoecklin S, Behillil S, van der Werf S, Valette M, Lina B, Riccardo F, Nicastri E, Casas I, Larrauri A, Salom Castell M, Pozo F, Maksyutov RA, Martin C, Van Ranst M, Bossuyt N, Siira L, Sane J, Tegmark-Wisell K, PalmérusM, Broberg EK, Beauté J, Jorgensen P, Bundle N, Pereyaslov D, Adlhoch C, Pukkila J, Pebody R, Olsen S, Ciancio BC. First cases of coronavirus disease 2019 (COVID-19) in the WHO European Region, 24 January to 21 February 2020. Euro Surveill. 2020 Mar;25(9). doi: 10.2807/1560-7917.ES.2020.25.9.2000178. PubMed PMID: 32156327; PubMed Central PMCID: PMC7068164.	No appropriate population sample
78: COVID-19 National Incident Room Surveillance Team. COVID-19, Australia: Epidemiology Report 6 (Reporting week ending 19:00 AEDT 7 March 2020). Commun Dis Intell (2018). 2020 Mar 11;44. doi: 10.33321/cdi.2020.44.21. PubMed PMID: 32156224	No appropriate outcome
79: Li W, Zhou Q, Tang Y, Ren L, Yu X, Li Q, Liu E, Chen Y; COVID-19 evidence and recommendations working group. Protocol for the development of a rapid advice guideline for prevention, management and care of children with 2019 novel coronavirus infection. Ann Palliat Med. 2020 Feb 24. pii: apm.2020.02.33. doi: 10.21037/apm.2020.02.33. [Epub ahead of print] PubMed PMID: 32156144.	No original article
80: Wang C, Pan R, Wan X, Tan Y, Xu L, Ho CS, Ho RC. Immediate Psychological Responses and Associated Factors during the Initial Stage of the 2019 Coronavirus Disease (COVID-19) Epidemic among the General Population in China. Int J Environ Res Public Health. 2020 Mar 6;17(5). pii: E1729. doi: 10.3390/ijerph17051729. PubMed PMID: 32155789.	No appropriate outcome

81: Hageman JR. The Coronavirus Disease 2019 (COVID-19). Pediatr Ann. 2020 Mar 1;49(3):e99-e100. doi: 10.3928/19382359-20200219-01. PubMed PMID: 32155273.	No original article
82: Wang L, Shi Y, Xiao T, Fu J, Feng X, Mu D, Feng Q, Hei M, Hu X, Li Z, Lu G, Tang Z, Wang Y, Wang C, Xia S, Xu J, Yang Y, Yang J, Zeng M, Zheng J, Zhou W, Zhou X, Zhou X, Du L, Lee SK, Zhou W; Working Committee on Perinatal and Neonatal Management for the Prevention and Control of the 2019 Novel Coronavirus Infection. Chinese expert consensus on the perinatal and neonatal management for the prevention and control of the 2019 novel coronavirus infection (Firstedition). Ann Transl Med. 2020 Feb;8(3):47. doi: 10.21037/atm.2020.02.20. Review. PubMed PMID: 32154287; PubMed Central PMCID: PMC7036629.	No original article
83: Zhu H, Wang L, Fang C, Peng S, Zhang L, Chang G, Xia S, Zhou W. Clinical analysis of 10 neonates born to mothers with 2019-nCoV pneumonia. Transl Pediatr. 2020 Feb;9(1):51-60. doi: 10.21037/tp.2020.02.06. PubMed PMID: 32154135; PubMed Central PMCID: PMC7036645.	Included
84: Caselli D, Aricò M. 2019-nCoV: Polite with children! Pediatr Rep. 2020 Feb 11;12(1):8495. doi: 10.4081/pr.2020.8495. eCollection 2020 Feb 11. PubMed PMID: 32153742; PubMed Central PMCID: PMC7036705.	No original article
85: Chen H, Guo J, Wang C, Luo F, Yu X, Zhang W, Li J, Zhao D, Xu D, Gong Q, Liao J, Yang H, Hou W, Zhang Y. Clinical characteristics and intrauterine vertical transmission potential of COVID-19 infection in nine pregnant women: a retrospective review of medical records. Lancet. 2020 Mar 7;395(10226):809-815. doi: 10.1016/S0140-6736(20)30360-3. Epub 2020 Feb 12. PubMed PMID: 32151335.	Included

86: Qiao J. What are the risks of COVID-19 infection in pregnant women? Lancet. 2020 Mar 7;395(10226):760-762. doi: 10.1016/S0140-6736(20)30365-2. Epub 2020 Feb 12. PubMed PMID: 32151334.	No original article
87: De Luca D. Managing neonates with respiratory failure due to SARS-CoV-2. Lancet Child Adolesc Health. 2020 Apr;4(4):e8. doi: 10.1016/S2352-4642(20)30073-0. Epub 2020 Mar 6. PubMed PMID: 32151320.	No original article
88: Wang J, Shi Y. Managing neonates with respiratory failure due to SARS-CoV-2 - Authors' reply. Lancet Child Adolesc Health. 2020 Apr;4(4):e9. doi: 10.1016/S2352-4642(20)30072-9. Epub 2020 Mar 6. PubMed PMID: 32151318.	No original article
89: Tang A, Tong ZD, Wang HL, Dai YX, Li KF, Liu JN, Wu WJ, Yuan C, Yu ML, Li P, Yan JB. Detection of Novel Coronavirus by RT-PCR in Stool Specimen from Asymptomatic Child, China. Emerg Infect Dis. 2020 Jun 17;26(6). doi: 10.3201/eid2606.200301. [Epub ahead of print] PubMed PMID: 32150527.	No appropriate population sample
90: Chen Z, Xiong H, Li JX, Li H, Tao F, Yang YT, Wu B, Tang W, Teng JX, Fu Q, Yang L. [COVID-19 with post-chemotherapy agranulocytosis in childhood acute leukemia: a case report]. Zhonghua Xue Ye Xue Za Zhi. 2020 Mar 9;41(0):E004. doi: 10.3760/cma.j.issn.0253-2727.2020.0004. [Epub ahead of print] Chinese. PubMed PMID: 32149486.	No appropriate population sample
91: Ko WC, Rolain JM, Lee NY, Chen PL, Huang CT, Lee PI, Hsueh PR. Arguments in favour of remdesivir for treating SARS-CoV-2 infections. Int J Antimicrob Agents. 2020 Mar 6:105933. doi: 10.1016/j.ijantimicag.2020.105933. [Epub ahead of print] PubMed PMID: 32147516.	No appropriate population sample

92: Lee PI, Hu YL, Chen PY, Huang YC, Hsueh PR. Are children less susceptible to COVID-19? J Microbiol Immunol Infect. 2020 Feb 25. pii: S1684-1182(20)30039-6. doi: 10.1016/j.jmii.2020.02.011. [Epub ahead of print] PubMed PMID: 32147409.	No appropriate population sample
93: Hu Z, Song C, Xu C, Jin G, Chen Y, Xu X, Ma H, Chen W, Lin Y, Zheng Y, Wang J, Hu Z, Yi Y, Shen H. Clinical characteristics of 24 asymptomatic infections with COVID-19 screened among close contacts in Nanjing, China. Sci China Life Sci. 2020 Mar 4. doi: 10.1007/s11427-020-1661-4. [Epub ahead of print] PubMed PMID: 32146694.	No appropriate population sample
94: Porcheddu R, Serra C, Kelvin D, Kelvin N, Rubino S. Similarity in Case Fatality Rates (CFR) of COVID-19/SARS-COV-2 in Italy and China. J Infect Dev Ctries. 2020 Feb 29;14(2):125-128. doi: 10.3855/jidc.12600. PubMed PMID: 32146445	No appropriate population sample
95: Tao FB. [Healing the schism between public health and medicine, promoting the integration of prevention and treatment]. Zhonghua Yu Fang Yi Xue Za Zhi. 2020 Mar 8;54(0):E024. doi: 10.3760/cma.j.cn112150-20200304-00246. [Epub ahead of print] Chinese. PubMed PMID: 32145718.	No appropriate outcome
96: Zhang L, Jiang Y, Wei M, Cheng BH, Zhou XC, Li J, Tian JH, Dong L, Hu RH. [Analysis of the pregnancy outcomes in pregnant women with COVID-19 in Hubei Province]. Zhonghua Fu Chan Ke Za Zhi. 2020 Mar 7;55(0):E009. doi: 10.3760/cma.j.cn112141-20200218-00111. [Epub ahead of print] Chinese. PubMed PMID: 32145714.	Overlapping data
97: He Y, Wang Z, Li F, Shi Y. Public health might be endangered by possible prolonged discharge of SARS-CoV-2 in stool. J Infect. 2020 Mar 5. pii: S0163-4453(20)30111-0. doi: 10.1016/j.jinf.2020.02.031. [Epub ahead of print] PubMed PMID: 32145217.	No appropriate population sample

98: Liu Y, Chen H, Tang K, Guo Y. Clinical manifestations and outcome of SARS-CoV-2 infection during pregnancy. J Infect. 2020 Mar 4. pii: S0163-4453(20)30109-2. doi: 10.1016/j.jinf.2020.02.028. [Epub ahead of print] PubMed PMID: 32145216.	Included
99: Wang G, Zhang Y, Zhao J, Zhang J, Jiang F. Mitigate the effects of home confinement on children during the COVID-19 outbreak. Lancet. 2020 Mar 21;395(10228):945-947. doi: 10.1016/S0140-6736(20)30547-X. Epub 2020 Mar 4. PubMed PMID: 32145186.	No appropriate population sample
100: Lin M, Beliavsky A, Katz K, Powis JE, Ng W, Williams V, Science M, Groves H, Muller MP, Vaisman A, Hota S, Johnstone J, Leis JA. What can early Canadian experience screening for COVID-19 teach us about how to prepare for a pandemic? CMAJ. 2020 Mar 6. pii: cmaj.200305. doi: 10.1503/cmaj.200305. [Epub ahead of print] PubMed PMID: 32144097.	No appropriate outcome
101: Yang Y, Peng F, Wang R, Guan K, Jiang T, Xu G, Sun J, Chang C. The deadly coronaviruses: The 2003 SARS pandemic and the 2020 novel coronavirus epidemic in China. J Autoimmun. 2020 Mar 3:102434. doi: 10.1016/j.jaut.2020.102434. [Epub ahead of print] Review. PubMed PMID: 32143990.	No appropriate outcome
102: Lee IK, Wang CC, Lin MC, Kung CT, Lan KC, Lee CT. Effective strategies to prevent coronavirus disease-2019 (COVID-19) outbreak in hospital. J Hosp Infect. 2020 Mar 3. pii: S0195-6701(20)30098-0. doi: 10.1016/j.jhin.2020.02.022. [Epub ahead of print] PubMed PMID: 32142886.	No appropriate population sample
103: Favre G, Pomar L, Qi X, Nielsen-Saines K, Musso D, Baud D. Guidelines for pregnant women with suspected SARS-CoV-2 infection. Lancet Infect Dis. 2020 Mar 3. pii: S1473-3099(20)30157-2. doi: 10.1016/S1473-3099(20)30157-2. [Epub ahead of print] PubMed PMID: 32142639.	No original article

104: Han Y, Yang H. The transmission and diagnosis of 2019 novel coronavirus infection disease (COVID-19): A Chinese perspective. J Med Virol. 2020 Mar 6. doi: 10.1002/jmv.25749. [Epub ahead of print] PubMed PMID: 32141619.	No appropriate outcome
105: Liang H, Acharya G. Novel corona virus disease (COVID-19) in pregnancy: What clinical recommendations to follow? Acta Obstet Gynecol Scand. 2020 Apr;99(4):439-442. doi: 10.1111/aogs.13836. Epub 2020 Mar 5. PubMed PMID: 32141062	No original article
106: Cao Q, Chen YC, Chen CL, Chiu CH. SARS-CoV-2 infection in children: Transmission dynamics and clinical characteristics. J Formos Med Assoc. 2020 Mar;119(3):670-673. doi: 10.1016/j.jfma.2020.02.009. Epub 2020 Mar 2. PubMed PMID: 32139299.	No original article
107: Zhang YH, Lin DJ, Xiao MF, Wang JC, Wei Y, Lei ZX, Zeng ZQ, Li L, Li HA, Xiang W. [2019 novel coronavirus infection in a three-month-old baby]. Zhonghua Er Ke Za Zhi. 2020 Mar 2;58(3):182-184. doi: 10.3760/cma.j.issn.0578-1310.2020.03.004. Chinese. PubMed PMID: 32135587.	No appropriate population sample
108: Chen F, Liu ZS, Zhang FR, Xiong RH, Chen Y, Cheng XF, Wang WY, Ren J. [First case of severe childhood novel coronavirus pneumonia in China]. Zhonghua Er Ke Za Zhi. 2020 Mar 2;58(3):179-182. doi: 10.3760/cma.j.issn.0578-1310.2020.03.003. Chinese. PubMed PMID: 32135586.	No appropriate population sample
109: Subspecialty Group of Gastroenterology, the Society of Pediatrics, Chinese Medical Association. [Prevention and control program on 2019 novel coronavirus infection in children's digestive endoscopy center]. Zhonghua Er Ke Za Zhi. 2020 Mar 2;58(3):175-178. doi: 10.3760/cma.j.issn.0578-1310.2020.03.002. Chinese. PubMed PMID: 32135585.	No appropriate population sample

110: Society of Pediatrics, Chinese Medical Association; Editorial Board, Chinese Journal of Pediatrics. [Recommendations for the diagnosis, prevention and control of the 2019 novel coronavirus infection in children (first interim edition)]. Zhonghua Er Ke Za Zhi. 2020 Mar 2;58(3):169-174. doi: 10.3760/cma.j.issn.0578-1310.2020.03.001. Chinese. PubMed PMID: 32135584.	No original article
111: Li Y, Zhao R, Zheng S, Chen X, Wang J, Sheng X, Zhou J, Cai H, Fang Q, Yu F, Fan J, Xu K, Chen Y, Sheng J. Lack of Vertical Transmission of Severe Acute Respiratory Syndrome Coronavirus 2, China. Emerg Infect Dis. 2020 Jun 17;26(6). doi: 10.3201/eid2606.200287. [Epub ahead of print] PubMed PMID: 32134381.	Included
112: Xia W, Shao J, Guo Y, Peng X, Li Z, Hu D. Clinical and CT features in pediatric patients with COVID-19 infection: Different points from adults. Pediatr Pulmonol. 2020 Mar 5. doi: 10.1002/ppul.24718. [Epub ahead of print] PubMed PMID: 32134205	No appropriate outcome
113: Wang Y, Wang Y, Chen Y, Qin Q. Unique epidemiological and clinical features of the emerging 2019 novel coronavirus pneumonia (COVID-19) implicate special control measures. J Med Virol. 2020 Mar 5. doi: 10.1002/jmv.25748. [Epub ahead of print] Review. PubMed PMID: 32134116.	No appropriate outcome
114: Liu W, Zhu HL, Duan Y. Effective Chemicals against Novel Coronavirus (COVID-19) in China. Curr Top Med Chem. 2020 Mar 5. doi: 10.2174/1568026620999200305145032. [Epub ahead of print] PubMed PMID: 32133962.	No appropriate outcome
115: Chua M, Lee J, Sulaiman S, Tan HK. From the frontline of COVID-19 - How prepared are we as obstetricians: a commentary. BJOG. 2020 Mar 4. doi: 10.1111/1471-0528.16192. [Epub ahead of print] PubMed PMID: 32131142.	No appropriate population sample

116: Peng X, Xu X, Li Y, Cheng L, Zhou X, Ren B. Transmission routes of 2019-nCoV and controls in dental practice. Int J Oral Sci. 2020 Mar 3;12(1):9. doi: 10.1038/s41368-020-0075-9. Review. PubMed PMID: 32127517; PubMed Central PMCID: PMC7054527.	No appropriate population sample
117: Rodriguez-Morales AJ, Gallego V, Escalera-Antezana JP, Méndez CA, Zambrano LI, Franco-Paredes C, Suárez JA, Rodriguez-Enciso HD, Balbin-Ramon GJ, Savio-Larriera E, Risquez A, Cimerman S. COVID-19 in Latin America: The implications of the first confirmed case in Brazil. Travel Med Infect Dis. 2020 Feb 29:101613. doi: 10.1016/j.tmaid.2020.101613. [Epub ahead of print] PubMed PMID: 32126292.	No appropriate population sample
118: COVID-19 National Incident Room Surveillance Team. COVID-19, Australia: Epidemiology Report 5 (Reporting week ending 19:00 AEDT 29 February 2020). Commun Dis Intell (2018). 2020 Mar 4;44. doi: 10.33321/cdi.2020.44.20. PubMed PMID: 32126197	No appropriate outcome
119: Wang CJ, Ng CY, Brook RH. Response to COVID-19 in Taiwan: Big Data Analytics, New Technology, and Proactive Testing. JAMA. 2020 Mar 3. doi: 10.1001/jama.2020.3151. [Epub ahead of print] PubMed PMID: 32125371.	No appropriate population sample
120: Amodio E, Vitale F, Cimino L, Casuccio A, Tramuto F. Outbreak of Novel Coronavirus (SARS-Cov-2): First Evidences From International Scientific Literature and Pending Questions. Healthcare (Basel). 2020 Feb 27;8(1). pii: E51. doi: 10.3390/healthcare8010051. PubMed PMID: 32120965.	No appropriate population sample
121: Wang X, Zhou Z, Zhang J, Zhu F, Tang Y, Shen X. A case of 2019 Novel Coronavirus in a pregnant woman with preterm delivery. Clin Infect Dis. 2020 Feb 28. pii: ciaa200. doi: 10.1093/cid/ciaa200. [Epub ahead of print] PubMed PMID: 32119083	Included

122: Wang D, Ju XL, Xie F, Lu Y, Li FY, Huang HH, Fang XL, Li YJ, Wang JY, Yi B, Yue JX, Wang J, Wang LX, Li B, Wang Y, Qiu BP, Zhou ZY, Li KL, Sun JH, Liu XG, Li GD, Wang YJ, Cao AH, Chen YN. [Clinical analysis of 31 cases of 2019 novel coronavirus infection in children from six provinces (autonomous region) of northern China]. Zhonghua Er Ke Za Zhi. 2020 Mar 2;58(4):E011. doi: 10.3760/cma.j.cn112140-20200225-00138. [Epub ahead of print] Chinese. PubMedPMID: 32118389.	No appropriate population sample
123: Henwood AF. Coronavirus disinfection in histopathology. J Histotechnol. 2020 Mar 1:1-3. doi: 10.1080/01478885.2020.1734718. [Epub ahead of print] PubMed PMID: 32116147	No appropriate outcome
124: Lu Q, Shi Y. Coronavirus disease (COVID-19) and neonate: What neonatologist need to know. J Med Virol. 2020 Mar 1. doi: 10.1002/jmv.25740. [Epub ahead of print] Review. PubMed PMID: 32115733.	No original article
125: Chen S, Huang B, Luo DJ, Li X, Yang F, Zhao Y, Nie X, Huang BX. [Pregnant women with new coronavirus infection: a clinical characteristics and placental pathological analysis of three cases]. Zhonghua Bing Li Xue Za Zhi. 2020 Mar 1;49(0):E005. doi: 10.3760/cma.j.cn112151-20200225-00138. [Epub ahead of print] Chinese. PubMed PMID: 32114744.	Included
126: Rothan HA, Byrareddy SN. The epidemiology and pathogenesis of coronavirus disease (COVID-19) outbreak. J Autoimmun. 2020 Feb 26:102433. doi: 10.1016/j.jaut.2020.102433. [Epub ahead of print] Review. PubMed PMID: 32113704.	No appropriate outcome
127: Chen L, Xiong J, Bao L, Shi Y. Convalescent plasma as a potential therapy for COVID-19. Lancet Infect Dis. 2020 Feb 27. pii: S1473-3099(20)30141-9. doi: 10.1016/S1473-3099(20)30141-9. [Epub ahead of print] PubMed PMID: 32113510.	No appropriate population sample

128: Ma XL, Chen Z, Zhu JJ, Shen XX, Wu MY, Shi LP, Du LZ, Fu JF, Shu Q. Management strategies of neonatal jaundice during the coronavirus disease 2019 outbreak. World J Pediatr. 2020 Feb 28. doi: 10.1007/s12519-020-00347-3. [Epub ahead of print] Review. PubMed PMID: 32112336.	No appropriate population sample
129: Kam KQ, Yung CF, Cui L, Lin Tzer Pin R, Mak TM, Maiwald M, Li J, Chong CY, Nadua K, Tan NWH, Thoon KC. A Well Infant with Coronavirus Disease 2019 (COVID-19) with High Viral Load. Clin Infect Dis. 2020 Feb 28. pii: ciaa201. doi: 10.1093/cid/ciaa201. [Epub ahead of print] PubMed PMID: 32112082.	No appropriate population sample
130: Cai J, Xu J, Lin D, Yang Z, Xu L, Qu Z, Zhang Y, Zhang H, Jia R, Liu P, Wang X, Ge Y, Xia A, Tian H, Chang H, Wang C, Li J, Wang J, Zeng M. A Case Series of children with 2019 novel coronavirus infection: clinical and epidemiological features. Clin Infect Dis. 2020 Feb 28. pii: ciaa198. doi: 10.1093/cid/ciaa198. [Epub ahead of print] PubMed PMID: 32112072.	No appropriate population sample
131: Huang WH, Teng LC, Yeh TK, Chen YJ, Lo WJ, Wu MJ, Chin CS, Tsan YT, Lin TC, Chai JW, Lin CF, Tseng CH, Liu CW, Wu CM, Chen PY, Shi ZY, Liu PY. 2019 novel coronavirus disease (COVID-19) in Taiwan: Reports of two cases from Wuhan, China. J Microbiol Immunol Infect. 2020 Feb 19. pii: S1684-1182(20)30037-2. doi: 10.1016/j.jmii.2020.02.009. [Epub ahead of print] PubMed PMID: 32111449.	No appropriate population sample
132: Liu M, Xu HL, Yuan M, Liu ZR, Wu XY, Zhang Y, Ma LY, Gong L, Gan H, Zong WW, Tao SM, Liu Q, Du YN, Tao FB. [Analysis on epidemic situation and spatiotemporal changes of COVID-19 in Anhui]. Zhonghua Yu Fang Yi Xue Za Zhi. 2020 Feb 27;54(0):E019. doi: 10.3760/cma.j.cn112150-20200221-00150. [Epub ahead of print] Chinese. PubMed PMID: 32107910.	No appropriate outcome

133: Rasmussen SA, Smulian JC, Lednicky JA, Wen TS, Jamieson DJ. Coronavirus disease 2019 (COVID 19) and pregnancy: what obstetricians need to know. Am J Obstet Gynecol. 2020 Feb 24. pii: S0002-9378(20)30197-6. doi: 10.1016/j.ajog.2020.02.017. [Epub ahead of print] Review. PubMed PMID: 32105680.	No original article
134: Cai JH, Wang XS, Ge YL, Xia AM, Chang HL, Tian H, Zhu YX, Wang QR, Zeng JS. [First case of 2019 novel coronavirus infection in children in Shanghai]. Zhonghua Er Ke Za Zhi. 2020 Feb 2;58(2):86-87. doi: 10.3760/cma.j.issn.0578-1310.2020.02.002. Chinese. PubMed PMID: 32102141.	No appropriate population sample
135: Fang F, Luo XP. [Facing the pandemic of 2019 novel coronavirus infections: the pediatric perspectives]. Zhonghua Er Ke Za Zhi. 2020 Feb 2;58(2):81-85. doi: 10.3760/cma.j.issn.0578-1310.2020.02.001. Chinese. PubMed PMID: 32102140.	No appropriate population sample
136: Lai A, Bergna A, Acciarri C, Galli M, Zehender G. Early phylogenetic estimate of the effective reproduction number of SARS-CoV-2. J Med Virol. 2020 Feb 25. doi: 10.1002/jmv.25723. [Epub ahead of print] PubMed PMID: 32096566.	No appropriate population sample
137: Ralph R, Lew J, Zeng T, Francis M, Xue B, Roux M, Toloue Ostadgavahi A, Rubino S, Dawe NJ, Al-Ahdal MN, Kelvin DJ, Richardson CD, Kindrachuk J, Falzarano D, Kelvin AA. 2019-nCoV (Wuhan virus), a novel Coronavirus: human-to-human transmission, travel-related cases, and vaccine readiness. J Infect Dev Ctries. 2020 Jan 31;14(1):3-17. doi: 10.3855/jidc.12425. PubMed PMID: 32088679.	No appropriate population sample
138: Gilbert M, Pullano G, Pinotti F, Valdano E, Poletto C, Boëlle PY, D'Ortenzio E, Yazdanpanah Y, Eholie SP, Altmann M, Gutierrez B, Kraemer MUG, Colizza V. Preparedness and vulnerability of African countries against importations of COVID-19: a modelling study. Lancet. 2020 Mar 14;395(10227):871-877. doi: 10.1016/S0140-	No appropriate population sample

6736(20)30411-6. Epub 2020 Feb 20. PubMed PMID: 32087820.	
139: Catton H. Global challenges in health and health care for nurses and midwives everywhere. Int Nurs Rev. 2020 Mar;67(1):4-6. doi: 10.1111/inr.12578. PubMed PMID: 32083728.	No appropriate outcome
140: Kim JY, Ko JH, Kim Y, Kim YJ, Kim JM, Chung YS, Kim HM, Han MG, Kim SY, Chin BS. Viral Load Kinetics of SARS-CoV-2 Infection in First Two Patients in Korea. J Korean Med Sci. 2020 Feb 24;35(7):e86. doi: 10.3346/jkms.2020.35.e86. PubMed PMID: 32080991; PubMed Central PMCID: PMC7036338.	No appropriate population sample
141: Xu XW, Wu XX, Jiang XG, Xu KJ, Ying LJ, Ma CL, Li SB, Wang HY, Zhang S, Gao HN, Sheng JF, Cai HL, Qiu YQ, Li LJ. Clinical findings in a group of patients infected with the 2019 novel coronavirus (SARS-Cov-2) outside of Wuhan, China: retrospective case series. BMJ. 2020 Feb 19;368:m606. doi: 10.1136/bmj.m606. Erratum in: BMJ. 2020 Feb 27;368:m792. PubMed PMID: 32075786.	No appropriate population sample
142: Han W, Quan B, Guo Y, Zhang J, Lu Y, Feng G, Wu Q, Fang F, Cheng L, Jiao N, Li X, Chen Q. The course of clinical diagnosis and treatment of a case infected with coronavirus disease 2019. J Med Virol. 2020 May;92(5):461-463. doi: 10.1002/jmv.25711. Epub 2020 Mar 1. PubMed PMID: 32073161.	No appropriate population sample
143: Cheng ZJ, Shan J. 2019 Novel coronavirus: where we are and what we know. Infection. 2020 Feb 18. doi: 10.1007/s15010-020-01401-y. [Epub ahead of print] Review. PubMed PMID: 32072569.	No original article

144: Zeng LK, Tao XW, Yuan WH, Wang J, Liu X, Liu ZS. [First case of neonate infected with novel coronavirus pneumonia in China]. Zhonghua Er Ke Za Zhi. 2020 Feb 17;58(0):E009. doi: 10.3760/cma.j.issn.0578-1310.2020.0009. [Epub ahead of print] Chinese. PubMed PMID: 32065520.	Included
145: Wang XF, Yuan J, Zheng YJ, Chen J, Bao YM, Wang YR, Wang LF, Li H, Zeng JX, Zhang YH, Liu YX, Liu L. [Retracted: Clinical and epidemiological characteristics of 34 children with 2019 novel coronavirus infection in Shenzhen]. Zhonghua Er Ke Za Zhi. 2020 Feb 17;58(0):E008. doi: 10.3760/cma.j.issn.0578-1310.2020.0008. [Epub ahead of print] Chinese. PubMed PMID: 32062875.	No appropriate population sample
146: Feng K, Yun YX, Wang XF, Yang GD, Zheng YJ, Lin CM, Wang LF. [Analysis of CT features of 15 Children with 2019 novel coronavirus infection]. Zhonghua Er Ke Za Zhi. 2020 Feb 16;58(0):E007. doi: 10.3760/cma.j.issn.0578-1310.2020.0007. [Epub ahead of print] Chinese. PubMed PMID: 32061200.	No appropriate population sample
147: Wei M, Yuan J, Liu Y, Fu T, Yu X, Zhang ZJ. Novel Coronavirus Infection in Hospitalized Infants Under 1 Year of Age in China. JAMA. 2020 Feb 14. doi: 10.1001/jama.2020.2131. [Epub ahead of print] PubMed PMID: 32058570; PubMed Central PMCID: PMC7042807.	No appropriate population sample
148: Jiang X, Rayner S, Luo MH. Does SARS-CoV-2 has a longer incubation period than SARS and MERS? J Med Virol. 2020 May;92(5):476-478. doi: 10.1002/jmv.25708. Epub 2020 Feb 24. PubMed PMID: 32056235.	No appropriate population sample
149: Bajema KL, Oster AM, McGovern OL, Lindstrom S, Stenger MR, Anderson TC, Isenhour C, Clarke KR, Evans ME, Chu VT, Biggs HM, Kirking HL, Gerber SI, Hall AJ, Fry AM, Oliver SE; 2019-nCoV Persons Under Investigation Team; 2019-CoV Persons Under Investigation Team. Persons Evaluated for 2019 Novel Coronavirus - United States,	No appropriate population sample

January 2020. MMWR Morb Mortal Wkly Rep. 2020 Feb 14;69(6):166-170. doi: 10.15585/mmwr.mm6906e1. PubMed PMID: 32053579; PubMedCentral PMCID: PMC7017962.	
150: Zhang L, Liu Y. Potential interventions for novel coronavirus in China: A systematic review. J Med Virol. 2020 May;92(5):479-490. doi: 10.1002/jmv.25707. Epub 2020 Mar 3. Review. PubMed PMID: 32052466.	No original article
151: Pediatric Branch of Hubei Medical Association; Pediatric Branch of Wuhan Medical Association; Pediatric Medical Quality Control Center of Hubei. [Recommendation for the diagnosis and treatment of novel coronavirus infection in children in Hubei (Trial version 1)]. Zhongguo Dang Dai Er Ke Za Zhi. 2020 Feb;22(2):96-99. Chinese. PubMed PMID: 32051073.	No appropriate population sample
152: Pediatric Committee, Medical Association of Chinese People's Liberation Army; Editorial Committee of Chinese Journal of Contemporary Pediatrics. [Emergency response plan for the neonatal intensive care unit during epidemic of 2019 novel coronavirus]. Zhongguo Dang Dai Er Ke Za Zhi. 2020 Feb;22(2):91-95. Chinese. PubMed PMID: 32051072.	No appropriate outcome
153: Working Group for the Prevention and Control of Neonatal 2019-nCoV Infection in the Perinatal Period of the Editorial Committee of Chinese Journal of Contemporary Pediatrics. [Perinatal and neonatal management plan for prevention and control of 2019 novel coronavirus infection (1st Edition)]. Zhongguo Dang Dai Er Ke Za Zhi. 2020 Feb;22(2):87-90. Chinese. PubMed PMID: 32051071.	No original article
154: Schwartz DA, Graham AL. Potential Maternal and Infant Outcomes from (Wuhan) Coronavirus 2019-nCoV Infecting Pregnant Women: Lessons from SARS, MERS, and Other Human Coronavirus Infections.	No original article

Viruses. 2020 Feb 10;12(2). pii: E194. doi: 10.3390/v12020194. PubMed PMID: 32050635; PubMed Central PMCID: PMC7077337.	
155: Liu Y, Yang Y, Zhang C, Huang F, Wang F, Yuan J, Wang Z, Li J, Li J, Feng C, Zhang Z, Wang L, Peng L, Chen L, Qin Y, Zhao D, Tan S, Yin L, Xu J, Zhou C, Jiang C, Liu L. Clinical and biochemical indexes from 2019-nCoV infected patients linked to viral loads and lung injury. Sci China Life Sci. 2020 Mar;63(3):364-374. doi: 10.1007/s11427-020-1643-8. Epub 2020 Feb 9. PubMed PMID: 32048163	No appropriate population sample
156: Chen F, Liu ZS, Zhang FR, Xiong RH, Chen Y, Cheng XF, Wang WY, Ren J. [First case of severe childhood novel coronavirus pneumonia in China]. Zhonghua Er Ke Za Zhi. 2020 Feb 11;58(0):E005. doi: 10.3760/cma.j.issn.0578-1310.2020.0005. [Epub ahead of print] Chinese. PubMed PMID: 32045966.	No appropriate population sample
157: Lee PI, Hsueh PR. Emerging threats from zoonotic coronaviruses-from SARS and MERS to 2019-nCoV. J Microbiol Immunol Infect. 2020 Feb 4. pii: S1684-1182(20)30011-6. doi: 10.1016/j.jmii.2020.02.001. [Epub ahead of print] PubMed PMID: 32035811.	No appropriate outcome
158: Favre G, Pomar L, Musso D, Baud D. 2019-nCoV epidemic: what about pregnancies? Lancet. 2020 Feb 22;395(10224):e40. doi: 10.1016/S0140-6736(20)30311-1. Epub 2020 Feb 6. PubMed PMID: 32035511.	No original article
159: Shen K, Yang Y, Wang T, Zhao D, Jiang Y, Jin R, Zheng Y, Xu B, Xie Z, Lin L, Shang Y, Lu X, Shu S, Bai Y, Deng J, Lu M, Ye L, Wang X, Wang Y, Gao L; China National Clinical Research Center for Respiratory Diseases; National Center for Children's Health, Beijing, China; Group of Respirology, Chinese Pediatric Society, Chinese Medical Association; Chinese Medical Doctor Association Committee on Respirology	No original article

Pediatrics; China Medicine Education AssociationCommittee on Pediatrics; Chinese Research Hospital Association Committee on Pediatrics; Chinese Non-government Medical Institutions Association Committee on Pediatrics; China Association of Traditional Chinese Medicine, Committee on Children's Health and Medicine Research; China News of Drug Information Association, Committee on Children's Safety Medication; Global Pediatric Pulmonology Alliance. Diagnosis, treatment, and prevention of 2019 novel coronavirus infection in children: experts' consensus statement. World J Pediatr. 2020 Feb 7. doi: 10.1007/s12519-020-00343-7. [Epub ahead of print] PubMed PMID:32034659	
160: Chen ZM, Fu JF, Shu Q, Chen YH, Hua CZ, Li FB, Lin R, Tang LF, Wang TL, Wang W, Wang YS, Xu WZ, Yang ZH, Ye S, Yuan TM, Zhang CM, Zhang YY. Diagnosis and treatment recommendations for pediatric respiratory infection caused by the 2019 novel coronavirus. World J Pediatr. 2020 Feb 5. doi: 10.1007/s12519-020-00345-5. [Epub ahead of print] Review. PubMed PMID: 32026148.	No original article
162: Kanne JP. Chest CT Findings in 2019 Novel Coronavirus (2019-nCoV) Infections from Wuhan, China: Key Points for the Radiologist. Radiology. 2020 Apr;295(1):16-17. doi: 10.1148/radiol.2020200241. Epub 2020 Feb 4. PubMed PMID: 32017662	No appropriate population sample
163: Xu X, Chen P, Wang J, Feng J, Zhou H, Li X, Zhong W, Hao P. Evolution of the novel coronavirus from the ongoing Wuhan outbreak and modeling of its spike protein for risk of human transmission. Sci China Life Sci. 2020 Mar;63(3):457-460. doi: 10.1007/s11427-020-1637-5. Epub 2020 Jan 21. PubMed PMID: 32009228	No appropriate population sample
164: Chan JF, Yuan S, Kok KH, To KK, Chu H, Yang J, Xing F, Liu J, Yip CC, Poon RW, Tsoi HW, Lo SK, Chan KH, Poon VK, Chan WM, Ip JD, Cai JP, Cheng VC, Chen H, Hui CK, Yuen KY. A familial cluster of pneumonia associated with the 2019 novel coronavirus indicating person-to-person transmission: a	No appropriate population sample

study of a family cluster. Lancet. 2020 Feb 15;395(10223):514-523. doi: 10.1016/S0140-6736(20)30154-9. Epub 2020 Jan 24. PubMed PMID: 31986261.	
165: Zhang M, Li W, Zhou P, Liu D, Luo R, Jongkaewwattana A, He Q. Genetic manipulation of porcine deltacoronavirus reveals insights into NS6 and NS7 functions: a novel strategy for vaccine design. Emerg Microbes Infect. 2019 Dec 20;9(1):20-31. doi: 10.1080/22221751.2019.1701391. eCollection 2020. PubMed PMID: 31859605; PubMed Central PMCID: PMC6968670.	No appropriate outcome
166: Cao L, Wang TY, Chen HZ, Qian Y, Chen BW, Fang P, Sun YX, Zhu RN, Deng J, Zhao LQ, Mi J, Zhang T. [A preliminary investigation on the serological and epidemiological characteristics of severe acute respiratory syndrome in children]. Zhonghua Er Ke Za Zhi. 2004 Nov;42(11):840-4. Chinese. PubMed PMID: 15631713	No appropriate population sample

3. Other sources

Song L, Xiao W, Ling K, Yao S and Chen X. Anesthetic Management for Emergent Cesarean Delivery in a Parturient with Recent Diagnosis of Coronavirus Disease 2019 (COVID-19): A Case Report. Transl Perioper & Pain Med 2020; 7 (3)	Included
Ferrazzi E, Frigerio L, Savasi V, Vergani P, Prefumo F, Barresi S. Mode of delivery and clinical findings in COVID-19-infected pregnant women in Northern Italy. (In press).	Included
Sara N. Iqbal, Rachael Overcash, Neggin Mokhtari, Haleema Saeed, Stacey Gold, Tamika Auguste, Muhammad-Usman Mirza, Maria-Elena Ruiz, Joeffrey J. Chahine, Masashi Waga, Glenn Wortmann.	Included

An Uncomplicated Delivery in a Patient with Covid-19 in the United States. N Engl J Med. 2020 Apr 1 : NEJMc2007605. Published online 2020 Apr 1. doi: 10.1056/NEJMc2007605	
Breslin N, Baptiste C, Miller R, Fuchs K, Goffman D, Gyamfi-Bannerman C, D'Alton M, COVID-19 in pregnancy: early lessons, American Journal of Obstetrics & Gynecology MFM (2020), doi: https://doi.org/10.1016/j.ajogmf.2020.100111.	Overlapping data
Liu W, Wang Q, Zhang Q, Chen L, Chen J, Zhang B. et al. Coronavirus disease 2019 (COVID-19) during pregnancy: A case series. Preprints 2020;2020020373. Available from: https://www.preprints.org/manuscript/202002.0373/v1	Overlapping data

pathogens

MDPI

PRISMA Checklist

Section/topic	#	Checklist item	Reported on page #
**TITLE**			
Title	1	Identify the report as a systematic review, meta-analysis, or both.	1
**ABSTRACT**			
Structured summary	2	Provide a structured summary including, as applicable: background; objectives; data sources; study eligibility criteria, participants, and interventions; study appraisal and synthesis methods; results; limitations; conclusions and implications of key findings; systematic review registration number.	1
**INTRODUCTION**			
Rationale	3	Describe the rationale for the review in the context of what is already known.	1-2
Objectives	4	Provide an explicit statement of questions being addressed with reference to participants, interventions, comparisons, outcomes, and study design (PICOS).	1-2
**METHODS**			
Protocol and registration	5	Indicate if a review protocol exists, if and where it can be accessed (e.g., Web address), and, if available, provide registration information including registration number.	NA
Eligibility criteria	6	Specify study characteristics (e.g., PICOS, length of follow-up) and report characteristics (e.g., years considered, language, publication status) used as criteria for eligibility, giving rationale.	3
Information sources	7	Describe all information sources (e.g., databases with dates of coverage, contact with study authors to identify additional studies) in the search and date last searched.	2
Search	8	Present full electronic search strategy for at least one database, including any limits used, such that it could be repeated.	2
Study selection	9	State the process for selecting studies (i.e., screening, eligibility, included in systematic review, and, if applicable, included in the meta-analysis).	3
Data collection process	10	Describe method of data extraction from reports (e.g., piloted forms, independently, in duplicate) and any processes for obtaining and confirming data from investigators.	3
Data items	11	List and define all variables for which data were sought (e.g., PICOS, funding sources) and any assumptions and simplifications made.	3
Risk of bias in individual studies	12	Describe methods used for assessing risk of bias of individual studies (including specification of whether this was done at the study or outcome level), and how this information is to be used in any data synthesis.	3
Summary measures	13	State the principal summary measures (e.g., risk ratio, difference in means).	NA
Synthesis of results	14	Describe the methods of handling data and combining results of studies, if done, including measures of consistency (e.g., I^2) for each meta-analysis.	3
Section/topic	#	Checklist item	Reported on page #
Risk of bias across studies	15	Specify any assessment of risk of bias that may affect the cumulative evidence (e.g., publication bias, selective reporting within studies).	NA
Additional analyses	16	Describe methods of additional analyses (e.g., sensitivity or subgroup analyses, meta-regression), if done, indicating which were pre-specified.	NA
**RESULTS**			
Study selection	17	Give numbers of studies screened, assessed for eligibility, and included in the review, with reasons for exclusions at each stage, ideally with a flow diagram.	5

Study characteristics	18	For each study, present characteristics for which data were extracted (e.g., study size, PICOS, follow-up period) and provide the citations.	5-12
Risk of bias within studies	19	Present data on risk of bias of each study and, if available, any outcome level assessment (see item 12).	12-14
Results of individual studies	20	For all outcomes considered (benefits or harms), present, for each study: (a) simple summary data for each intervention group (b) effect estimates and confidence intervals, ideally with a forest plot.	14-23
Synthesis of results	21	Present results of each meta-analysis done, including confidence intervals and measures of consistency.	NA
Risk of bias across studies	22	Present results of any assessment of risk of bias across studies (see Item 15).	NA
Additional analysis	23	Give results of additional analyses, if done (e.g., sensitivity or subgroup analyses, meta-regression [see Item 16]).	NA
**DISCUSSION**			
Summary of evidence	24	Summarize the main findings including the strength of evidence for each main outcome; consider their relevance to key groups (e.g., healthcare providers, users, and policy makers).	23-25
Limitations	25	Discuss limitations at study and outcome level (e.g., risk of bias), and at review-level (e.g., incomplete retrieval of identified research, reporting bias).	25
Conclusions	26	Provide a general interpretation of the results in the context of other evidence, and implications for future research.	25
**FUNDING**			
Funding	27	Describe sources of funding for the systematic review and other support (e.g., supply of data); role of funders for the systematic review.	26
